# Supplementary material for: Music to prevent deliriUm during neuroSurgerY (MUSYC): a single-centre, prospective randomised controlled trial
Source: BMJ Open. 2023 Jun 27;13(6):e069957. doi: 10.1136/bmjopen-2022-069957 (PMC10410844; doi:10.1136/bmjopen-2022-069957)
Supplement: Supplementary data [file bmjopen-2022-069957supp002.pdf]

Supplementary figure 1. Study course MUSYC trial

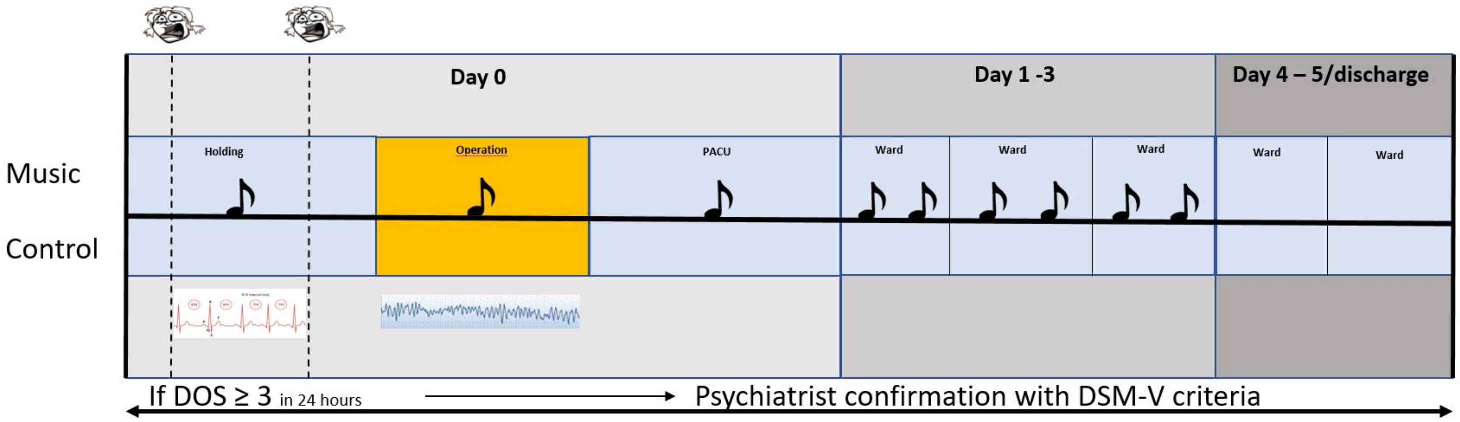

Course trial: after randomization, both groups receive ECG recording and two times anxiety measures. Intra-operative BIS registration is applied for depth of anesthesia. All participating subjects are treated according to standard care and delirium is measured until day 5 or discharge. Participants in the intervention group additionally receive music intervention 30 minutes before surgery, intra-operative during the entire operation, 30 minutes after surgery and twice per day 30minutes until post-operative day 3.
