# Supplementary material for: Music to prevent deliriUm during neuroSurgerY (MUSYC): a single-centre, prospective randomised controlled trial
Source: BMJ Open. 2023 Jun 27;13(6):e069957. doi: 10.1136/bmjopen-2022-069957 (PMC10410844; doi:10.1136/bmjopen-2022-069957)
Supplement: Supplementary data [file bmjopen-2022-069957supp003.pdf]

Supplementary table 1. Adherence to music intervention during trial

	Day 0			Day 1		Day 2		Day 3	
**Moment[1]**	**Pre**	**Per**	**Post**	**Morning**	**Noon**	**Morning**	**Noon**	**Morning**	**Noon**
**N[2]**	91	91	91	91	91	88	88	70	70
**Music**									
**No**	3	0	21	25	26	26	21	19	20
**Yes**	79	91	61	59	45	44	39	24	18
**Unknown[3]**	9	0	9	7	20	18	28	27	32
**Adherence[4]**	96%	100%	74%	70%	63%	63%	65%	56%	47%

Patients in the mITT population allocated to the music group. 1. Session of 30 minutes in the morning or afternoon. 2. Amount of patients in the musical group not discharged from the ward. 3. Unregistered music session. 4. Adherence calculated from the registered patients (i.e. 'Yes'/'No' + 'Yes')
