# Supplementary material for: Music to prevent deliriUm during neuroSurgerY (MUSYC): a single-centre, prospective randomised controlled trial
Source: BMJ Open. 2023 Jun 27;13(6):e069957. doi: 10.1136/bmjopen-2022-069957 (PMC10410844; doi:10.1136/bmjopen-2022-069957)
Supplement: Supplementary data [file bmjopen-2022-069957supp004.pdf]

Supplementary figure 2. Course delirium diagnosis

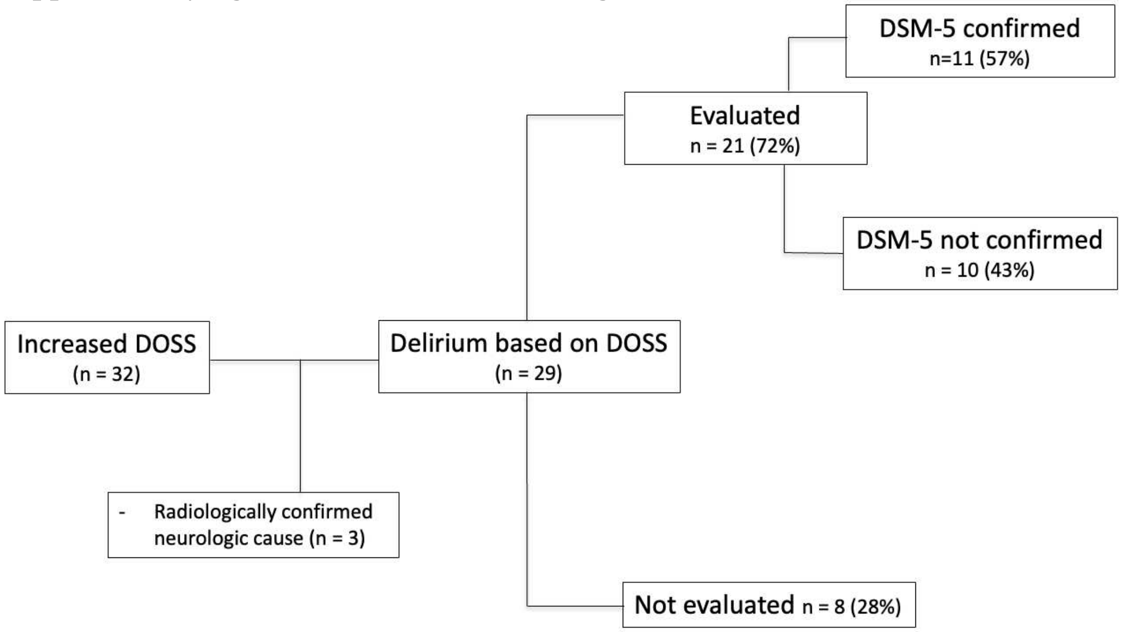

Delirium diagnosis flowchart, diagnosis of delirium required a two-step procedure. First, all participants were screened with the DOS scale. Second, in case of a daily mean score of 3 or higher (and no neurologic cause was radiologically found) a psychiatrist was consulted to assess the clinical diagnosis of delirium based on the DSM-5 criteria. In 8 patients, the psychiatrist was not consulted due to logistic issues.
