# Supplementary material for: Music to prevent deliriUm during neuroSurgerY (MUSYC): a single-centre, prospective randomised controlled trial
Source: BMJ Open. 2023 Jun 27;13(6):e069957. doi: 10.1136/bmjopen-2022-069957 (PMC10410844; doi:10.1136/bmjopen-2022-069957)
Supplement: Author's manuscript [file bmjopen-2022-069957.draft_revisions.pdf]

BMJ Open

BMJ Open is committed to open peer review. As part of this commitment we make the peer review history of every article we publish publicly available.

When an article is published we post the peer reviewers' comments and the authors' responses online. We also post the versions of the paper that were used during peer review. These are the versions that the peer review comments apply to.

The versions of the paper that follow are the versions that were submitted during the peer review process. They are not the versions of record or the final published versions. They should not be cited or distributed as the published version of this manuscript.

BMJ Open is an open access journal and the full, final, typeset and author-corrected version of record of the manuscript is available on our site with no access controls, subscription charges or pay-per-view fees (http://bmjopen.bmj.com).

If you have any questions on BMJ Open's open peer review process please email info.bmjopen@bmj.com

BMJ Open

# Music to prevent delirium during neurosurgery (MUSYC): a single-centre prospective randomised controlled trial.

| | |
|---|---|
| Journal: | *BMJ Open* |
| Manuscript ID | bmjopen-2022-069957 |
| Article Type: | Original research |
| Date Submitted by the Author: | 10-Nov-2022 |
| Complete List of Authors: | Kappen, Pablo; Erasmus Medical Center, Department of Neurosurgery; Erasmus Medical Center, Department of Neuroscience<br>Mos, M.I.; Erasmus Medical Center, Department of Neurosurgery; Erasmus Medical Center, Department of Neuroscience<br>Jeekel, Johannes; Erasmus Medical Center, Department of Neuroscience<br>Dirven, Clemens; Erasmus Medical Center, Department of Neurosurgery<br>Kushner, Steven; Erasmus Medical Center, Department of Psychiatry<br>Osse, Robert-Jan; Erasmus Medical Center, Department of Psychiatry<br>Coesmans, Michiel; Erasmus Medical Center, Department of Psychiatry<br>Poley, Marten; Erasmus Medical Center, Institute for Medical Technology Assessment (iMTA); Erasmus Medical Center, Department of Paediatric Surgery and Intensive Care<br>van Schie, Mathijs; Erasmus Medical Center, Department of Cardiology<br>van der Holt, Bronno; Erasmus Medical Center, Department of Haematology<br>Klimek, M; Erasmus Medical Center, Anesthesiology<br>Vincent, Arnaud; Erasmus Medical Center, Department of Neurosurgery |
| Keywords: | Neurosurgery < SURGERY, Delirium & cognitive disorders < PSYCHIATRY, COMPLEMENTARY MEDICINE, Clinical trials < THERAPEUTICS, PREVENTIVE MEDICINE, NEUROSURGERY |
| | |

SCHOLARONE™
Manuscripts

*I, the Submitting Author has the right to grant and does grant on behalf of all authors of the Work (as defined in the below author licence), an exclusive licence and/or a non-exclusive licence for contributions from authors who are: i) UK Crown employees; ii) where BMJ has agreed a CC-BY licence shall apply, and/or iii) in accordance with the terms applicable for US Federal Government officers or employees acting as part of their official duties; on a worldwide, perpetual, irrevocable, royalty-free basis to BMJ Publishing Group Ltd ("BMJ") its licensees and where the relevant Journal is co-owned by BMJ to the co-owners of the Journal, to publish the Work in this journal and any other BMJ products and to exploit all rights, as set out in our licence.*

*The Submitting Author accepts and understands that any supply made under these terms is made by BMJ to the Submitting Author unless you are acting as an employee on behalf of your employer or a postgraduate student of an affiliated institution which is paying any applicable article publishing charge ("APC") for Open Access articles. Where the Submitting Author wishes to make the Work available on an Open Access basis (and intends to pay the relevant APC), the terms of reuse of such Open Access shall be governed by a Creative Commons licence – details of these licences and which Creative Commons licence will apply to this Work are set out in our licence referred to above.*

*Other than as permitted in any relevant BMJ Author's Self Archiving Policies, I confirm this Work has not been accepted for publication elsewhere, is not being considered for publication elsewhere and does not duplicate material already published. I confirm all authors consent to publication of this Work and authorise the granting of this licence.*

For peer review only

# Music to prevent delirium during neurosurgery (MUSYC): a single-centre prospective randomised controlled trial.

P.R. Kappen[✉, 1, 2], M.I. Mos[1, 2], J. Jeekel[2], C.M.F. Dirven[1], S.A. Kushner[3], R.J. Osse[3], M. Coesmans[3], M.J. Poley[4, 5], M. van Schie[6], B. van der Holt[7], M. Klimek[8], A.J.P.E. Vincent[1]

**Affiliations.**

✉ Corresponding Author; Mr. Pablo Kappen, address: Doctor Molewaterplein 40, 3015 GD Rotterdam, the Netherlands, Room NA-2124, phone: +31(0)10 704 35 41, email: p.kappen@erasmusmc.nl

1. Department of Neurosurgery, Erasmus MC University Medical Center, Rotterdam, the Netherlands
2. Department of Neuroscience, Erasmus MC University Medical Center, Rotterdam, the Netherlands
3. Department of Psychiatry, Erasmus MC University Medical Center, Rotterdam, the Netherlands
4. Institute for Medical Technology Assessment (iMTA), Erasmus MC University Medical Center, Rotterdam, the Netherlands
5. Department of Paediatric Surgery and Intensive Care, Sophia Children's Hospital, Erasmus MC University Medical Center, Rotterdam, the Netherlands
6. Department of Cardiology, Erasmus MC University Medical Center, Rotterdam, the Netherlands
7. Department of Haematology, Erasmus Medical Center Cancer Institute, Rotterdam, the Netherlands
8. Department of Anaesthesiology, Erasmus Medical Center, Rotterdam, the Netherlands

For peer review only

**Abstract word count:** 250

**Manuscript word count:** 3500

**Reference count:** 40

**Number of figures:** 4

**Number of tables:** 3



For peer review only - http://bmjopen.bmj.com/site/about/guidelines.xhtml

**Bullet points**

- Music reduced the onset of delirium after craniotomy, as confirmed with DOSS but not with DSM-5
- Music activated pre-operative heart rate variability, a marker of autonomic tone
- A deeper level of anesthesia in the music group was achieved with standardized dosages of sedation.
- A beneficial reducing trend of music for pre-operative anxiety, but no effect on post-operative complications, length of stay and location of discharge were found

For peer review only



For peer review only - http://bmjopen.bmj.com/site/about/guidelines.xhtml

**Abstract**

**Objectives**

Delirium is a serious complication following neurosurgical procedures, as high incidence rates and associations with increased length of hospital stay, number of re-operations and mortality are reported. We hypothesize that the beneficial effect of music on a combination of delirium-eliciting factors might reduce delirium incidence following neurosurgery and subsequently improve clinical outcomes.

**Methods**

This single-centre prospective randomized controlled trial in adults compared the effects of music administered before, during and after craniotomy with standard of clinical care. The primary endpoint delirium was assessed by the delirium observation screening scale (DOSS) and confirmed by a psychiatrist according to DSM-5 criteria. A variety of secondary outcomes were assessed to substantiate the effects of music on delirium and its clinical implications.

**Results**

We enrolled 189 patients: delirium as assessed by the DOSS was less common in the music (n=95) than in the control group (n=94, OR:0.49, p=0.048). However, after DSM-5 confirmation, differences in delirium were not significant (OR:0.47, p=0.342). Moreover, music increased the heart rate variability (RMSSD, p=0.021 and HF/LF, p=0.025) and decreased depth of anaesthesia at 60 minutes (p=0.001), 75 minutes (p=0.022) and 90 minutes (p<0.001) with standardized dosages of anaesthesia. All other secondary outcomes were not different between groups.

**Conclusion**

Our results support the efficacy of music in preventing delirium after craniotomy, as found with DOSS but not after DSM-5 confirmation, substantiated by the effect of music on pre-operative autonomic tone and depth of anesthesia. Delirium screening tools should be validated and the long-term implications should be evaluated after craniotomy.

For peer review only

## WHAT IS ALREADY KNOWN ON THIS TOPIC

- Pilot studies suggest promising effects of music in preventing delirium in surgical and intensive care unit patients.

## WHAT THIS STUDY ADDS

- This efficacy trial showed that music prevented delirium after craniotomy when defined by DOSS, but not with DSM-5 criteria.

## HOW THIS STUDY MIGHT AFFECT RESEARCH, PRACTICE OR POLICY

- Our results fit well within the current literature and support the implementation of music for the prevention of delirium also within the neurosurgical population.

For peer review only



For peer review only - http://bmjopen.bmj.com/site/about/guidelines.xhtml

## Introduction

Delirium, is a common clinical syndrome in the elderly, and is defined as an acute disorder of awareness, attention and cognition. Delirium is often multifactorial in aetiology and can be influenced by a number of predisposing (e.g. older age, cognitive impairment, multiple comorbidities) and precipitating factors (e.g. infections, operations, drugs).(1)

Delirium in neurosurgical patients has been reported in 4 to 44% of cases, with a large variation in definition and assessment methods. (2) The high incidence in this population is probably caused by the underlying massive neuro-inflammation which is usually induced during intracranial procedures. (3) The clinical relevance of delirium in neurosurgery remains difficult to assess objectively, as criteria for delirium overlap with symptoms from the primary neurologic injury. However, delirium independently predicted clinical outcomes in neurosurgical and neurocritically ill patients such as worse functional outcome (4), length of stay, costs and death. (5) These complications justify the search for preventive therapies for post-operative delirium in neurosurgical patients.

Although promising preventive approaches are emerging, pharmacological treatments have inconsistent results and are accompanied with side effects. (6, 7) Nonpharmacological multi-component approaches for primary prevention, such as reorientation, early mobilization, therapeutic activities, hydration, nutrition and sleep strategies have been shown to be effective and cost-reducing in other patient groups. However, these approaches can be labour intensive, and include the use of volunteers, or non-licensed professionals to enhance feasibility. (8)

Recorded music is an easy applicable intervention which neatly fits throughout the entire perioperative process and has been shown to be effective in the surgical population in reducing a combination of delirium-eliciting factors such as pre-operative anxiety, post-operative pain, stress response and opioid/sedation requirement.(9-17) A recent meta-analysis, evaluating six randomised pilot studies, found music potentially being effective in preventing postoperative delirium in postsurgical patients. However, these studies did not include neurosurgical patients. (18)

For peer review only

We therefore designed a randomized controlled trial to assess the effect of music in the prevention of post-operative delirium in neurosurgical patients.



For peer review only - http://bmjopen.bmj.com/site/about/guidelines.xhtml

## Methods

*Patient and public involvement*

Patients were involved in the composition of the music playlists, as these were based on their music preference, the role music plays in their life (ie, whether they are musician/ just listen to music) and the importance of music. The results of our trial were disseminated to the participating patients through a letter after publication.

*Study design*

The Music to prevent deliriUm during neuroSurgerY Clinical (MUSYC) trial was a single centre, prospective randomized controlled trial conducted at the Erasmus MC Rotterdam, the Netherlands. The trial compared effects of music administered before, during and after craniotomy with standard of clinical care. The Medical Ethics Review Committee of the Erasmus Medical Center, Rotterdam (Erasmus MC), declared this study not subject to the Medical Research Involving Human Subjects Act (i.e. 'Non-WMO'), and approved this study (MEC-2020-0064), on the 25th of February of 2020, as such.

The trial protocol was designed by neurosurgeons, psychiatrists, anaesthesiologists and neuroscientists and followed the Standard Protocol Items: Recommendations for Interventional Trials (SPIRIT) guidelines and the Consolidated Standards of Reporting Trials (CONSORT) guidelines for non-pharmacological treatments (see checklist in online supplementary material). The trial was registered (trialregister.nl: NL8503 and ClinicalTrials.gov: NCT04649450) and details of the protocol have been published previously. (19, 20)

*Patients*

Adult patients (i.e. age 18 years or more) undergoing craniotomy (i.e. opening the dura requiring bone flap removal) at the Erasmus MC with sufficient knowledge of the Dutch language were eligible for study participation. Exclusion criteria were: impaired awareness before surgery (i.e. motoric less than 6 in the Glasgow Coma Scale), planned post-operative intensive care unit (ICU) admission, suspected delirium (defined as fluctuating awareness) at baseline, antipsychotic treatment, undergoing surgery impeding supply of music, bilateral hearing impairment and participation in other clinical trials inferring with results. During



For peer review only - http://bmjopen.bmj.com/site/about/guidelines.xhtml

inclusion, one participant reported that music induced epileptic seizures (known as musicogenic epilepsy): this patient was excluded (and the exclusion criteria were adopted accordingly), as it was considered unethical to expose such a patient to music. Eligible patients were approached and written informed consent by patient or legal representative was obtained.

*Intervention*

All participating subjects were treated according to standard of care. Method of music intervention administration (i.e. type, frequency and duration) was applied based on previous studies.(12, 13) Participants in the intervention group (i.e. music group) received an over-ear headphone and a tablet with access to a platform with different pre-selected music playlists (i.e. jazz, blues, classic, electronic, pop, 60s, 70s, 80s etc), in which the music selection could be extended based on patients' wishes. These patients received the first 30 minutes of music the day of operation (day 0) while awaiting surgery (see supplementary figure 1 for 'Study Course MUSYC trial'). In the operating room, in-ear earphones after intubation, which were compatible with the Mayfield clamp and site of operation, were inserted and music was continued until just before detubation. After surgery, during recovery at the post-operative care unit (PACU), another 30 minutes of recorded music through over-ear headphones was administered. Finally, participants received 30 minutes of recorded music twice a day until post-operative day 3. Patients in the control group were asked to refrain from music listening, however this was not strictly controlled as this would influence the standard of clinical care too much. Nurses were instructed to monitor for music listening behaviour with a diary which was placed next to the music equipment. Periodically (approximately every six months) a training was given for all nurses on the ward to explain how music had to be administered and monitored.

*Outcome measures*

The primary outcome measure was presence or absence of postoperative delirium within the first five postoperative days. (21, 22) The diagnosis of delirium required a two-step procedure; first, all participating patients were daily screened by the treating nurse using the Delirium Observation Screening scale (DOSS), a validated 13-item delirium screening tool with higher scores indicating a higher probability of delirium. Use of the DOSS was already current practice at our department.(23-27) Second, in case of a daily mean score of 3 or



For peer review only - http://bmjopen.bmj.com/site/about/guidelines.xhtml

higher, which was radiologically not substantiated by a neurosurgical complication, a psychiatrist was consulted to assess the clinical diagnosis of delirium based on the Diagnostic and Statistical Manual of Mental Disorders (DSM)-5 criteria.(28) We chose not to blind the assessors from the intervention, as this could not be secured which might have led to misleading results.

Secondary outcomes were assessed to substantiate the effects of music on delirium and its clinical implications. Pre-operative secondary outcomes included anxiety (measured with the Visual Analogue Scale-anxiety/VAS-A) and heart rate variability (HRV), a marker of the autonomic tone reflecting parasympathetic nervous activity, measured with a 30 minutes electrocardiography (ECG) recording. The following HRV parameters were analysed: standard deviation of normal sinus beats (SDNN), root mean square of successive differences between normal heartbeats (RMSSD), the number of adjacent NN intervals that differ from each other by more than 50 ms (NN50) and the ratio of low frequency to high frequency power (HF/LF). Intra-operative secondary outcomes included depth of anaesthesia with Bispectral Index (BIS, Aspect ™ version 3.22) with standardized sedation dosages (propofol and remifentanil). BIS was measured from the non-operated side, if feasible with site of resection, and the anaesthesiologist was blinded from the intra-operative BIS values, which was considered ethical as this form monitoring is not standard of clinical care during intracranial procedures. Post-operative secondary outcomes were delirium severity (using the Delirium Rating Scale-revised-98/DRS-R-98) and delirium duration (onset until first day DOSS score <3), pain (Numeric Rating Scale/NRS pain and dosages analgesic), post-operative complications, length of stay and location of discharge. Finally, patients' satisfaction of receiving music was assessed with a 100- point visual analogue scale.

Baseline characteristics were extracted from questionnaires or the electronic patient file consisting of pre-hospital demographics (i.e. age, gender, medical history), in-hospital demographics (i.e. daily function with the Karnofsky Performance Scale/KPS or Modified Rankin scale/MRS and quality of life with the EQ-5D and EORTC QLQ – BN-20 and cognitive function with the Montreal Cognitive assessment / MoCA), disease characteristics (i.e. neurologic deficit, type and side of intracranial pathology) and operation details (i.e. emergency grade, duration of surgery).



For peer review only - http://bmjopen.bmj.com/site/about/guidelines.xhtml

*Statistical analysis*

We expected an incidence of delirium in our control group of 30%, which was based on the incidence of 24.2–32.4% documented in neurosurgical literature using the same screening tool (i.e. DOSS).(29, 30) When designing the trial, the expected effect could not be based on previous literature as no pooled effect of music on delirium was reported. Other non-pharmacological interventions mentioned a relative reduction of 36%–77% and we therefore considered the intervention clinically relevant if a relative reduction of 60%, corresponding to an absolute reduction of 18%, was achieved.(31, 32) Assuming a loss to follow-up of 5%, we estimated that a target sample size of 189 patients would provide the trial with a power of 80%.

Randomization was done in a 1:1 ratio, by a secured online software program (ALEA; FormsVision BV, Abcoude, The Netherlands) and stratified per type of disease characteristic (i.e. 'neuro-oncology', 'neurovascular', 'traumatic brain injury', 'infectious') and age (i.e. 'younger than 60 years', '60 years or older'). Variable block sizes were used in which in each block both groups were represented equally.

The main analysis was the comparison of the proportion of patients with delirium between the two arms in the Intention-to-Treat population (ITT; all registered and randomized patients) using univariate logistic regression. As sensitivity analyses the proportion of patients with delirium was also compared between the two arms in the modified ITT (m-ITT, i.e. ITT but excluding patients who were found to be ineligible after randomization) and Safety Population (i.e. all patients who underwent craniotomy). A multivariable logistic regression analysis with the stratification factors in the ITT population was also performed as sensitivity analysis, while multivariable analyses in the m-ITT and safety population should be considered as descriptive and therefore as hypothesis-generating only. All secondary outcomes were analyses in the mITT population, and should only be considered as descriptive only.

A 2-hour recording of BIS (blinded from the anaesthesiologist between operation minute 60 to 180) was split into samples of 15 minutes as time points. A 30-minute recording of HRV was split into samples of 5 minutes as time points. Subsequently, we ran a linear mixed model for BIS and HRV, as a within subject variability was suspected, with time point and



For peer review only - http://bmjopen.bmj.com/site/about/guidelines.xhtml

interaction group/time point as independent variables, as presented with fixed effects (beta/$\beta 1$) and confidence intervals (95%CI).

A two-sided p value of 0.05 or less was considered statistically significant. All statistical analysis were conducted using R (version 4.1.1).

For peer review only

## Results

From July 2020 through September 2021, patients were enrolled in the trial at the Erasmus MC until reaching sample size. A total of 309 patients were expected to be eligible after screening, of which 189 patients were registered and randomly assigned to a trial group: 95 in the music group and 94 in the standard care group (Fig. 1). A total of 5 patients (4 in the music group and 1 in the control group) were excluded after registration due to withdrawing consent (n = 1), pre-operative use of antipsychotic treatment (n=1), or no craniotomy (n = 3, one operation cancelled, one burr-hole-biopsy and one no necessity of opening the dura). The remaining 184 patients, constituting the modified intention-to-treat population, were followed up for all the secondary outcomes.

The baseline characteristics were similar in the two trial groups (ITT population), with a median age of 60 years and 44% being female (table 1). Psychiatric medical history was reported in 11%, including depression (n = 10) in most cases, pre-operative usage of delirium eliciting medication (i.e. antidepressants and sleep medication) in 15% and no dementia in our cohort. Baseline cognition (MoCA) was 24/20-27 (median/IQR), quality of life (QoL) was 70/55-80 (median/IQR) and no neurologic symptoms (in case of a KPS = 100 or MRS = 0) at admission were present in 23%. This cohort included mostly neuro-oncological patients (n = 161, 85%), with neurological deficit present in 38% and frontal localisation in 34%. Affection for music was reported with an importance of 8/7-8 (median/IQR), only 5% reported never to listen music in daily life. Surgical details showed duration of surgery of 220 minutes and emergency surgery (i.e. within 72 hours) in only 3%.

In the music group, adherence to the music intervention before, during, and directly after surgery was 96%, 100%, and 74%, respectively (supplementary table 1). The following days the adherence decreased each day, from 70% on the first morning to 47% at noon on day 3. The total listening time was a median of 130 minutes (IQR, 73-230) during the five days of admission or until discharge.

A high DOSS score (i.e. 3 or higher) was observed in 32 patients. This was caused by a neurosurgical complication, as confirmed on radiology, in three patients; two patients with infarction after a vascular procedure with hemiparesis and decreased attention. The other patient had a subdural hematoma which was evacuated in the operation room. This resulted in



For peer review only - http://bmjopen.bmj.com/site/about/guidelines.xhtml

29 patients with possible delirium by DOSS; 21 were evaluated by the psychiatrist who diagnosed delirium in 11 patients (57%, supplementary figure 2) based on the DSM-5 criteria.

*Primary outcome*

According to the DOSS, a significantly higher incidence of delirium was observed in the control vs. music group in the ITT population for the univariable (22.3 vs. 11.6%, p =0.048) and multivariable (OR/95%CI: 0.49/0.20-1.00, p =0.050) analysis. This was not observed in the mITT (p = 0.064) and the SP (p =0.064) population. The occurrence of a DSM-5 confirmed delirium, was not statistically significant between the control vs. music group in the ITT (7.4 vs 4.2%, OR = 0.55), the mITT (7.5 vs. 4.4%, OR = 0.57), and the SP (7.4 vs. 4.3%, OR = 0.58) population (table 2).

Of those patients with DSM-5-confirmed delirium (n = 11); severity of delirium (mean/SD) was 12.60/5.52, which was not different between the two arms (p =0.857). The duration of delirium (days, mean/SD) was 3.36/4.69, which was not different between the two arms (p = 0.761).

*Secondary outcome*

Available ECG data (n=87) revealed that heart rate remained constant in the music group while it decreased after 25 ($\beta_1$ = 3.00, p =0.049) and 30 ($\beta_1$ = 4.45, p=0.004) minutes in the control group (table 3 and figure 2). A significant increase on HRV was found by music at 5 minutes on RMSSD ($\beta_1$ = 51.67, p = 0.021) and a significant decrease at 5 minutes on the LF/HF ratio ($\beta_1$ -0.51, p = 0.025) with a significant increase at 20 minutes ($\beta_1$ = 0.62, p = 0.022). No significant effect was found on the other HRV parameters. Available depth of anaesthesia (n=70) data revealed a significant decrease of BIS in the music group at 30 minutes($\beta 1$ =-1.35, p = 0.007), 60 minutes ($\beta_1$ = -1.78, p=0.001), 75 minutes ($\beta_1$ -1.19, p=0.022) and 90 minutes ($\beta_1$ = -2.04, p<0.001, figure 3). A trend towards less anxiety in the music group was observed (p=0.058, figure 4). All other secondary outcomes were not different between groups.

Patient satisfaction (median/IQR) in the music group who filled in the questionnaire (n = 68), was 85/80-95, and 88% reported to want to receive music in case of future surgery.



For peer review only - http://bmjopen.bmj.com/site/about/guidelines.xhtml

## Discussion

In this study, we aimed to evaluate whether a music listening intervention could lower the onset of delirium after craniotomy. We found a significant decrease of post-operative delirium by the addition of music perioperatively using the DOSS, however this was not significant when assessed by the DSM-5 criteria. Second, music activated pre-operative heart rate variability, a marker of autonomic tone, and decreased intra-operative depth of sedation. Last, a beneficial reducing trend of music for pre-operative anxiety, but no effect on post-operative complications, length of stay and location of discharge were found. Clinical implications and limitations are discussed below.

We found a significant decrease of post-operative delirium, defined with DOSS, by the addition of perioperative music. A recent published systematic review conducted a meta-analysis on the preventive effect of music on delirium with six studies and found a relative reduction similar to ours (0.52 vs 0.48)(18) This meta-analysis included pilot studies which administered patient preferred music similar to our design, but varied from ours as the population considered surgical and non-surgical ICU patients and delirium was defined with the NEECHAM, CAM-ICU or own definitions. Our results fit well within the current literature and support the implementation of music for the prevention of delirium also within the neurosurgical population. Although a similar trend was found, significance of results was not achieved between the music and control on the onset of delirium group, when assessed by the DSM-5 criteria. Our study may have been underpowered, as suspicion of delirium after increased DOSS was not confirmed by the psychiatrist in almost half of the cases. Our sample size calculation was based on other neurosurgical studies evaluating delirium in case of increased scores on delirium screening tools. (2) Our study required a DSM-5 confirmation after an increased DOSS score. In settings such as the general medical or surgical populations, diagnostic usage of these tools is justified, as high diagnostic accuracy rates are reported.(33) However, it is unclear whether this can be adopted to our complicated patient population, as a positive screen for delirium may be due to the underlying neurological disease or its sequelae (e.g., oedema, vasospasm, seizures, rebleeding, ischemia). Future studies should validate delirium screening tools in the neurosurgical population.



For peer review only - http://bmjopen.bmj.com/site/about/guidelines.xhtml

We propose a vagal-mediated anti-inflammatory response as a candidate pathway of music on delirium, as hypotheses of delirium rely on neuro-inflammatory reactions within the brain. (3, 34) Although we did not assess inflammatory cytokines in our study, vagal nerve activation by music was supported by the increased HRV, revealed by an increased SDNN and a decreased LF/HF ratio on ECG during the pre-operative music session. The activation of HRV by music in brain-damaged patients was proven earlier and is considered a valid marker of parasympathetic nervous activation. (35, 36)Whether HRV could be used as a marker for postoperative recovery, remains to be determined. Moreover, we observed a decreasing trend in pre-operative anxiety by music, although this did not reach significance. This pre-operative parasympathetic activation and anxiety reduction may have induced a sedative-sparing effect, subsequently increasing cortical engagement and cognitive processing.(37) A dose-reduction was not expected, as the anesthesiologists administered standardized dosages of sedation while blinded for the BIS-value, but a significant deeper level of anesthesia was achieved in the music group. Literature is contradictory on the correlation of depth of sedation and anesthetic requirements as music listening is associated with decrease of depth of anesthesia (38), but no decrease of sevoflurane was achieved when pursuing constant depth of anesthesia. (39) Future neurosurgical studies should confirm whether concentration of sedation can be reduced with music in case of standardized depth of anesthesia.

We found high adherence to the music intervention before surgery. High importance of music in daily life, the number of hours listening to music in daily life and the willingness to receive music intervention in case of future surgeries was found in our cohort, which are considered important facilitators for music implementation. (40) However the adherence declined after surgery, due to pain, nausea, logistics (i.e. for MRI) or unwillingness. Absence of a near future operation prospect may have reduced the urgent necessity of music, resulting in the post-operative decline in adherence. Lack of the knowledge of the intervention is considered a barrier for implementation. Informing patients, substantiated by the results from efficacy studies such as this trial, may aid in the implementation of music in the neurosurgical population.



For peer review only - http://bmjopen.bmj.com/site/about/guidelines.xhtml

Although delirium is (most often) temporary and self-limiting, delirium independently predicts clinically relevant outcomes in neurologically damaged patients. (4) (5) Although a trend was observed, we did not find any significant positive effects on complications, length of admission or location of discharge. Future studies should assess the long-term implications of delirium defined with either DOSS and DSM-criteria after discharge in neurosurgical patients.

*Strengths and limitations*

This is the first randomized controlled trial assessing the effect of music on delirium after craniotomy and the largest assessing the effect of music against delirium. We showed that music prevents delirium when defined with the DOSS but not after DSM-5 confirmation. Our study was subject to several limitations: first, not all our patients with increased DOSS were evaluated by a psychiatrist due to logistics and we might have missed some patients with delirium. However, we feel that this did not affect our conclusions, because, with our low confirmation rate of suspected delirium, the study would still have been underpowered. Second, due to the nature of the intervention we did not blind the study, which could have influenced the outcome assessors. However, blinding could not be secured which might have led to misleading results. Third, the generalizability of the results may be affected by the single-centre design of the study.

For peer review only

## Conclusion

Our results support the efficacy of music in preventing delirium after craniotomy, as found with DOSS but not after DSM-5 confirmation. Delirium screening tools should be validated within the neurosurgical context and the long-term implications of a delirium, either defined by an increased DOSS or DSM-5, should be evaluated. This effect of music is substantiated by the effect of music on an increased pre-operative HRV and decreased intra-operative depth of anesthesia. Last, although pre-operative adherence was high, this declined after surgery which should be taken into account, when considering implementation in the neurosurgical population.

## Contributors

Each author has contributed significantly to, and is willing to take public responsibility for, one or more aspects of the study. AJPEV, CMFD, JJ, MK and PRK conceived the study idea. PRK coordinated the research protocol. PRK and MIM extracted the data. PRK, BvdH and AJPEV conducted the statistical analysis and wrote the first draft of the manuscript. JJ, MIM, CMFD, MK, SK, R-JO, MC, MJP and AJPEV critically revised the manuscript. All authors have seen and approved the final version of the manuscript being submitted. The article is the authors' original work, has not received prior publication and is not under consideration for publication elsewhere.

## Funding

This research is funded with the Erasmus MC Efficiency Grant (Grant number: 19105) by the Erasmus Medical Centre, Rotterdam, The Netherlands.

## Competing interests

None declared.

## Research ethical approval

This study involves human participants and was approved by the Medical Ethics Review Committee of the Erasmus Medical Center, Rotterdam (Erasmus MC), which declared this study not subject to the Medical Research Involving Human Subjects Act (i.e. 'Non-WMO'),



For peer review only - http://bmjopen.bmj.com/site/about/guidelines.xhtml

and approved this study (MEC-2020-0064), on the 25th of February of 2020, as such. Written informed consent was obtained for each inclusion by patient or legal representative

**Data Sharing Statement**

The raw data supporting the conclusions of this article will be made available by the authors, without undue reservation.

For peer review only

## Table 1. Baseline characteristics

| | Control (n=94) | Music (n = 95) |
|---|---|---|
| **Pre-hospital demographics** | | |
| Age (years) * | 61 (51-69) | 60 (49-69) |
| Sex (% female) | 46 (49%) | 38 (40%) |
| Medical history (n/%) | | |
| Somatic history[1] | 79 (84%) | 74 (78%) |
| Psychiatric history | 14 (15%) | 6 (6%) |
| Delirium prior admission | 2 (2%) | 5 (3%) |
| Dementia | 0 (0%) | 0 (0%) |
| Body mass index ($kg/m^2$) | 26 (24-30) | 26 (23-28) |
| Medication (n/%)[2] | 15 (26%) | 14 (15%) |
| Intoxication[3] | | |
| Abuse of alcohol (n/%) | 6 (6%) | 1 (1%) |
| Abuse of drugs (n/%) | 3 (3%) | 3 (3%) |
| **In-hospital demographics** | | |
| Pre-hospital functional status[4] | 80 (70-90) | 80 (70-90) |
| KPS (100-0) | 70 (18-80) | 70 (45-80) |
| MRS (5-1) | 1.0 (0.25-2.0) | 1.0 (1.0-2.0) |
| Quality of Life (1- 100) | 70 (60-79) | 70 (55-80) |
| Cognitive function (0 - 30)[5] | 24 (19-27) | 25 (21-27) |
| Electrolyte disturbance (n/%)[6] | 10 (11%) | 9 (10%) |
| | | |
| **Disease characteristics** | | |
| Neurologic deficit (n/%) | 35 (37%) | 36 (38%) |
| Type | | |
| Oncologic | 81 (86%) | 80 (84%) |
| Vascular | 12 (13%) | 14 (15%) |
| Other | 1 (1%) | 1 (1%) |
| Frontal disease localization (n/%) | 40 (43%) | 24 (25%) |
| **Music affection** | | |
| Music importance | 7.0 (6.8-8.0) | 8.0 (7.0-8.0) |
| Frequency listening | | |
| The whole day | 29 (31%) | 21 (22%) |
| Some hours per day | 44 (47%) | 54 (57%) |
| Some hours per week | 9 (10%) | 12 (13%) |
| Never | 6 (6%) | 3 (3%) |
| Played an instrument | 20 (21%) | 20 (21%) |
| | | |
| **Operation details[7]** | | |
| Operation duration (minutes) | 220 (160-320) | 210 (140-290) |
| Emergency operation (n/%)[8] | 2 (2%) | 4 (4%) |
| Supine position (n/%) | 81 (86%) | 80 (84%) |
| Tramrail sign tension (n/%) | 68 (72%) | 67 (7%) |

*All continuous data are presented in median/IQR. 1. Somatic history: including systematic disease (DM, hypertension) currently treated by medication and prior surgery (requiring general anesthesia on the operation room). 2. Medication known to induce delirium before admission, such as sleep medication, morphine, atropine and antidepressants 3. Reported abusive use of alcohol and/or drugs. 4. Patients' functional performance with the Karnofsky Performance Scale (ranging from 100/'No complaints' to 0/'Death') and Modified Ranking Scale (ranging from 0/'No symptoms to 5/'Death'. 5. Cognitive function assessed with the Montreal Cognitive Assessment / MoCA 6. Electrolyte disturbance (mEq/L) in case of sodium >145 or <135 or potassium <3.5 or >5. 7. Patients in the MITT population (n=184) only. 8. Operation indication within 72 hours.

For peer review only

**Table 2. Primary outcome**

| | Control (%) | Music (%) | Univariable analysis (OR/95%CI)* | P value[1] | Multivariable analysis (OR/95%CI)* | P value[2] |
|---|---|---|---|---|---|---|
| **Intention-to-treat analysis (ITT)** | | | | | | |
| Increased DOSS | 22.3% | 11.6% | 0.46/0.19;1.00 | 0.048 | 0.49/0.20;1.00 | 0.050 |
| Confirmed by DSM-5 | 7.4% | 4.2% | 0.55/0.14;1.96 | 0.342 | 0.57/0.16;2.07 | 0.39 |
| **Modified Intention-to-treat analysis (mITT)** | | | | | | |
| Increased DOSS | 22.6% | 12.1% | 0.47/0.21;1.04 | 0.060 | 0.47/0.16;2.07 | 0.064 |
| Confirmed by DSM-5 | 7.5% | 4.4% | 0.57/0.14;2.03 | 0.370 | 0.58/0.16;2.10 | 0.412 |
| **Safety Population (SP)** | | | | | | |
| Increased DOSS | 22.3% | 12.0% | 0.47/0.21;1.04 | 0.061 | 0.47/0.21;1.04 | 0.064 |
| Confirmed by DSM-5 | 7.4% | 4.3% | 0.58/0.14;2.03 | 0.370 | 0.58/0.16;2.11 | 0.414 |

*Odds Ratio/95%Confidence interval (OR/95%CI). 1. P values assessed with the Chi-squared test 2. Logistic regression analysis with groups, type disease and gender as independent variables.

For peer review only

**Table 3. Secondary outcomes**

| | Control (n = 93) | Music (n = 91) | P value |
|---|---|---|---|
| Univariable analyses | | | |
| Anxiety difference (mean/SD)[1] | 0.05/0.94 | -0.25/1.49 | 0.058 |
| Pain (mean/SD)[2] | 3.56/1.91 | 3.16/1.74 | 0.246 |
| Naproxen mg (mean/SD) | 13.6/75.4 | 2.75/26.2 | 0.103 |
| Oxycodon mg (mean/SD) | 2.03/4.35 | 1.61/3.31 | 0.828 |
| No complications (n/%) | 25 (26.9%) | 21 (23.1%) | 0.551 |
| Length of stay, days (mean/SD) | 7.43 (8.08) | 6.74 (8.26) | 0.947 |
| Discharge home (n/%) | 77 (82.8%) | 76 (83.5%) | 0.896 |

Multivariable analyses

| Heart Rate Variability/HRV ($\beta_1$/95%CI)[4] | | | | |
|---|---|---|---|---|
| Time point[3] | SDNN | RMSSD | NN50 | LF/HF |
| 5 minutes | 32.36/ -2.07;66.79 | 51.67/ 8.7-;94.64* | 16.03/ -8.09;40.14 | -0.51/ -0.96;-0.07* |
| 10 minutes | 18.93/ -18.49;56.34 | 24.93/ -12.72;62.58 | 12.48/ -7.87;32.84 | 0.51/ -0.02;1.04 |
| 15 minutes | 7.23/ -29.70;44.16 | -1.22/ -38.52;36.07 | 12.50/ -7.65;32.66 | 0.41/ -0.12;0.94 |
| 20 minutes | -4.19/ -40.93;32.54 | 6.97/ -29.88;43.83 | 9.20/ -10.86;29.27 | 0.62/ 0.10;1.13* |
| 25 minutes | -10.29/-46.93;26.34 | -9.20/ -45.99;27.59 | -1.51/ -21.54;18.51 | 0.48/-0.04;1.00 |
| 30 minutes | 11.79/-25.23;48.81 | 20.00/ -17.44;57.45 | -4.58/ -24.81;15.64 | 0.50/-0.03;1.04 |

| Time point[3] | Depth of anesthesia/BIS ($\beta_1$/95%CI)[5] | P value |
|---|---|---|
| 15 minutes | 0.63/ -3.18;4.45 | 0.742 |
| 30 minutes | -1.35/ -2.33;-0.38 | 0.007 |
| 45 minutes | -0.97/ -1.95;0.01 | 0.052 |
| 60 minutes | -1.78/ -2.78;-0.77 | 0.001 |
| 75 minutes | -1.19/ -2.21;-0.17 | 0.022 |
| 90 minutes | -2.04/ -3.08;-0.99 | <0.001 |
| 105 minutes | -0.71/ -1.79;0.38 | 0.201 |
| 120 minutes | -0.76/ -1.88;0.35 | 0.181 |

Secondary outcomes analyzed on the mITT population. 1. Anxiety differences between first and second measures with VAS-A. 2. Pain (NRS) over the first three post-operative days. 3. Time-points samples included in the linear mixed model. 4. HRV analyses; 30 minute of pre-operative ECG recordings split into 5-minute samples, all values marked with * are significant (i.e. p<0.05). 5. BIS analyses: 120 minute of intra-operative BIS registration split into 15-minute samples.

For peer review only

**Figure 1.** CONSORT Trial flowchart.

**Figure 2.** Pre-operative course of HRV. A 30-minute recording of HRV was split into samples of 5 minutes as time points and compared between groups. A significant increase on HRV was found by music at 5 minutes on RMSSD ($\beta_1$ = 51.67, p = 0.021) and decrease on the LF/HF ratio at 5 minutes ($\beta_1$ -0.51, p = 0.025) with a significant increase after 20 minutes ($\beta_1$ = 0.62, p = 0.022).

**Figure 3**. Intra-operative depth of anaesthesia. A 2-hour recording of BIS was split into samples of 15 minutes as time points and compared between groups. Available depth of anaesthesia (n=70) data revealed a significant decrease of BIS in the music group at 30 ($\beta$1 =-1.35, p = 0.007), 60 minutes ($\beta$1 = -1.78, p=0.001), 75 minutes ($\beta$1 -1.19, p=0.022) and 90 minutes ($\beta$1 = -2.04, p<0.001).

**Figure 4.** Two anxiety measurements (VAS-A) were conducted before surgery and categorized in 'No anxiety' (0 – 2), 'Medium anxiety (3-6) and 'High anxiety (7 – 10). Most patients remained in their anxiety level, although some patients showed a decrease pre-operative anxiety when receiving music, but this difference was non-significant between groups (p=0.058).

**Supplementary figure 1. Study course MUSYC trial.**

**Supplementary figure 2. Course delirium diagnosis.**

**Supplementary table 1. Adherence to music intervention during trial.**

For peer review only



For peer review only - http://bmjopen.bmj.com/site/about/guidelines.xhtml

# References

1. Oh ES, Fong TG, Hshieh TT, Inouye SK. Delirium in Older Persons: Advances in Diagnosis and Treatment. JAMA. 2017;318(12):1161-74.
2. Kappen PR, Kakar E, Dirven CMF, van der Jagt M, Klimek M, Osse RJ, et al. Delirium in neurosurgery: a systematic review and meta-analysis. Neurosurg Rev. 2021.
3. Maldonado JR. Neuropathogenesis of delirium: review of current etiologic theories and common pathways. Am J Geriatr Psychiatry. 2013;21(12):1190-222.
4. Budėnas A, Tamašauskas Š, Šliaužys A, Navickaitė I, Sidaraitė M, Pranckevičienė A, et al. Incidence and clinical significance of postoperative delirium after brain tumor surgery. Acta Neurochir. 2018;160(12):2327-37.
5. Chen H, Jiang H, Chen B, Fan L, Shi W, Jin Y, et al. The Incidence and Predictors of Postoperative Delirium After Brain Tumor Resection in Adults: A Cross-Sectional Survey. World Neurosurg. 2020;140:e129-e39.
6. Liu Y, Li XJ, Liang Y, Kang Y. Pharmacological Prevention of Postoperative Delirium: A Systematic Review and Meta-Analysis of Randomized Controlled Trials. Evid Based Complement Alternat Med. 2019;2019:9607129.
7. Vlisides P, Avidan M. Recent Advances in Preventing and Managing Postoperative Delirium. F1000Res. 2019;8.
8. Inouye SK, Westendorp RG, Saczynski JS. Delirium in elderly people. Lancet. 2014;383(9920):911-22.
9. Cerejeira J, Batista P, Nogueira V, Vaz-Serra A, Mukaetova-Ladinska EB. The stress response to surgery and postoperative delirium: evidence of hypothalamic-pituitary-adrenal axis hyperresponsiveness and decreased suppression of the GH/IGF-1 Axis. J Geriatr Psychiatry Neurol. 2013;26(3):185-94.
10. Clegg A YJ. Which medications to avoid in people at risk of delirium: a systematic review. 2011(40:23–29).
11. Fu VX, Oomens P, Sneiders D, van den Berg SAA, Feelders RA, Wijnhoven BPL, et al. The Effect of Perioperative Music on the Stress Response to Surgery: A Meta-analysis. J Surg Res. 2019;244:444-55.
12. Fu VX OP, Klimek M, Verhofstad MHJ, Jeekel J. The effect of perioperative music on medication requirement and hospital length of stay: a meta-analysis. Annals of Surgery. July 2019;272(6):961 - 72.
13. Kuhlmann AYR, de Rooij A, Kroese LF, van Dijk M, Hunink MGM, Jeekel J. Meta-analysis evaluating music interventions for anxiety and pain in surgery. Br J Surg. 2018;105(7):773-83.
14. Lee JH. The Effects of Music on Pain: A Meta-Analysis. J Music Ther. 2016;53(4):430-77.
15. Leung JM SL, Paul S. Does postoperative delirium limit the use of patient-controlled analgesia in older surgical patients? 111:625–631. 2009.
16. Schor JD, Levkoff SE, Lipsitz LA, Reilly CH, Cleary PD, Rowe JW, et al. Risk factors for delirium in hospitalized elderly. JAMA. 1992;267(6):827-31.
17. Hole J, Hirsch M, Ball E, Meads C. Music as an aid for postoperative recovery in adults: a systematic review and meta-analysis. Lancet. 2015;386(10004):1659-71.
18. Golubovic J, Neerland BE, Aune D, Baker FA. Music Interventions and Delirium in Adults: A Systematic Literature Review and Meta-Analysis. Brain Sci. 2022;12(5).
19. Kappen P, Jeekel J, Dirven CMF, Klimek M, Kushner S, Osse RJ, et al. Music to prevent deliriUm during neuroSurgerY (MUSYC) Clinical trial: a study protocol for a randomised controlled trial. BMJ Open. 2021;11(10):e048270.
20. Chan AW, Tetzlaff JM, Altman DG, Laupacis A, Gotzsche PC, Krleza-Jeric K, et al. SPIRIT 2013 statement: defining standard protocol items for clinical trials. Ann Intern Med. 2013;158(3):200-7.
21. Robinson TN, Raeburn CD, Tran ZV, Angles EM, Brenner LA, Moss M. Postoperative delirium in the elderly: risk factors and outcomes. Ann Surg. 2009;249(1):173-8.



For peer review only - http://bmjopen.bmj.com/site/about/guidelines.xhtml

22. Lee H, Ju JW, Oh SY, Kim J, Jung CW, Ryu HG. Impact of timing and duration of postoperative delirium: a retrospective observational study. Surgery. 2018;164(1):137 - 43.
23. Association AP. Diagnostic and Statistical Manual of Mental Disorders, Fourth Edition. May 2013.
24. Gavinski K, Carnahan R, Weckmann M. Validation of the delirium observation screening scale in a hospitalized older population. J Hosp Med. 2016;11(7):494-7.
25. Detroyer E, Clement PM, Baeten N, Pennemans M, Decruyenaere M, Vandenberghe J, et al. Detection of delirium in palliative care unit patients: a prospective descriptive study of the Delirium Observation Screening Scale administered by bedside nurses. Palliat Med. 2014;28(1):79-86.
26. Schuurmans MJ, Shortridge-Baggett LM, Duursma SA. The Delirium Observation Screening Scale: a screening instrument for delirium. Res Theory Nurs Pract. 2003;17(1):31-50.
27. van Velthuijsen EL, Zwakhalen SM, Warnier RM, Mulder WJ, Verhey FR, Kempen GI. Psychometric properties and feasibility of instruments for the detection of delirium in older hospitalized patients: a systematic review. Int J Geriatr Psychiatry. 2016;31(9):974-89.
28. American Psychiatric A. American Psychiatric Association DSM-5 Task Force (2013): Diagnostic and Statistical Manual of Mental Disorders: DSM-5. Washington, DC: American Psychiatric Association.
29. He Z, Cheng H, Wu H, Sun G, Yuan J. Risk factors for postoperative delirium in patients undergoing microvascular decompression. PLoS One. 2019;14(4):e0215374.
30. Zipser Z, Deuel J, Ernst J, Schubert M, von Kanel R, Bottger S. The predisposing and precipitating risk factors for delirium in neurosurgery: a prospective cohort study of 949 patients. Acta Neurochir (Wien). 2019;161(7):1307-15.
31. Janssen TL, Alberts AR, Hooft L, Mattace-Raso F, Mosk CA, van der Laan L. Prevention of postoperative delirium in elderly patients planned for elective surgery: systematic review and meta-analysis. Clin Interv Aging. 2019;14:1095-117.
32. Abraha I, Trotta F, Rimland JM, Cruz-Jentoft A, Lozano-Montoya I, Soiza RL, et al. Efficacy of Non-Pharmacological Interventions to Prevent and Treat Delirium in Older Patients: A Systematic Overview. The SENATOR project ONTOP Series. PLoS One. 2015;10(6):e0123090.
33. Inouye SK. Delirium in older persons. N Engl J Med. 2006;354(11):1157-65.
34. Tracey KJ. Reflex control of immunity. Nat Rev Immunol. 2009;9(6):418-28.
35. Florez-Perdomo WA, Garcia-Ballestas E, Moscote-Salazar LR, Konar SK, Raj S, Chouksey P, et al. Heart Rate Variability as a Predictor of Mortality in Traumatic Brain Injury: A Systematic Review and Meta-Analysis. World Neurosurg. 2021;148:80-9.
36. Mojtabavi H, Saghazadeh A, Valenti VE, Rezaei N. Can music influence cardiac autonomic system? A systematic review and narrative synthesis to evaluate its impact on heart rate variability. Complement Ther Clin Pract. 2020;39:101162.
37. Khan SH, Wang S, Harrawood A, Martinez S, Heiderscheit A, Chlan L, et al. Decreasing Delirium through Music (DDM) in critically ill, mechanically ventilated patients in the intensive care unit: study protocol for a pilot randomized controlled trial. Trials. 2017;18(1):574.
38. Ganidagli S, Cengiz M, Yanik M, Becerik C, Unal B. The effect of music on preoperative sedation and the bispectral index. Anesth Analg. 2005;101(1):103-6, table of contents.
39. Szmuk P, Aroyo N, Ezri T, Muzikant G, Weisenberg M, Sessler DI. Listening to music during anesthesia does not reduce the sevoflurane concentration needed to maintain a constant bispectral index. Anesth Analg. 2008;107(1):77-80.
40. Kakar E, Ista E, Klimek M, Jeekel J. Implementation of music in the perioperative standard care of colorectal surgery: study protocol of the IMPROVE Study. BMJ Open. 2021;11(10):e051878.



For peer review only - http://bmjopen.bmj.com/site/about/guidelines.xhtml

**Figure 1. Trial flowchart**

## CONSORT 2010 Flow Diagram

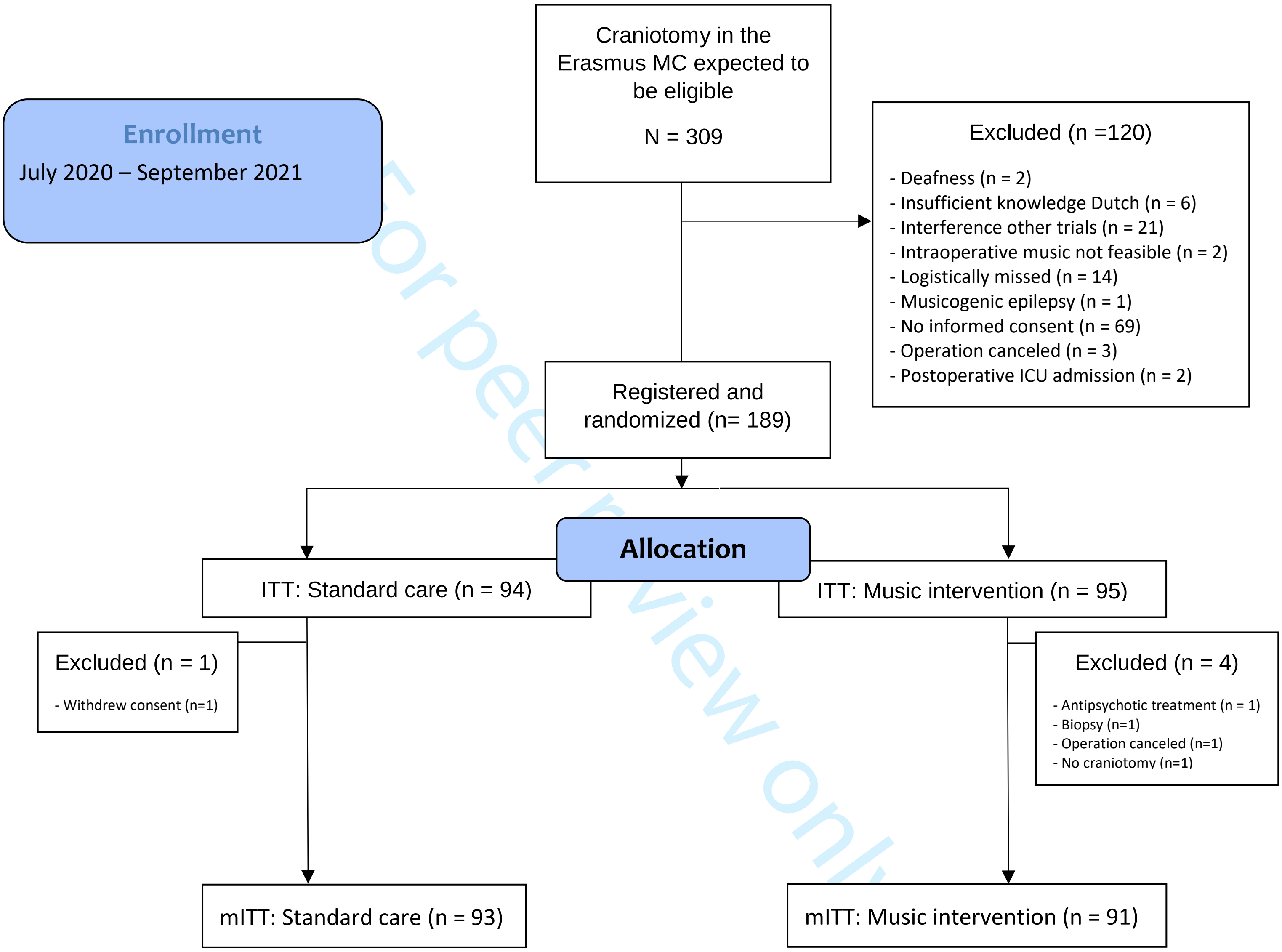


For peer review only - http://bmjopen.bmj.com/site/about/guidelines.xhtml

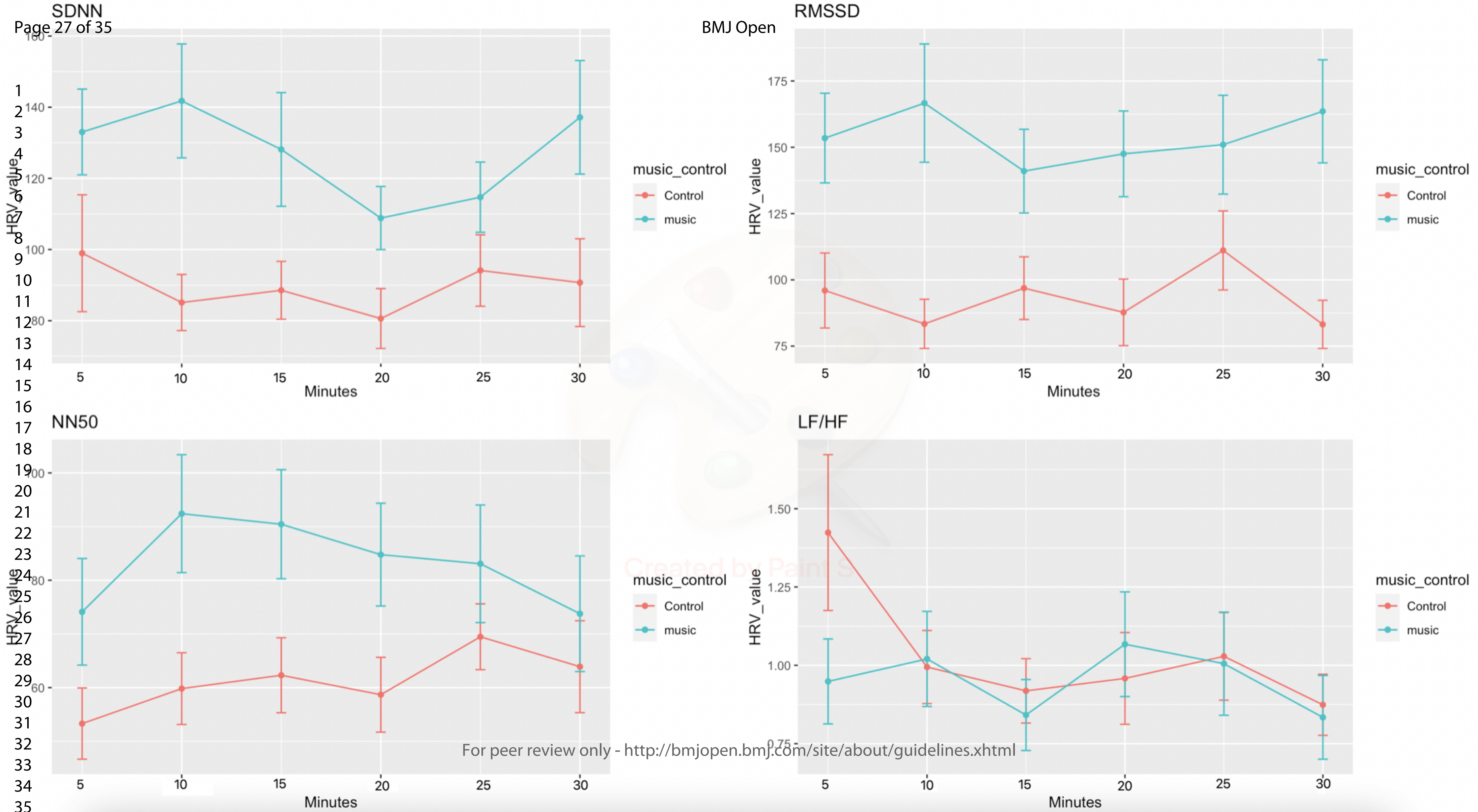

SDNN
RMSSD
NN50
LF/HF
HRV_value
Minutes
music_control
Control
music

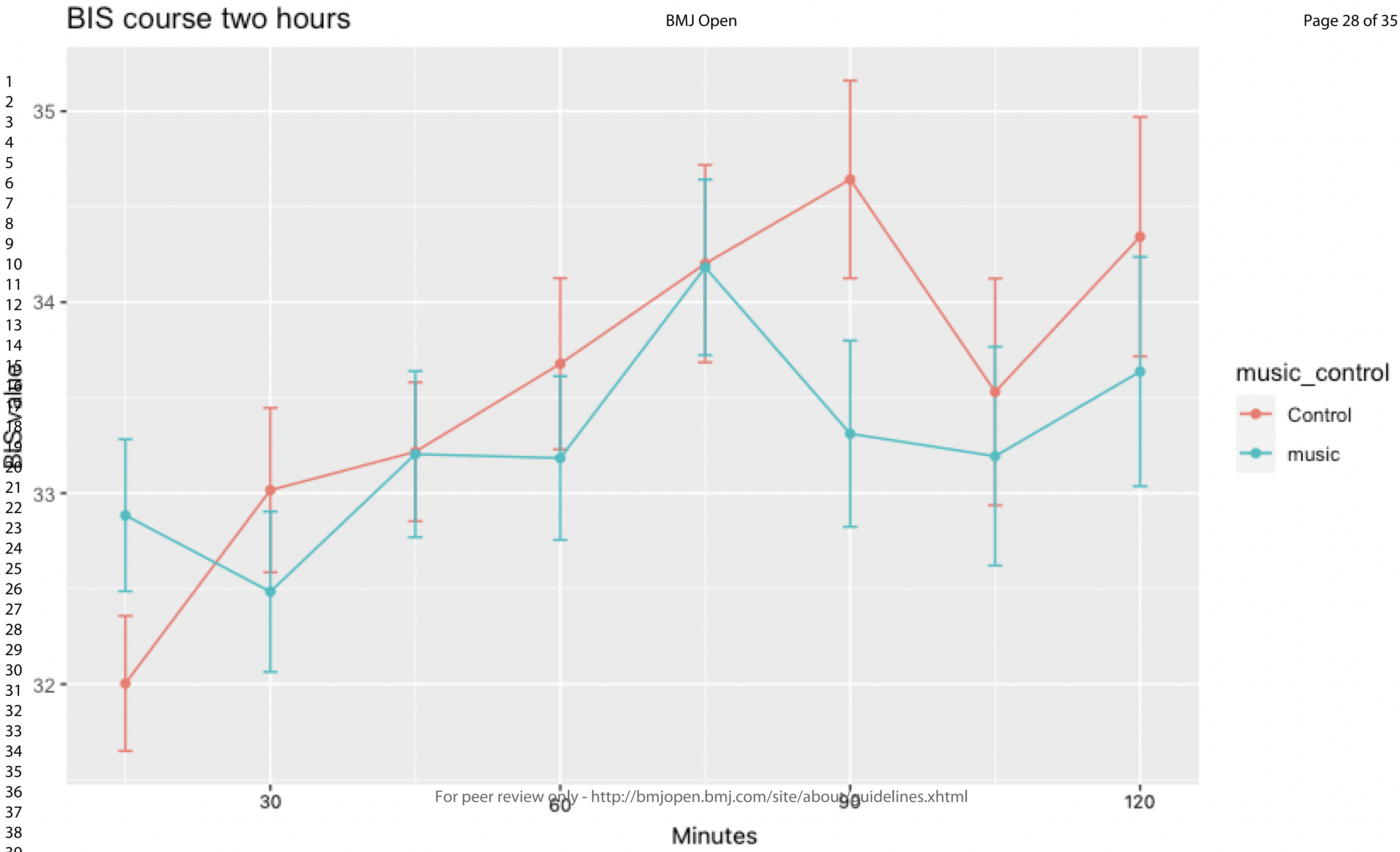


For peer review only - http://bmjopen.bmj.com/site/about/guidelines.xhtml

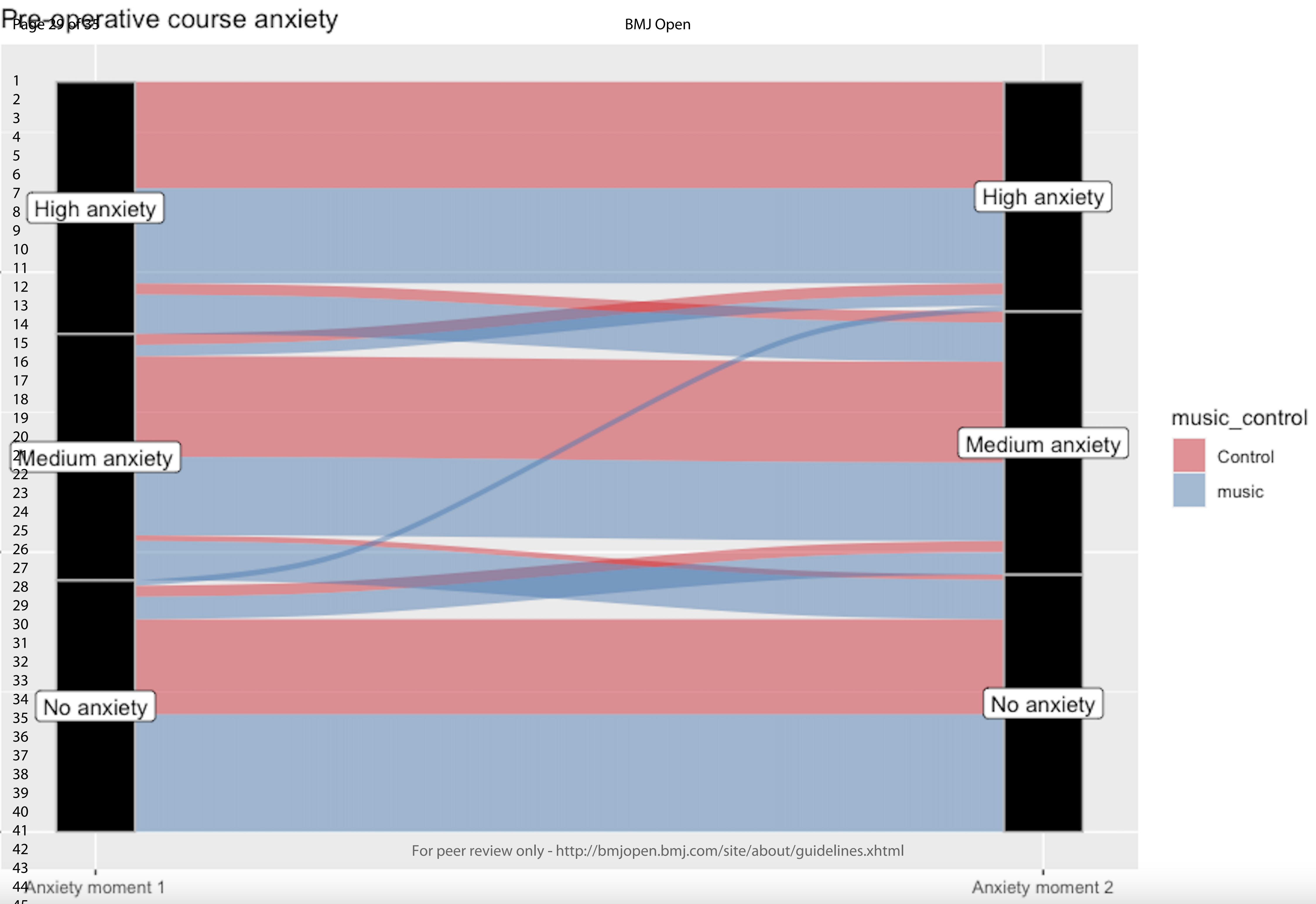
Pre-operative course anxiety
High anxiety
Medium anxiety
No anxiety
High anxiety
Medium anxiety
No anxiety
music_control
Control
music
Anxiety moment 1
Anxiety moment 2

**Supplementary figure 1. Study course MUSYC trial**

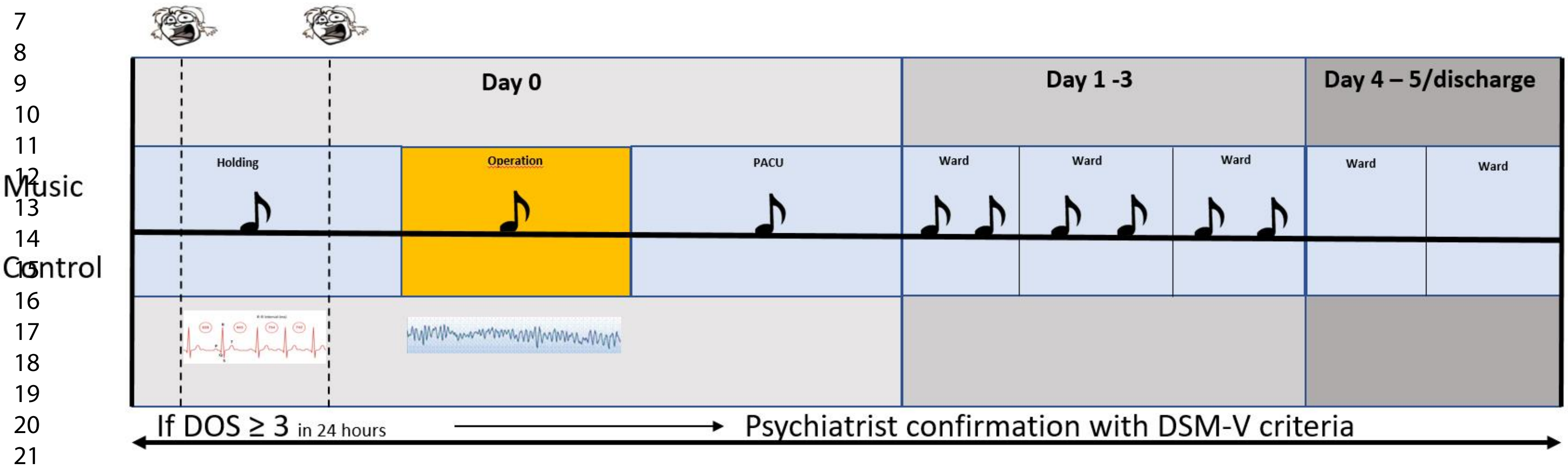


Course trial: after randomization, both groups receive ECG recording and two times anxiety measures. Intra-operative BIS registration is applied for depth of anesthesia. All participating subjects are treated according to standard care and delirium is measured until day 5 or discharge. Participants in the intervention group additionally receive music intervention 30 minutes before surgery, intra-operative during the entire operation, 30 minutes after surgery and twice per day 30minutes until post-operative day 3.

For peer review only

**Supplementary figure 2. Course delirium diagnosis**

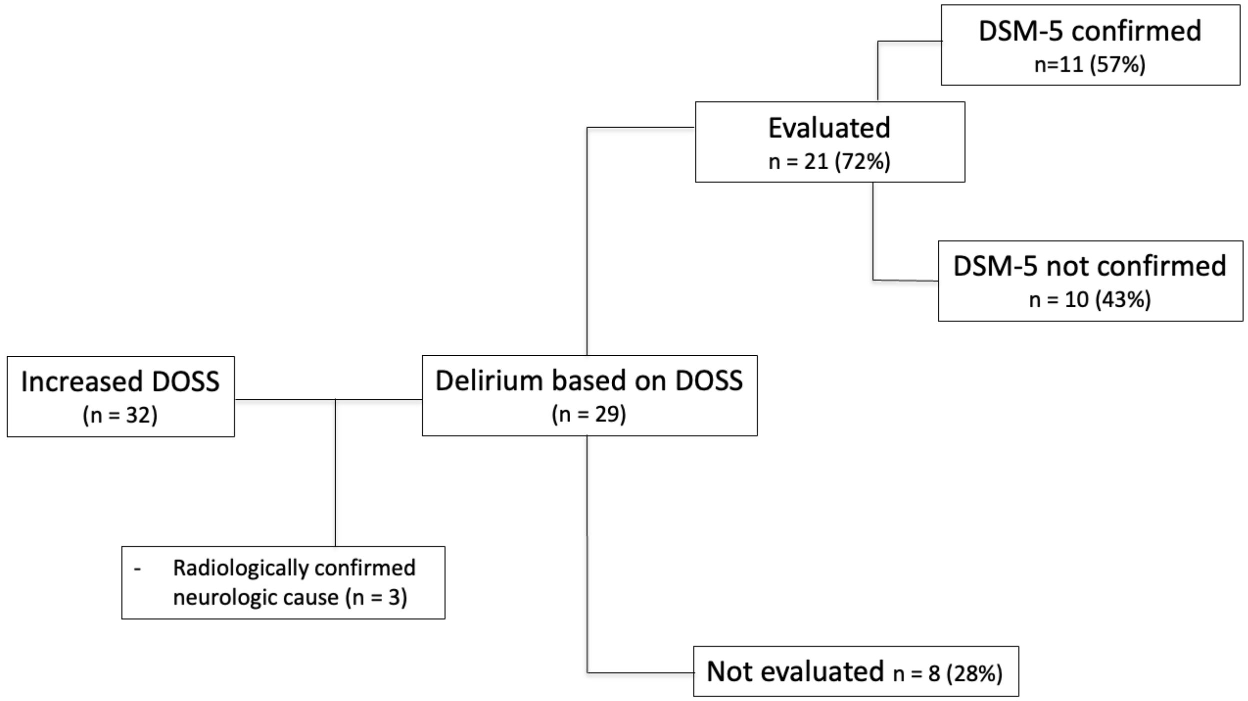


Delirium diagnosis flowchart, diagnosis of delirium required a two-step procedure. First, all participants were screened with the DOS scale. Second, in case of a daily mean score of 3 or higher (and no neurologic cause was radiologically found) a psychiatrist was consulted to assess the clinical diagnosis of delirium based on the DSM-5 criteria. In 8 patients, the psychiatrist was not consulted due to logistic issues.

**Supplementary table 1. Adherence to music intervention during trial**

| | Day 0 | | | Day 1 | | Day 2 | | Day 3 | |
|---|---|---|---|---|---|---|---|---|---|
| **Moment[1]** | **Pre** | **Per** | **Post** | **Morning** | **Noon** | **Morning** | **Noon** | **Morning** | **Noon** |
| **N[2]** | 91 | 91 | 91 | 91 | 91 | 88 | 88 | 70 | 70 |
| | | | | | | | | | |
| **Music** | | | | | | | | | |
| **No** | 3 | 0 | 21 | 25 | 26 | 26 | 21 | 19 | 20 |
| **Yes** | 79 | 91 | 61 | 59 | 45 | 44 | 39 | 24 | 18 |
| **Unknown[3]** | 9 | 0 | 9 | 7 | 20 | 18 | 28 | 27 | 32 |
| **Adherence[4]** | 96% | 100% | 74% | 70% | 63% | 63% | 65% | 56% | 47% |

Patients in the mITT population allocated to the music group. 1. Session of 30 minutes in the morning or afternoon. 2. Amount of patients in the musical group not discharged from the ward. 3. Unregistered music session. 4. Adherence calculated from the registered patients (i.e. 'Yes'/'No' + 'Yes')

For peer review only

## 2017 CONSORT checklist of information to include when reporting a randomized trial assessing nonpharmacologic treatments (NPTs)*. Modifications of the extension appear in italics and blue.

| Section/Topic Item | Checklist item no. | CONSORT item | Extension for NPT trials | Page |
|---|---|---|---|---|
| **Title and abstract** | | | | |
| | 1a | Identification as a randomized trial in the title | | 1 |
| | 1b | Structured summary of trial design, methods, results, and conclusions (for specific guidance see CONSORT for abstracts) | *Refer to CONSORT extension for abstracts for NPT trials* | *3* |
| **Introduction** | | | | |
| Background and objectives | 2a | Scientific background and explanation of rationale | | 5, 6 |
| | 2b | Specific objectives or hypotheses | | 5, 6 |
| **Methods** | | | | |
| Trial design | 3a | Description of trial design (such as parallel, factorial) including allocation ratio | When applicable, how care providers were allocated to each trial group | 7:11 |
| | 3b | Important changes to methods after trial commencement (such as eligibility criteria), with reasons | | 7, 9 |
| Participants | 4a | Eligibility criteria for participants | When applicable, eligibility criteria for centers and for *care providers* | 7 |
| | 4b | Settings and locations where the data were collected | | 7 |
| Interventions *†* | 5 | The interventions for each group with sufficient details to allow replication, including how and when they were actually administered | Precise details of both the experimental treatment and comparator | 8 |
| | 5a | | Description of the different components of the interventions and, when applicable, description of the procedure for tailoring the interventions to individual participants. | 8, supplementary figures |
| | 5b | | Details *of whether and* how the interventions were standardized. | 8, supplementary figures |
| | 5c. | | Details *of whether and* how adherence of care providers to the protocol was assessed or enhanced | 8, supplementary figures |

*Cite as:* *Boutron I, Altman DG, Moher D, Schulz KF, Ravaud P. CONSORT Statement for Randomized Trials of Nonpharmacologic Treatments: A 2017 Update and a CONSORT Extension for Nonpharmacologic Trial Abstracts. Annals of Internal Medicine. 2017 Jul 4;167(1):40–7.*

For peer review only - http://bmjopen.bmj.com/site/about/guidelines.xhtml

| Section/Topic Item | Checklist item no. | CONSORT item | Extension for NPT trials | Page |
|---|---|---|---|---|
| | 5d | | *Details of whether and how adherence of participants to interventions was assessed or enhanced* | *8* |
| Outcomes | 6a | Completely defined pre-specified primary and secondary outcome measures, including how and when they were assessed | | **8, 9** |
| | 6b | Any changes to trial outcomes after the trial commenced, with reasons | | **7, 9** |
| Sample size | 7a | How sample size was determined | When applicable, details of whether and how the clustering by care providers or centers was addressed | 10 |
| | 7b | When applicable, explanation of any interim analyses and stopping guidelines | | NA |
| Randomization: | | | | 10 |
| - Sequence generation | 8a | Method used to generate the random allocation sequence | | 10 |
| | 8b | Type of randomization; details of any restriction (such as blocking and block size) | | 10 |
| - Allocation concealment mechanism | 9 | Mechanism used to implement the random allocation sequence (such as sequentially numbered containers), describing any steps taken to conceal the sequence until interventions were assigned | | 10 |
| - Implementation | 10 | Who generated the random allocation sequence, who enrolled participants, and who assigned participants to interventions | | 10 |
| Blinding | 11a | If done, who was blinded after assignment to interventions (for example, participants, care providers, those assessing outcomes) and how | If done, who was blinded after assignment to interventions (e.g., participants, care providers, *those administering co-interventions,* those assessing outcomes) and how | 8 |
| | 11b | If relevant, description of the similarity of interventions | If blinded, method of blinding and description of the similarity of interventions | NA |
| | 11c | | *If blinding was not possible, description of any attempts to limit bias* | *8* |

***Cite as:*** *Boutron I, Altman DG, Moher D, Schulz KF, Ravaud P. CONSORT Statement for Randomized Trials of Nonpharmacologic Treatments: A 2017 Update and a CONSORT Extension for Nonpharmacologic Trial Abstracts. Annals of Internal Medicine. 2017 Jul 4;167(1):40–7.*

| Section/Topic Item | Checklist item no. | CONSORT item | Extension for NPT trials | Page |
|---|---|---|---|---|
| Statistical methods | 12a | Statistical methods used to compare groups for primary and secondary outcomes | When applicable, details of whether and how the clustering by care providers or centers was addressed | 10, 11 |
| | 12b | Methods for additional analyses, such as subgroup analyses and adjusted analyses | | 10, 11 |
| **Results** | | | | |
| Participant flow (a diagram is strongly recommended) | 13a | For each group, the numbers of participants who were randomly assigned, received intended treatment, and were analyzed for the primary outcome | The number of care providers or centers performing the intervention in each group and the number of patients treated by each care provider or in each center | 12, figure 1 |
| | 13b | For each group, losses and exclusions after randomization, together with reasons | | 12, figure 1 |
| | 13c | | *For each group, the delay between randomization and the initiation of the intervention* | *Supplementary figures* |
| | new | | Details of the experimental treatment and comparator as they were implemented | *Supplementary figures* |
| Recruitment | 14a | Dates defining the periods of recruitment and follow-up | | **12, figure 1** |
| | 14b | Why the trial ended or was stopped | | **12** |
| Baseline data | 15 | A table showing baseline demographic and clinical characteristics for each group | When applicable, a description of care providers (case volume, qualification, expertise, etc.) and centers (volume) in each group. | Table 1 |
| Numbers analyzed | 16 | For each group, number of participants (denominator) included in each analysis and whether the analysis was by original assigned groups | | 10, 11, Table 1 |
| Outcomes and estimation | 17a | For each primary and secondary outcome, results for each group, and the estimated effect size and its precision (such as 95% confidence interval) | | 12, 13, table 2, 3 and figure 2, 3 and 4 |
| | 17b | For binary outcomes, presentation of both absolute and relative effect sizes is recommended | | 12, 13, table 2, 3 and figure 2, 3 and 4 |
| Ancillary analyses | 18 | Results of any other analyses performed, including subgroup analyses and adjusted analyses, distinguishing pre-specified from exploratory | | 12, 13, table 2, 3 and figure 2, 3 and 4 |

***Cite as:*** *Boutron I, Altman DG, Moher D, Schulz KF, Ravaud P. CONSORT Statement for Randomized Trials of Nonpharmacologic Treatments: A 2017 Update and a CONSORT Extension for Nonpharmacologic Trial Abstracts. Annals of Internal Medicine. 2017 Jul 4;167(1):40–7.*

| Section/Topic Item | Checklist item no. | CONSORT item | Extension for NPT trials | Page |
|---|---|---|---|---|
| Harms | 19 | All important harms or unintended effects in each group (for specific guidance see CONSORT for harms) | | NA |
| **Discussion** | | | | |
| Limitations | 20 | Trial limitations, addressing sources of potential bias, imprecision, and, if relevant, multiplicity of analyses | In addition, take into account the choice of the comparator, lack of or partial blinding, and unequal expertise of care providers or centers in each group | 16 |
| Generalizability | 21 | Generalizability (external validity, applicability) of the trial findings | Generalizability (external validity) of the trial findings according to the intervention, comparators, patients, and care providers and centers involved in the trial | 14-16 |
| Interpretation | 22 | Interpretation consistent with results, balancing benefits and harms, and considering other relevant evidence | | 14-16 |
| **Other information** | | | | |
| Registration | 23 | Registration number and name of trial registry | | 7 |
| Protocol | 24 | Where the full trial protocol can be accessed, if available | | 7 |
| Funding | 25 | Sources of funding and other support (such as supply of drugs), role of funders | | 17 |

**Additions or modifications to the 2010 CONSORT checklist. CONSORT = Consolidated Standards of Reporting Trials*
*†The items 5, 5a, 5b, 5c, 5d are consistent with the Template for Intervention Description and Replication (TIDieR) checklist*

***Cite as:*** *Boutron I, Altman DG, Moher D, Schulz KF, Ravaud P. CONSORT Statement for Randomized Trials of Nonpharmacologic Treatments: A 2017 Update and a CONSORT Extension for Nonpharmacologic Trial Abstracts. Annals of Internal Medicine. 2017 Jul 4;167(1):40–7.*

For peer review only - http://bmjopen.bmj.com/site/about/guidelines.xhtml

BMJ Open

# Music to prevent delirium during neurosurgery (MUSYC): a single-centre prospective randomised controlled trial.

| | |
|---|---|
| Journal: | *BMJ Open* |
| Manuscript ID | bmjopen-2022-069957.R1 |
| Article Type: | Original research |
| Date Submitted by the Author: | 31-Mar-2023 |
| Complete List of Authors: | Kappen, Pablo; Erasmus Medical Center, Department of Neurosurgery; Erasmus Medical Center, Department of Neuroscience<br>Mos, M.I.; Erasmus Medical Center, Department of Neurosurgery; Erasmus Medical Center, Department of Neuroscience<br>Jeekel, Johannes; Erasmus Medical Center, Department of Neuroscience<br>Dirven, Clemens; Erasmus Medical Center, Department of Neurosurgery<br>Kushner, Steven; Erasmus Medical Center, Department of Psychiatry<br>Osse, Robert-Jan; Erasmus Medical Center, Department of Psychiatry<br>Coesmans, Michiel; Erasmus Medical Center, Department of Psychiatry<br>Poley, Marten; Erasmus Medical Center, Institute for Medical Technology Assessment (iMTA); Erasmus Medical Center, Department of Paediatric Surgery and Intensive Care<br>van Schie, Mathijs; Erasmus Medical Center, Department of Cardiology<br>van der Holt, Bronno; Erasmus Medical Center, Department of Haematology<br>Klimek, M; Erasmus Medical Center, Anesthesiology<br>Vincent, Arnaud; Erasmus Medical Center, Department of Neurosurgery |
| <b>Primary Subject Heading</b>: | Neurology |
| Secondary Subject Heading: | Anaesthesia, Surgery, Patient-centred medicine, Nursing |
| Keywords: | Neurosurgery < SURGERY, Delirium & cognitive disorders < PSYCHIATRY, COMPLEMENTARY MEDICINE, Clinical trials < THERAPEUTICS, PREVENTIVE MEDICINE |
| | |

SCHOLARONE™
Manuscripts

*I, the Submitting Author has the right to grant and does grant on behalf of all authors of the Work (as defined in the below author licence), an exclusive licence and/or a non-exclusive licence for contributions from authors who are: i) UK Crown employees; ii) where BMJ has agreed a CC-BY licence shall apply, and/or iii) in accordance with the terms applicable for US Federal Government officers or employees acting as part of their official duties; on a worldwide, perpetual, irrevocable, royalty-free basis to BMJ Publishing Group Ltd ("BMJ") its licensees and where the relevant Journal is co-owned by BMJ to the co-owners of the Journal, to publish the Work in this journal and any other BMJ products and to exploit all rights, as set out in our licence.*

*The Submitting Author accepts and understands that any supply made under these terms is made by BMJ to the Submitting Author unless you are acting as an employee on behalf of your employer or a postgraduate student of an affiliated institution which is paying any applicable article publishing charge ("APC") for Open Access articles. Where the Submitting Author wishes to make the Work available on an Open Access basis (and intends to pay the relevant APC), the terms of reuse of such Open Access shall be governed by a Creative Commons licence – details of these licences and which Creative Commons licence will apply to this Work are set out in our licence referred to above.*

*Other than as permitted in any relevant BMJ Author's Self Archiving Policies, I confirm this Work has not been accepted for publication elsewhere, is not being considered for publication elsewhere and does not duplicate material already published. I confirm all authors consent to publication of this Work and authorise the granting of this licence.*

For peer review only

# Music to prevent delirium during neurosurgery (MUSYC): a single-centre prospective randomised controlled trial.

P.R. Kappen✉, [1,2], M.I. Mos[1,2], J. Jeekel[2], C.M.F. Dirven[1], S.A. Kushner[3], R.J. Osse[3], M. Coesmans[3], M.J. Poley[4,5], M.S. van Schie[6], B. van der Holt[7], M. Klimek[8], A.J.P.E. Vincent[1]

**Affiliations.**

✉ Corresponding Author; Mr. Pablo Kappen, address: Doctor Molewaterplein 40, 3015 GD Rotterdam, the Netherlands, Room NA-2124, phone: +31(0)10 704 35 41, email: p.kappen@erasmusmc.nl

1. Department of Neurosurgery, Erasmus MC University Medical Center, Rotterdam, the Netherlands
2. Department of Neuroscience, Erasmus MC University Medical Center, Rotterdam, the Netherlands
3. Department of Psychiatry, Erasmus MC University Medical Center, Rotterdam, the Netherlands
4. Institute for Medical Technology Assessment (iMTA), Erasmus MC University Medical Center, Rotterdam, the Netherlands
5. Department of Paediatric Surgery and Intensive Care, Sophia Children's Hospital, Erasmus MC University Medical Center, Rotterdam, the Netherlands
6. Department of Cardiology, Erasmus MC University Medical Center, Rotterdam, the Netherlands
7. Department of Haematology, Erasmus Medical Center Cancer Institute, Rotterdam, the Netherlands
8. Department of Anaesthesiology, Erasmus Medical Center, Rotterdam, the Netherlands

**Abstract word count:** 245

**Manuscript word count:** 3717

**Reference count:** 41

**Number of figures:** 4

**Number of tables:** 3



For peer review only - http://bmjopen.bmj.com/site/about/guidelines.xhtml

**Bullet points**

- Music reduced the onset of delirium after craniotomy, as confirmed with DOSS but not with DSM-5
- Music activated pre-operative heart rate variability, a marker of autonomic tone
- A beneficial reducing trend of music for pre-operative anxiety, but no effect on depth of anaesthesia, post-operative complications, length of stay and location of discharge were found

For peer review only



For peer review only - http://bmjopen.bmj.com/site/about/guidelines.xhtml

## Abstract

### Objectives

Delirium is a serious complication following neurosurgical procedures, as high incidence rates and associations with increased length of hospital stay, number of re-operations and mortality are reported. We hypothesize that the beneficial effect of music on a combination of delirium-eliciting factors might reduce delirium incidence following neurosurgery and subsequently improve clinical outcomes.

### Methods

This single-centre prospective randomized controlled trial in adults compared the effects of music administered before, during and after craniotomy with standard of clinical care. We enrolled 189 patients (music = 95, control = 94) from July 2020 through September 2021. The primary endpoint delirium was assessed by the delirium observation screening scale (DOSS) and confirmed by a psychiatrist according to DSM-5 criteria. A variety of secondary outcomes were assessed to substantiate the effects of music on delirium and its clinical implications.

### Results

Delirium, as assessed by the DOSS, was less common in the music (n = 11, 11.6%) than in the control group (n =21, 22.3%, OR:0.49, p=0.048). However, after DSM-5 confirmation, differences in delirium were not significant (4.2% vs. 7.4%, OR:0.47, p=0.342). Moreover, music increased the heart rate variability (RMSSD, p=0.012). All other secondary outcomes were not different between groups.

### Conclusion

Our results support the efficacy of music in reducing the incidence of delirium after craniotomy, as found with DOSS but not after DSM-5 confirmation, substantiated by the effect of music on pre-operative autonomic tone. Delirium screening tools should be validated and the long-term implications should be evaluated after craniotomy.




For peer review only - http://bmjopen.bmj.com/site/about/guidelines.xhtml

## Introduction

Delirium is defined in the latest Diagnostic and Statistical Manual of Mental Disorders (DSM-5) as "an acute disturbance in attention and cognition which is not better explained by another neurocognitive disorder such as for example dementia". To increase the recognition of delirium during hospital stay, a variety of delirium diagnostic screening tools have been developed, which can also be assessed by other healthcare workers. Delirium in neurosurgical patients has been reported in 4 to 44% of cases, with a large variation in definition and assessment methods. (1) The high incidence in this population is probably caused by the underlying massive neuro-inflammation which is usually induced during intracranial procedures. (2) Delirium, also in neurosurgical literature, is often multifactorial in aetiology and can be influenced by a number of predisposing (e.g. older age, cognitive impairment, multiple comorbidities) and precipitating factors (e.g. infections, operations, drugs).(3-8) The clinical relevance of delirium in neurosurgery remains difficult to assess objectively, as criteria for delirium overlap with symptoms from the primary neurologic injury. However, delirium independently predicted clinical outcomes in neurosurgical and neurocritically ill patients such as worse functional outcome (9), length of stay, costs and death. (10) These complications justify the search for preventive therapies for post-operative delirium in neurosurgical patients.

Although promising preventive approaches are emerging, pharmacological treatments have inconsistent results and are accompanied with side effects. (11, 12) Non-pharmacological multi-component approaches for primary prevention, such as reorientation, early mobilization, therapeutic activities, hydration, nutrition and sleep strategies have been shown to be effective and cost-reducing in other patient groups. However, these approaches can be labour intensive, and include the use of volunteers, or non-licensed professionals to enhance feasibility. (13)

Recorded music is an easy applicable intervention which neatly fits throughout the entire perioperative process and has been shown to be effective in the surgical population in reducing a combination of delirium-eliciting factors such as pre-operative anxiety, post-operative pain, stress response and opioid/sedation requirement.(14-21) A recent meta-



For peer review only - http://bmjopen.bmj.com/site/about/guidelines.xhtml

analysis, evaluating six randomised pilot studies, found music potentially being effective in preventing postoperative delirium in postsurgical patients. However, these studies did not include neurosurgical patients. (22)

We therefore designed a randomized controlled trial to assess the effect of music in the prevention of post-operative delirium in neurosurgical patients.

For peer review only



For peer review only - http://bmjopen.bmj.com/site/about/guidelines.xhtml

## Methods

*Patient and public involvement*

Patients were involved in the composition of the music playlists, as these were based on their music preference, the role music plays in their life (ie, whether they are musician/ just listen to music) and the importance of music. The results of our trial were disseminated to the participating patients through a letter after publication.

*Study design*

The Music to prevent deliriUm during neuroSurgerY Clinical (MUSYC) trial was a single centre, prospective randomized controlled trial conducted at the Erasmus MC Rotterdam, the Netherlands. The trial compared effects of music administered before, during and after craniotomy with standard of clinical care. The Medical Ethics Review Committee of the Erasmus Medical Center, Rotterdam (Erasmus MC), declared this study not subject to the Medical Research Involving Human Subjects Act (i.e. 'Non-WMO'), and approved this study (MEC-2020-0064), on the 25$^{th}$ of February of 2020, as such.

The trial protocol was designed by neurosurgeons, psychiatrists, anaesthesiologists and neuroscientists and followed the Standard Protocol Items: Recommendations for Interventional Trials (SPIRIT) guidelines and the Consolidated Standards of Reporting Trials (CONSORT) guidelines for non-pharmacological treatments (see checklist in online supplementary material). The trial was registered (trialregister.nl: NL8503 and ClinicalTrials.gov: NCT04649450) and details of the protocol have been published previously. (23, 24)

We expected an incidence of delirium in our control group of 30%, which was based on the incidence of 24.2–32.4% documented in neurosurgical literature using the same screening tool (i.e. DOSS).(5, 7) When designing the trial, the expected effect could not be based on previous literature as no pooled effect of music on delirium was reported. Other non-pharmacological interventions mentioned a relative reduction of 36%–77% and we therefore considered the intervention clinically relevant if a relative reduction of 60%, corresponding to an absolute reduction of 18%, was achieved.(25, 26) Assuming a loss to follow-up of 5%, we



For peer review only - http://bmjopen.bmj.com/site/about/guidelines.xhtml

estimated that a target sample size of 189 patients would provide the trial with a power of 80%.

From July 2020 through September 2021: 189 patients were registered and randomly assigned to a trial group: 95 in the music group and 94 in the standard care group (Fig. 1). Randomization was done in a 1:1 ratio, by a secured online software program (ALEA; FormsVision BV, Abcoude, The Netherlands) and stratified per type of disease characteristic (i.e. 'neuro-oncology', 'neurovascular', 'traumatic brain injury', 'infectious') and age (i.e. 'younger than 60 years', '60 years or older'). Variable block sizes were used in which in each block both groups were represented equally.

*Patients*

Adult patients (i.e. age 18 years or more) undergoing craniotomy (i.e. opening the dura requiring bone flap removal) at the Erasmus MC with sufficient knowledge of the Dutch language were eligible for study participation. Exclusion criteria were: impaired awareness before surgery (i.e. motoric less than 6 in the Glasgow Coma Scale), planned post-operative intensive care unit (ICU) admission, suspected delirium (defined as fluctuating awareness) at baseline, antipsychotic treatment, undergoing surgery impeding supply of music (i.e. awake craniotomy or vestibular schwannoma surgery), bilateral hearing impairment and participation in other clinical trials interfering with results. During inclusion, one participant reported that music induced epileptic seizures (known as musicogenic epilepsy): this patient was excluded (and the exclusion criteria were adopted accordingly), as it was considered unethical to expose such a patient to music. Eligible patients were approached and written informed consent by patient or legal representative was obtained.

*Intervention*

All participating subjects were treated according to standard of care. Method of music intervention administration (i.e. type, frequency and duration) was applied based on previous studies.(17, 18) Participants in the intervention group (i.e. music group) received an over-ear headphone and a tablet with access to a platform with different pre-selected music playlists (i.e. jazz, blues, classic, electronic, pop, 60s, 70s, 80s etc), in which the music selection could be extended based on patients' wishes. These patients received the first 30 minutes of music at the pre-operative holding area the day of operation (day 0) while awaiting surgery (see



For peer review only - http://bmjopen.bmj.com/site/about/guidelines.xhtml

supplementary figure 1 for 'Study Course MUSYC trial') which was stopped before reaching the operation room. In the operating room, in-ear earphones after intubation, which were compatible with the Mayfield clamp and site of operation, were inserted and music was continued until just before detubation. After surgery, during recovery at the post-operative care unit (PACU), another 30 minutes of recorded music through over-ear headphones was administered. Finally, participants received 30 minutes of recorded music twice a day until post-operative day 3. Patients in the control group were asked to refrain from music listening, however this was not strictly controlled as this would influence the standard of clinical care too much. Nurses were instructed to monitor for music listening behaviour with a diary which was placed next to the music equipment. Periodically (approximately every six months) a training was given for all nurses on the ward to explain how music had to be administered and monitored.

*Outcome measures*

The primary outcome measure was presence or absence of postoperative delirium within the first five postoperative days. (27) The diagnosis of delirium required a two-step procedure; first, all participating patients were daily screened by the treating nurse using the Delirium Observation Screening scale (DOSS), a validated 13-item delirium screening tool with higher scores indicating a higher probability of delirium. Use of the DOSS was already current practice at our department and was administered by the nurse during each shift (three- 8-hour shifts per day).(28-31) Second, in case of a daily mean score of 3 or higher, which was radiologically not substantiated by a neurosurgical complication, a psychiatrist was consulted to assess the clinical diagnosis of delirium based on the DSM-5 criteria.(32) DSM-5 criteria assessment was conducted once in case of an increased mean DOS score of 3 or above. This was not standardized for a certain moment of the day, but depended on the timing of the increased mean DOSS score and the logistic of the consulting psychiatrist that specific day. We chose not to blind the assessors from the intervention, as this could not be secured which might have led to misleading results.

Secondary outcomes were assessed to substantiate the effects of music on delirium and its clinical implications. Pre-operative secondary outcomes (during the 30-minute pre-operative holding stay) included anxiety (measured with the Visual Analogue Scale-anxiety/VAS-A) and heart rate variability (HRV), a marker of the autonomic tone reflecting parasympathetic



For peer review only - http://bmjopen.bmj.com/site/about/guidelines.xhtml

nervous activity, measured with a 30 minutes electrocardiography (ECG) recording. The following HRV parameters were analysed: standard deviation of normal sinus beats (SDNN), root mean square of successive differences between normal heartbeats (RMSSD), the number of adjacent NN intervals that differ from each other by more than 50 ms (NN50) and the ratio of low frequency to high frequency power (HF/LF). Intra-operative secondary outcomes included depth of anaesthesia with Bispectral Index (BIS, Aspect ™ version 3.22) with standardized sedation dosages (propofol and remifentanil). BIS was measured from the non-operated side, if feasible with site of resection, and the anaesthesiologist was blinded from the intra-operative BIS values, which was considered ethical as this form of monitoring is not standard of clinical care during intracranial procedures. Post-operative secondary outcomes (measured during the entire post-operative stay) were delirium severity (using the Delirium Rating Scale-revised-98/DRS-R-98) and delirium duration (onset until first day DOSS score <3), pain (Numeric Rating Scale/NRS pain and dosages analgesic), post-operative complications, length of stay and location of discharge. Finally, patients' satisfaction of receiving music was assessed with a 100- point visual analogue scale (administered at the outpatient clinic 6 weeks after discharge).

Baseline characteristics were extracted at baseline from questionnaires or the electronic patient file consisting of age, gender, medical history, daily function (Karnofsky Performance Scale/KPS or Modified Rankin scale/MRS), quality of life (100-likert scale, EQ-5D and EORTC QLQ – BN-20), cognitive function (Montreal Cognitive assessment / MoCA), disease characteristics (i.e. neurologic deficit, type and side of intracranial pathology) and operation details (i.e. emergency grade, duration of surgery).

*Statistical analysis*

The main analysis was the comparison of the proportion of patients with delirium between the two arms in the Intention-to-Treat population (ITT; all registered and randomized patients) using univariate logistic regression. As sensitivity analyses the proportion of patients with delirium was also compared between the two arms in the modified ITT (m-ITT, i.e. ITT but excluding patients who were found to be ineligible after randomization) and Safety Population (i.e. all patients who underwent craniotomy). A multivariable logistic regression analysis with the stratification factors in the ITT population was also performed as sensitivity analysis, while multivariable analyses in the m-ITT and safety population should

For peer review only

be considered as descriptive and therefore as hypothesis-generating only. All secondary outcomes were analyses in the mITT population, and should only be considered as descriptive only.

A 2-hour recording of BIS (blinded from the anaesthesiologist between operation minute 60 to 180) was split into samples of 15 minutes as time points. A 30-minute recording of HRV was split into samples of 5 minutes as time points. Subsequently, we ran a linear mixed model with unstructured covariance for BIS and HRV, as a within subject variability was suspected, with time point and interaction group/time point as independent variables, as presented with fixed effects (beta/$\beta$1) and confidence intervals (95%CI). Moreover, a sensitivity analyses was conducted with possible additional confounding for depth of anaesthesia (BIS level) including age, comorbidity, type of disease, American Society of Anesthesiologists (ASA) classification and steroid use. The residual plots were visually observed and a log transformation was applied in case of heteroscedasticity.

A two-sided p value of 0.05 or less was considered statistically significant. All statistical analysis were conducted using R (version 4.1.1).



For peer review only - http://bmjopen.bmj.com/site/about/guidelines.xhtml

## Results

A total of 309 patients were expected to be eligible after screening, of which 189 patients were registered and randomly assigned to the music (n=95) or control group (n=94). Five patients (4 in the music group and 1 in the control group) were excluded after registration due to withdrawing consent (n = 1), pre-operative use of antipsychotic treatment (n=1), or no craniotomy (n = 3, one operation cancelled, one burr-hole-biopsy and one no necessity of opening the dura). The remaining 184 patients, constituting the modified intention-to-treat population, were followed up for all the secondary outcomes.

The baseline characteristics were similar in the two trial groups (ITT population), with a median age of 60 years and 44% being female (table 1). Psychiatric medical history was reported in 11%, including depression (n = 10) in most cases, pre-operative usage of possible delirium eliciting medication (i.e. antidepressants and sleep medication) in 15% and no dementia in our cohort. Baseline cognition (MoCA) was 24/20-27 (median/IQR), quality of life (QoL) was 70/55-80 (median/IQR) and no neurologic symptoms (in case of a KPS = 100 or MRS = 0) at admission were present in 23%. This cohort included mostly neuro-oncological patients (n = 161, 85%), with neurological deficit present in 38% and frontal localisation in 34%. Affection for music was reported with an importance of 8/7-8 (median/IQR), only 5% reported never to listen music in daily life. Surgical details showed duration of surgery of 220 minutes and emergency surgery (i.e. within 72 hours) in only 3%.

*Primary outcome*

In the music group, adherence to the music intervention before, during, and directly after surgery was 96%, 100%, and 74%, respectively (supplementary table 1). The following days the adherence decreased each day, from 70% on the first morning to 47% at noon on day 3. The total listening time was a median of 130 minutes (IQR, 73-230) during the five days of admission or until discharge.

A high DOSS score (i.e. 3 or higher) was observed in 32 patients. This was caused by a neurosurgical complication, as confirmed on radiology, in three patients; two patients with infarction after a vascular procedure with hemiparesis and decreased attention. The other patient had a subdural hematoma which was evacuated in the operation room. This resulted in 29 patients with possible delirium by DOSS; 21 were evaluated by the psychiatrist who



For peer review only - http://bmjopen.bmj.com/site/about/guidelines.xhtml

diagnosed delirium in 11 patients (57%, supplementary figure 2) based on the DSM-5 criteria.

According to the DOSS, a significantly higher incidence of delirium was observed in the control (n=21) vs. music (n=11) group in the ITT population for the univariable (22.3 vs. 11.6%, p =0.048) and multivariable (OR/95%CI: 0.49/0.20-1.00, p =0.050) analysis. This was not observed in the mITT (p = 0.064) and the SP (p =0.064) population. The occurrence of a DSM-5 confirmed delirium, was not statistically significant between the control (n=7) vs. music (n=4) group in the ITT (7.4 vs 4.2%, OR = 0.55), the mITT (7.5 vs. 4.4%, OR = 0.57), and the SP (7.4 vs. 4.3%, OR = 0.58) population (table 2).

Of those patients with DSM-5-confirmed delirium (n = 11); severity of delirium (mean/SD) was 12.60/5.52, which was not different between the two arms (p =0.857). The duration of delirium (days, mean/SD) was 3.36/4.69, which was not different between the two arms (p = 0.761).

*Secondary outcome*

Available ECG data (n=87) revealed that heart rate remained constant in the music group while it decreased after 15 ($\beta_1$ = 2.89, p =0.043), 25 ($\beta_1$ = 3.36, p=0.05) and 30 ($\beta_1$ = 5.06, p=0.011) minutes in the control group (table 3 and figure 2). A significant increase on HRV was found by music at 5 minutes on RMSSD ($\beta_1$ = 55.08, p = 0.012). No significant effect was found on the other HRV parameters. Available depth of anaesthesia (n=70) data revealed no significant difference between the music and control group at the several analysed time points (figure 3). A trend towards less anxiety in the music group was observed (p=0.058, figure 4). All other secondary outcomes were not different between groups.

Patient satisfaction (median/IQR) in the music group who filled in the questionnaire (n = 68), was 85/80-95, and 88% reported they would want to receive music in case of future surgery.



For peer review only - http://bmjopen.bmj.com/site/about/guidelines.xhtml

**Discussion**

We found a significant decrease on the incidence of post-operative delirium by the addition of music perioperatively using the DOSS, however this was not significant when assessed by the DSM-5 criteria. Second, music activated pre-operative heart rate variability, a marker of autonomic tone. Last, no significant effect on anxiety, depth of anesthesia, post-operative complications, length of stay and location of discharge were found. Clinical implications and limitations are discussed below.

We found a significant decrease on the incidence of post-operative delirium, defined with DOSS, by the addition of perioperative music. A recent published systematic review conducted a meta-analysis on the preventive effect of music on delirium with six studies and found a relative reduction similar to ours (0.52 vs 0.48)(22) This meta-analysis included pilot studies which administered patient preferred music similar to our design, but varied from ours as the population considered surgical and non-surgical ICU patients and delirium was defined with the NEECHAM, CAM-ICU or own definitions. Our sample size calculation was based on other neurosurgical studies evaluating delirium in case of increased scores on delirium screening tools. (1) When handling screening tools for delirium definition by using the DOSS, we support the efficacy of music in lowering the incidence of post-operative delirium. However, although a similar trend was found, significance of results was not achieved when assessed by the DSM-5 criteria. The discrepancy between DOSS and DSM-5 may have several explanations. First, DSM-5 was evaluated by a psychiatrist *after* an increased DOSS score. Hence, delirium may have been resolved over time before the psychiatrist its assessment. Moreover, DOSS evaluation was conducted three times per day by the nurses, as opposed to DSM-5 determination which was only evaluated once. DSM-5 assessment during day-time might have missed some cases as delirium fluctuates over the course of the day, especially for the delirium-type present during night-time. Also, not all our patients with increased DOSS were evaluated by a psychiatrist due to logistics and we might have missed some patients with delirium. Second, delirium screening tools have not been validated within the neurosurgical population. (5-8, 33-37) Hence, while high diagnostic accuracies in the general population justify diagnostic usage of delirium screening tools, it is unclear whether this can be adopted to our complicated patient population, as a positive screen for delirium



For peer review only - http://bmjopen.bmj.com/site/about/guidelines.xhtml

may be due to the underlying neurological disease or its sequelae (e.g., oedema, vasospasm, seizures, rebleeding, ischemia) leading to false-positive results.

We propose a vagal-mediated anti-inflammatory response as a candidate pathway of music on delirium, as hypotheses of delirium rely on neuro-inflammatory reactions within the brain. (2) Although we did not assess inflammatory cytokines in our study, vagal nerve activation by music was supported by the increased HRV, revealed by an increased RMSSD on ECG during the pre-operative music session. The activation of HRV by music in brain-damaged patients was proven earlier and is considered a valid marker of parasympathetic nervous activation. (38, 39) Whether HRV could be used as a marker for postoperative recovery, remains to be determined. Moreover, we observed a decreasing trend in pre-operative anxiety by music, although this did not reach significance. This pre-operative parasympathetic activation and anxiety reduction may have induced a sedative-sparing effect, subsequently increasing cortical engagement and cognitive processing.(40) We did not find a deeper level of anesthesia in the music group. Literature is contradictory on the correlation of depth of sedation and anesthetic requirements as music listening is associated with decrease of depth of anesthesia, but no decrease of sevoflurane was achieved when pursuing constant depth of anesthesia. Future neurosurgical studies should confirm whether concentration of sedation can be reduced with music in case of standardized depth of anesthesia (BIS) levels.

We found high adherence to the music intervention before surgery. High importance of music in daily life, the number of hours listening to music in daily life and the willingness to receive music intervention in case of future surgeries was found in our cohort, which are considered important facilitators for music implementation. (41) However the adherence declined after surgery, due to pain, nausea, logistics (i.e. for MRI) or unwillingness. Absence of a near future operation prospect may have reduced the urgent necessity of music, resulting in the post-operative decline in adherence. Lack of the knowledge of the intervention is considered a barrier for implementation. Informing patients, substantiated by the results from efficacy studies such as this trial, may aid in the implementation of music in the neurosurgical population.



For peer review only - http://bmjopen.bmj.com/site/about/guidelines.xhtml

Although delirium is (most often) temporary and self-limiting, delirium independently predicts clinically relevant outcomes in neurologically damaged patients. (9) (10) Although a trend was observed, we did not find any significant positive effects on complications, length of admission or location of discharge. Future studies should assess the long-term implications of delirium defined with either DOSS and DSM-criteria after discharge in neurosurgical patients.

*Strengths and limitations*

This is the first randomized controlled trial assessing the effect of music on delirium after craniotomy and the largest assessing the effect of music against delirium. We showed that music reduced the incidence of delirium when defined with the DOSS but not after DSM-5 confirmation. Our study was subject to several limitations: first, not all our patients with increased DOSS were evaluated by a psychiatrist due to logistics and we might have missed some patients with delirium. However, we feel that this did not affect our conclusions, because, with our low confirmation rate of suspected delirium, the study would still have been underpowered. Second, due to the nature of the intervention we did not blind the study, which could have influenced the outcome assessors. However, blinding could not be secured which might have led to misleading results. Third, the generalizability of the results may be affected by the single-centre design of the study.

For peer review only

## Conclusion

Our results support the efficacy of music in reducing the incidence of delirium after craniotomy, as found with DOSS but not after DSM-5 confirmation. Delirium screening tools should be validated within the neurosurgical context and the long-term implications of a delirium, either defined by an increased DOSS or DSM-5, should be evaluated. This effect of music is substantiated by the effect of music on an increased pre-operative HRV. Last, although pre-operative adherence was high, this declined after surgery which should be taken into account, when considering implementation in the neurosurgical population.

## Contributors

Each author has contributed significantly to, and is willing to take public responsibility for, one or more aspects of the study. AJPEV, CMFD, JJ, MK and PRK conceived the study idea. PRK coordinated the research protocol. PRK and MIM extracted the data. PRK, BvdH and AJPEV conducted the statistical analysis and wrote the first draft of the manuscript. JJ, MIM, CMFD, MK, SK, R-JO, MC, MJP and AJPEV critically revised the manuscript. All authors have seen and approved the final version of the manuscript being submitted. The article is the authors' original work, has not received prior publication and is not under consideration for publication elsewhere.

## Funding

This research is funded with the Erasmus MC Efficiency Grant (Grant number: 19105) by the Erasmus Medical Centre, Rotterdam, The Netherlands.

## Competing interests

None declared.

## Research ethical approval

This study involves human participants and was approved by the Medical Ethics Review Committee of the Erasmus Medical Center, Rotterdam (Erasmus MC), which declared this study not subject to the Medical Research Involving Human Subjects Act (i.e. 'Non-WMO'), and approved this study (MEC-2020-0064), on the 25th of February of 2020, as such. Written informed consent was obtained for each inclusion by patient or legal representative



For peer review only - http://bmjopen.bmj.com/site/about/guidelines.xhtml

**Data Sharing Statement**

The raw data supporting the conclusions of this article will be made available by the authors, without undue reservation.

For peer review only



For peer review only - http://bmjopen.bmj.com/site/about/guidelines.xhtml

**Table 1. Baseline characteristics**

| | Control (n=94) | Music (n = 95) |
|---|---|---|
| **Pre-hospital demographics** | | |
| Age (years) * | 61 (51-69) | 60 (49-69) |
| Sex (% female) | 46 (49%) | 38 (40%) |
| Medical history (n/%) | | |
| Somatic history[1] | 79 (84%) | 74 (78%) |
| Psychiatric history | 14 (15%) | 6 (6%) |
| Delirium prior admission | 2 (2%) | 5 (3%) |
| Dementia | 0 (0%) | 0 (0%) |
| Body mass index ($kg/m^2$) | 26 (24-30) | 26 (23-28) |
| Medication (n/%)[2] | 15 (26%) | 14 (15%) |
| Intoxication[3] | | |
| Abuse of alcohol (n/%) | 6 (6%) | 1 (1%) |
| Abuse of drugs (n/%) | 3 (3%) | 3 (3%) |
| **In-hospital demographics** | | |
| Pre-hospital functional status[4] | 80 (70-90) | 80 (70-90) |
| KPS (100-0) | 70 (18-80) | 70 (45-80) |
| MRS (5-1) | 1.0 (0.25-2.0) | 1.0 (1.0-2.0) |
| Quality of Life (1- 100) | 70 (60-79) | 70 (55-80) |
| Cognitive function (0 - 30)[5] | 24 (19-27) | 25 (21-27) |
| Electrolyte disturbance (n/%)[6] | 10 (11%) | 9 (10%) |
| | | |
| **Disease characteristics** | | |
| Neurologic deficit (n/%) | 35 (37%) | 36 (38%) |
| Type | | |
| Oncologic | 81 (86%) | 80 (84%) |
| Vascular | 12 (13%) | 14 (15%) |
| Other | 1 (1%) | 1 (1%) |
| Frontal disease localization (n/%) | 40 (43%) | 24 (25%) |
| **Music affection** | | |
| Music importance | 7.0 (6.8-8.0) | 8.0 (7.0-8.0) |
| Frequency listening | | |
| The whole day | 29 (31%) | 21 (22%) |
| Some hours per day | 44 (47%) | 54 (57%) |
| Some hours per week | 9 (10%) | 12 (13%) |
| Never | 6 (6%) | 3 (3%) |
| Played an instrument | 20 (21%) | 20 (21%) |
| | | |
| **Operation details[7]** | | |
| Operation duration (minutes) | 220 (160-320) | 210 (140-290) |
| Emergency operation (n/%)[8] | 2 (2%) | 4 (4%) |
| Supine position (n/%) | 81 (86%) | 80 (84%) |
| Tramrail sign tension (n/%) | 68 (72%) | 67 (7%) |

*All continuous data are presented in median/IQR. 1. Somatic history: including systematic disease (DM, hypertension) currently treated by medication and prior surgery (requiring general anesthesia on the operation room). 2. Medication known to induce delirium before admission, such as sleep medication, morphine, atropine and antidepressants 3. Reported abusive use of alcohol and/or drugs. 4. Patients' functional performance with the Karnofsky Performance Scale (ranging from 100/'No complaints' to 0/'Death') and Modified Ranking Scale (ranging from 0/'No symptoms to 5/'Death'. 5. Cognitive function assessed with the Montreal Cognitive Assessment / MoCA 6. Electrolyte disturbance (mEq/L) in case of sodium >145 or <135 or potassium <3.5 or >5. 7. Patients in the MITT population (n=184) only. 8. Operation indication within 72 hours.

For peer review only

**Table 2. Primary outcome**

| | Control (n/%) | Music (n/%) | Univariable analysis (OR/95%CI)* | P value[1] | Multivariable analysis (OR/95%CI)* | P value[2] |
|---|---|---|---|---|---|---|
| **Intention-to-treat analysis (ITT)** | | | | | | |
| Increased DOSS | 21/22.3% | 11/11.6% | 0.46/0.19;1.00 | 0.048 | 0.49/0.20;1.00 | 0.050 |
| Confirmed by DSM-5 | 7/7.4% | 4 /4.2% | 0.55/0.14;1.96 | 0.342 | 0.57/0.16;2.07 | 0.39 |
| **Modified Intention-to-treat analysis (mITT)** | | | | | | |
| Increased DOSS | 21/22.6% | 11/12.1% | 0.47/0.21;1.04 | 0.060 | 0.47/0.16;2.07 | 0.064 |
| Confirmed by DSM-5 | 7/7.5% | 4/4.4% | 0.57/0.14;2.03 | 0.370 | 0.58/0.16;2.10 | 0.412 |
| **Safety Population (SP)** | | | | | | |
| Increased DOSS | 21/22.3% | 11/12.0% | 0.47/0.21;1.04 | 0.061 | 0.47/0.21;1.04 | 0.064 |
| Confirmed by DSM-5 | 7/7.4% | 4/4.3% | 0.58/0.14;2.03 | 0.370 | 0.58/0.16;2.11 | 0.414 |

*Odds Ratio/95%Confidence interval (OR/95%CI). 1. P values assessed with the Chi-squared test 2. Logistic regression analysis with groups, type disease and gender as independent variables.



For peer review only - http://bmjopen.bmj.com/site/about/guidelines.xhtml

**Table 3. Secondary outcomes**

| | Control (n = 93) | Music (n = 91) | P value |
|---|---|---|---|
| **Univariable analyses** | | | |
| Anxiety difference (mean/SD)[1] | 0.05/0.94 | -0.25/1.49 | 0.058 |
| Pain (mean/SD)[2] | 3.56/1.91 | 3.16/1.74 | 0.246 |
| Naproxen mg (mean/SD) | 13.6/75.4 | 2.75/26.2 | 0.103 |
| Oxycodon mg (mean/SD) | 2.03/4.35 | 1.61/3.31 | 0.828 |
| No complications (n/%) | 25 (26.9%) | 21 (23.1%) | 0.551 |
| Length of stay, days (mean/SD) | 7.43 (8.08) | 6.74 (8.26) | 0.947 |
| Discharge home (n/%) | 77 (82.8%) | 76 (83.5%) | 0.896 |

**Multivariable analyses**

| Heart Rate Variability/HRV ($\beta_1$/95%CI)[4] | | | | |
|---|---|---|---|---|
| Time point[3] | SDNN | RMSSD | NN50 | LF/HF |
| 5 minutes | 38.84/ -2.23;79.91 | 55.08/13.16;97.00* | 17.64/ -3.92;39.21 | -0.46/-1.04;0.11 |
| 10 minutes | 18.87/ -18.23;55.97 | 18.79/ -16.16;53.75 | 11.11/ -8.67;30.88 | 0.49/ -0.18;1.17 |
| 15 minutes | 2.49/ -35.41;40.38 | -3.20/ -38.60;32.19 | 9.14/ -11.11;29.38 | 0.34/ -0.28;0.96 |
| 20 minutes | -11.65/ -52.87;29.58 | 3.12/ -40.54;46.78 | 8.22/ -12.00;28.45 | 0.56/ -0.07;1.19 |
| 25 minutes | -15.62/-57.24;26.00 | -11.85/ -58.03;34.33 | -3.56/ -27.10;19.98 | 0.45/-0.26;1.16 |
| 30 minutes | 7.41/-38.04;52.87 | 22.20/ -17.01;61.41 | -5.98/ -28.49;16.53 | 0.46/-0.18;1.09 |

| Time point[3] | Depth of anesthesia/BIS ($\beta_1$/95%CI)[5] | P value |
|---|---|---|
| 15 minutes | 0.71/ -3.17;4.58 | 0.717 |
| 30 minutes | -1.44/ -3.34;0.46 | 0.139 |
| 45 minutes | -1.06/ -3.42;1.30 | 0.378 |
| 60 minutes | -2.23/ -5.34;0.89 | 0.162 |
| 75 minutes | -1.82/ -5.48;1.83 | 0.328 |
| 90 minutes | -2.46/ -6.72;1.79 | 0.256 |
| 105 minutes | -0.50/ -6.17;5.16 | 0.862 |
| 120 minutes | 0.31/ -5.44;6.05 | 0.917 |

Secondary outcomes analyzed on the mITT population. 1. Anxiety differences between first and second measures with VAS-A. 2. Pain (NRS) over the first three post-operative days. 3. Time-points samples included in the linear mixed model. 4. HRV analyses; 30 minute of pre-operative ECG recordings split into 5-minute samples, all values marked with * are significant (i.e. p<0.05). 5. BIS analyses: 120 minute of intra-operative BIS registration split into 15-minute samples.

For peer review only



For peer review only - http://bmjopen.bmj.com/site/about/guidelines.xhtml

**Figure 1.** CONSORT Trial flowchart.

**Figure 2.** Pre-operative course of HRV. A 30-minute recording of HRV was split into samples of 5 minutes as time points and compared between groups. Heart rate remained constant in the music group while it decreased after 15 ($\beta_1$ = 2.89, p =0.043), 25 ($\beta_1$ = 3.36, p=0.05) and 30 ($\beta_1$ = 5.06, p=0.011) minutes in the control group. A significant increase on HRV was found by music at 5 minutes on RMSSD ($\beta_1$ = 55.08, p = 0.012). No significant effect was found on the other HRV parameters.

**Figure 3**. Intra-operative depth of anaesthesia. A 2-hour recording of BIS was split into samples of 15 minutes as time points and compared between groups. Available depth of anaesthesia (n=70) data revealed no significant difference between the music and control group at the several analysed time points (figure 3).

**Figure 4.** Two anxiety measurements (VAS-A) were conducted before surgery and categorized in 'No anxiety' (0 – 2), 'Medium anxiety (3-6) and 'High anxiety (7 – 10). Most patients remained in their anxiety level, although some patients showed a decrease pre-operative anxiety when receiving music, but this difference was non-significant between groups (p=0.058).

**Supplementary figure 1. Study course MUSYC trial.**

**Supplementary figure 2. Course delirium diagnosis.**

**Supplementary table 1. Adherence to music intervention during trial.**

## References

For peer review only

1. Kappen PR, Kakar E, Dirven CMF, van der Jagt M, Klimek M, Osse RJ, et al. Delirium in neurosurgery: a systematic review and meta-analysis. Neurosurg Rev. 2021;45(1):329-341.
2. Maldonado JR. Neuropathogenesis of delirium: review of current etiologic theories and common pathways. Am J Geriatr Psychiatry. 2013;21(12):1190-222.
3. Oh ES, Fong TG, Hshieh TT, Inouye SK. Delirium in Older Persons: Advances in Diagnosis and Treatment. JAMA. 2017;318(12):1161-74.
4. Flanigan PM, Jahangiri A, Weinstein D, Dayani F, Chandra A, Kanungo I, et al. Postoperative Delirium in Glioblastoma Patients: Risk Factors and Prognostic Implications. Neurosurgery. 2018;83(6):1161-72.
5. He Z, Cheng H, Wu H, Sun G, Yuan J. Risk factors for postoperative delirium in patients undergoing microvascular decompression. PLoS One. 2019;14(4):e0215374.
6. Wang CM, Huang HW, Wang YM, He X, Sun XM, Zhou YM, et al. Incidence and risk factors of postoperative delirium in patients admitted to the ICU after elective intracranial surgery: A prospective cohort study. Eur J Anaesthesiol. 2020;37(1):14-24.
7. Zipser Z, Deuel J, Ernst J, Schubert M, von Kanel R, Bottger S. The predisposing and precipitating risk factors for delirium in neurosurgery: a prospective cohort study of 949 patients. Acta Neurochir (Wien). 2019;161(7):1307-15.
8. Kappen PR, Kappen HJ, Dirven CMF, Klimek M, Jeekel J, Andrinopoulou ER, et al. Postoperative Delirium After Intracranial Surgery: A Retrospective Cohort Study. World Neurosurgery. 2023;(22)01838-1.
9. Budėnas A, Tamašauskas Š, Šliaužys A, Navickaitė I, Sidaraitė M, Pranckevičienė A, et al. Incidence and clinical significance of postoperative delirium after brain tumor surgery. Acta Neurochir. 2018;160(12):2327-37.
10. Chen H, Jiang H, Chen B, Fan L, Shi W, Jin Y, et al. The Incidence and Predictors of Postoperative Delirium After Brain Tumor Resection in Adults: A Cross-Sectional Survey. World Neurosurg. 2020;140:e129-e39.
11. Liu Y, Li XJ, Liang Y, Kang Y. Pharmacological Prevention of Postoperative Delirium: A Systematic Review and Meta-Analysis of Randomized Controlled Trials. Evid Based Complement Alternat Med. 2019;2019:9607129.
12. Vlisides P, Avidan M. Recent Advances in Preventing and Managing Postoperative Delirium. F1000Res. 2019;8.
13. Inouye SK, Westendorp RG, Saczynski JS. Delirium in elderly people. Lancet. 2014;383(9920):911-22.
14. Cerejeira J, Batista P, Nogueira V, Vaz-Serra A, Mukaetova-Ladinska EB. The stress response to surgery and postoperative delirium: evidence of hypothalamic-pituitary-adrenal axis hyperresponsiveness and decreased suppression of the GH/IGF-1 Axis. J Geriatr Psychiatry Neurol. 2013;26(3):185-94.
15. Clegg A YJ. Which medications to avoid in people at risk of delirium: a systematic review. 2011(40:23–29).
16. Fu VX, Oomens P, Sneiders D, van den Berg SAA, Feelders RA, Wijnhoven BPL, et al. The Effect of Perioperative Music on the Stress Response to Surgery: A Meta-analysis. J Surg Res. 2019;244:444-55.
17. Fu VX OP, Klimek M, Verhofstad MHJ, Jeekel J. The effect of perioperative music on medication requirement and hospital length of stay: a meta-analysis. Annals of Surgery. July 2019;272(6):961 - 72.
18. Kuhlmann AYR, de Rooij A, Kroese LF, van Dijk M, Hunink MGM, Jeekel J. Meta-analysis evaluating music interventions for anxiety and pain in surgery. Br J Surg. 2018;105(7):773-83.
19. Lee JH. The Effects of Music on Pain: A Meta-Analysis. J Music Ther. 2016;53(4):430-77.
20. Leung JM SL, Paul S. Does postoperative delirium limit the use of patient-controlled analgesia in older surgical patients? 111:625–631. 2009.



For peer review only - http://bmjopen.bmj.com/site/about/guidelines.xhtml

21. Hole J, Hirsch M, Ball E, Meads C. Music as an aid for postoperative recovery in adults: a systematic review and meta-analysis. Lancet. 2015;386(10004):1659-71.
22. Golubovic J, Neerland BE, Aune D, Baker FA. Music Interventions and Delirium in Adults: A Systematic Literature Review and Meta-Analysis. Brain Sci. 2022;12(5).
23. Kappen P, Jeekel J, Dirven CMF, Klimek M, Kushner S, Osse RJ, et al. Music to prevent deliriUm during neuroSurgerY (MUSYC) Clinical trial: a study protocol for a randomised controlled trial. BMJ Open. 2021;11(10):e048270.
24. Chan AW, Tetzlaff JM, Altman DG, Laupacis A, Gotzsche PC, Krleza-Jeric K, et al. SPIRIT 2013 statement: defining standard protocol items for clinical trials. Ann Intern Med. 2013;158(3):200-7.
25. Janssen TL, Alberts AR, Hooft L, Mattace-Raso F, Mosk CA, van der Laan L. Prevention of postoperative delirium in elderly patients planned for elective surgery: systematic review and meta-analysis. Clin Interv Aging. 2019;14:1095-117.
26. Abraha I, Trotta F, Rimland JM, Cruz-Jentoft A, Lozano-Montoya I, Soiza RL, et al. Efficacy of Non-Pharmacological Interventions to Prevent and Treat Delirium in Older Patients: A Systematic Overview. The SENATOR project ONTOP Series. PLoS One. 2015;10(6):e0123090.
27. Lee H, Ju JW, Oh SY, Kim J, Jung CW, Ryu HG. Impact of timing and duration of postoperative delirium: a retrospective observational study. Surgery. 2018;164(1):137 - 43.
28. Association AP. Diagnostic and Statistical Manual of Mental Disorders, Fourth Edition. May 2013.
29. Gavinski K, Carnahan R, Weckmann M. Validation of the delirium observation screening scale in a hospitalized older population. J Hosp Med. 2016;11(7):494-7.
30. Detroyer E, Clement PM, Baeten N, Pennemans M, Decruyenaere M, Vandenberghe J, et al. Detection of delirium in palliative care unit patients: a prospective descriptive study of the Delirium Observation Screening Scale administered by bedside nurses. Palliat Med. 2014;28(1):79-86.
31. van Velthuijsen EL, Zwakhalen SM, Warnier RM, Mulder WJ, Verhey FR, Kempen GI. Psychometric properties and feasibility of instruments for the detection of delirium in older hospitalized patients: a systematic review. Int J Geriatr Psychiatry. 2016;31(9):974-89.
32. American Psychiatric A. American Psychiatric Association DSM-5 Task Force (2013): Diagnostic and Statistical Manual of Mental Disorders: DSM-5. Washington, DC: American Psychiatric Association.
33. Chen L, Xu M, Li GY, Cai WX, Zhou JX. Incidence, Risk Factors and Consequences of Emergence Agitation in Adult Patients after Elective Craniotomy for Brain Tumor: A Prospective Cohort Study. PLoS One. 2014;9(12):e114239.
34. Flanigan PM, Jahangiri A, Kuang R, Truong A, Choi S, Chou A, et al. Developing an Algorithm for Optimizing Care of Elderly Patients With Glioblastoma. Neurosurgery. 2018;82(1):64-75.
35. Herrero S, Carrero E, Valero R, Rios J, Fabregas N. Postoperative surveillance in neurosurgical patients - usefulness of neurological assessment scores and bispectral index. Rev Bras Anestesiol. 2017;67(2):153-65.
36. Matano F, Mizunari T, Yamada K, Kobayashi S, Murai Y, Morita A. Environmental and Clinical Risk Factors for Delirium in a Neurosurgical Center: A Prospective Study. World Neurosurg. 2017;103:424-30.
37. Wang J, Ji Y, Wang N, Chen W, Bao Y, Qin Q, et al. Establishment and validation of a delirium prediction model for neurosurgery patients in intensive care. Int J Nurs Pract. 2020:e12818.
38. Florez-Perdomo WA, Garcia-Ballestas E, Moscote-Salazar LR, Konar SK, Raj S, Chouksey P, et al. Heart Rate Variability as a Predictor of Mortality in Traumatic Brain Injury: A Systematic Review and Meta-Analysis. World Neurosurg. 2021;148:80-9.
39. Mojtabavi H, Saghazadeh A, Valenti VE, Rezaei N. Can music influence cardiac autonomic system? A systematic review and narrative synthesis to evaluate its impact on heart rate variability. Complement Ther Clin Pract. 2020;39:101162.



For peer review only - http://bmjopen.bmj.com/site/about/guidelines.xhtml

40. Khan SH, Wang S, Harrawood A, Martinez S, Heiderscheit A, Chlan L, et al. Decreasing Delirium through Music (DDM) in critically ill, mechanically ventilated patients in the intensive care unit: study protocol for a pilot randomized controlled trial. Trials. 2017;18(1):574.
41. Kakar E, Ista E, Klimek M, Jeekel J. Implementation of music in the perioperative standard care of colorectal surgery: study protocol of the IMPROVE Study. BMJ Open. 2021;11(10):e051878.

For peer review only

**Figure 1. Trial flowchart**

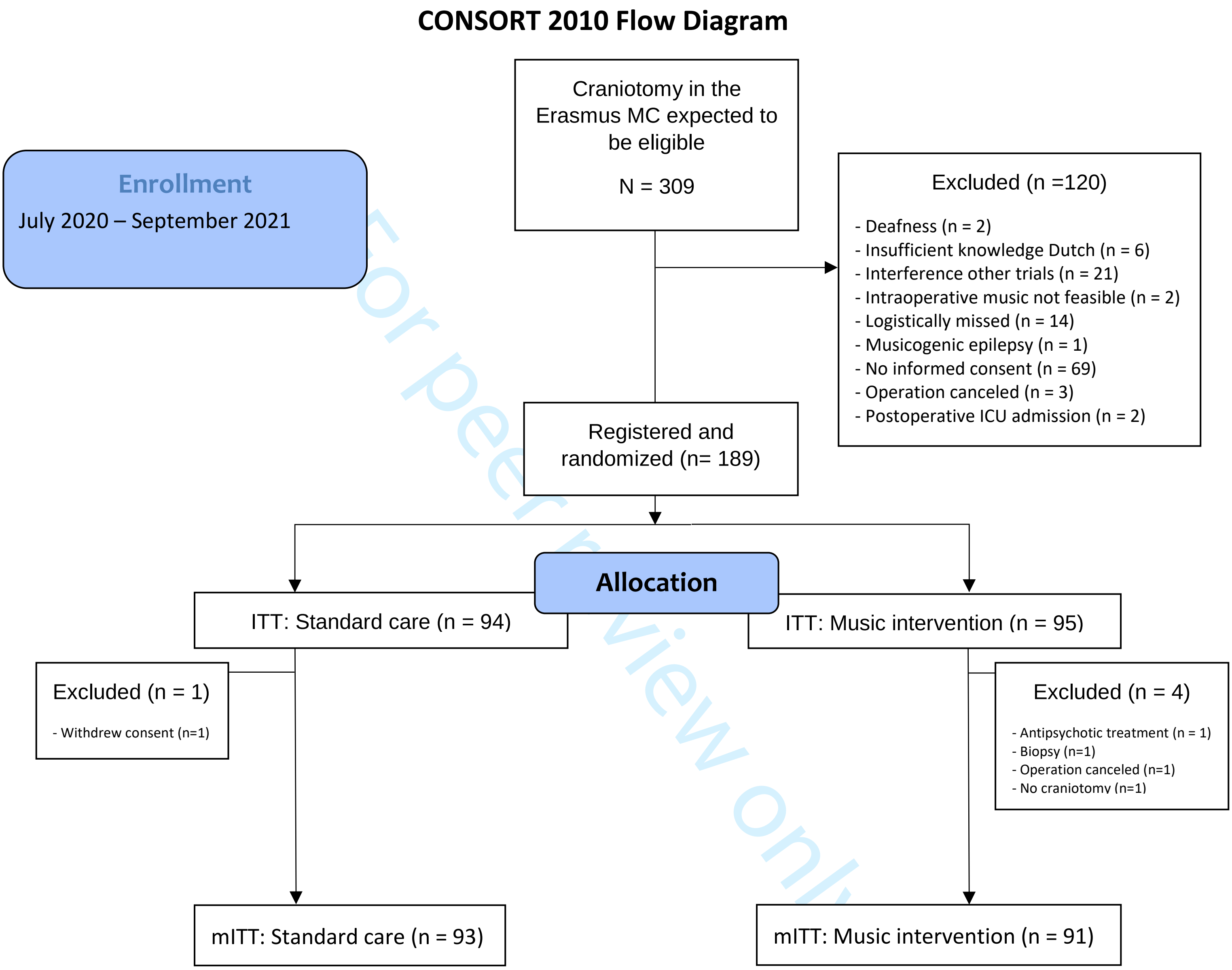


For peer review only

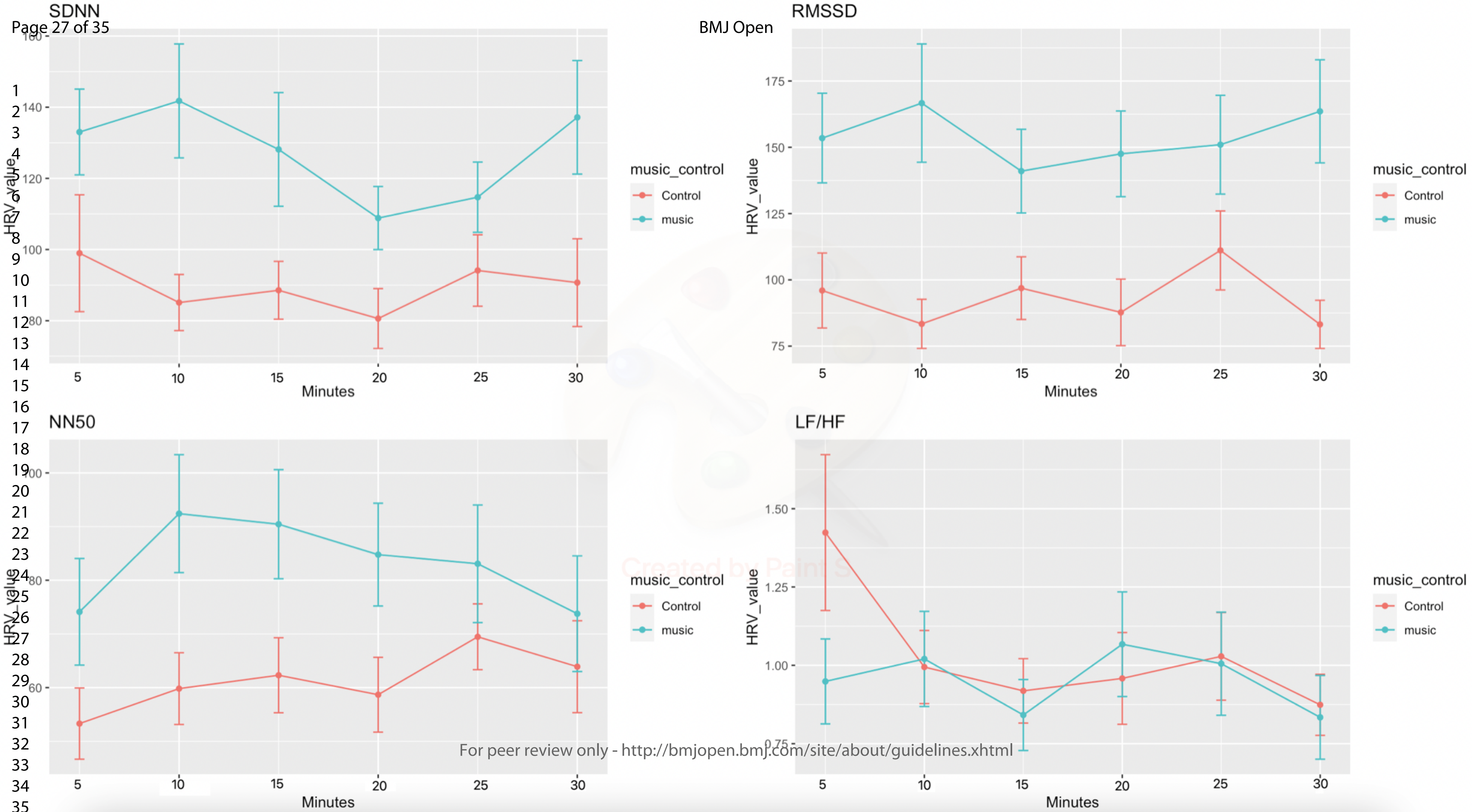
SDNN
HRV_value
Minutes
music_control
Control
music
RMSSD
HRV_value
Minutes
music_control
Control
music
NN50
HRV_value
Minutes
music_control
Control
music
LF/HF
HRV_value
Minutes
music_control
Control
music

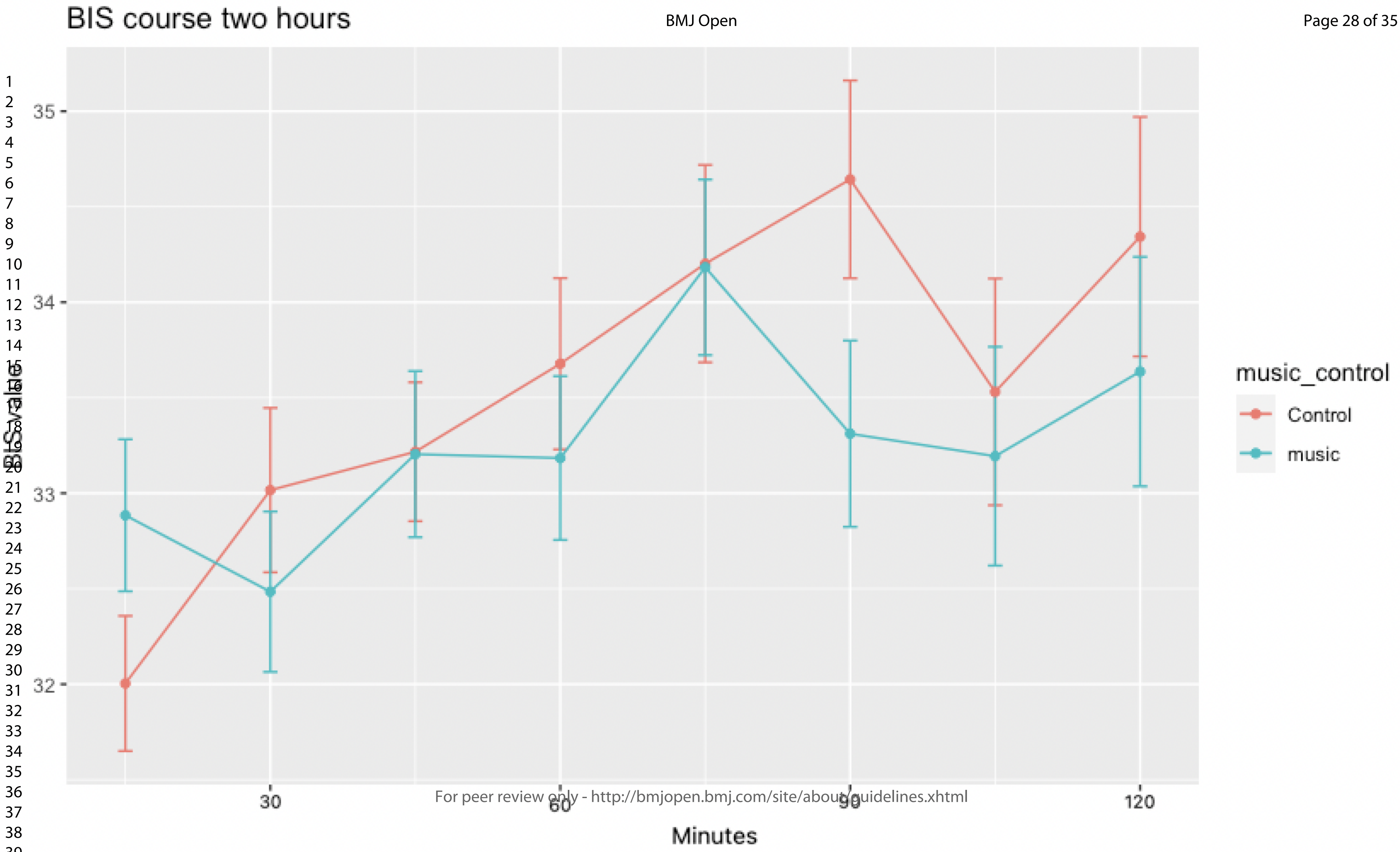
BIS course two hours
35
34
33
32
BIS value
30
60
90
120
Minutes
music_control
Control
music

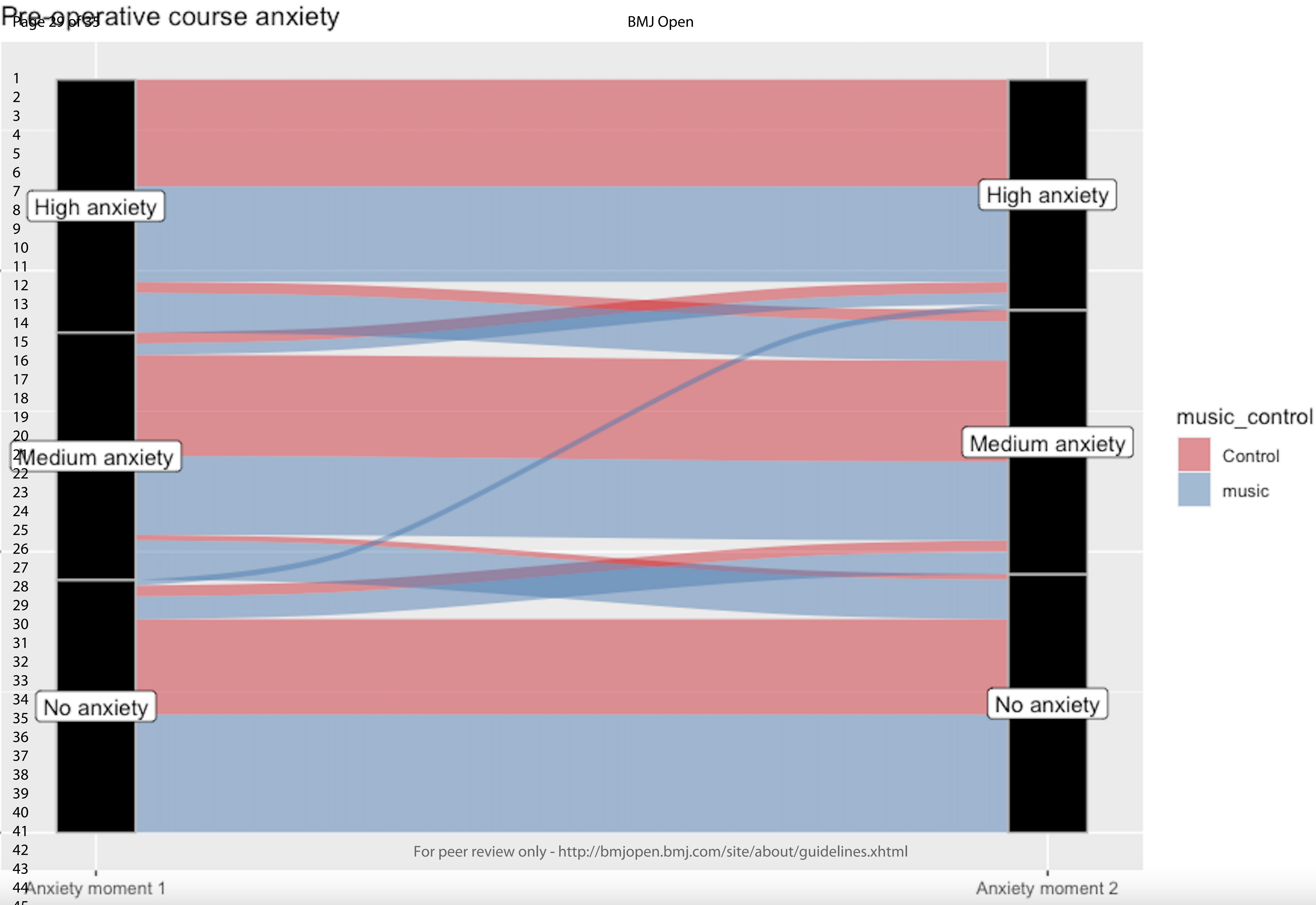
Pre-operative course anxiety
High anxiety
Medium anxiety
No anxiety
High anxiety
Medium anxiety
No anxiety
music_control
Control
music
Anxiety moment 1
Anxiety moment 2

**Supplementary figure 1. Study course MUSYC trial**

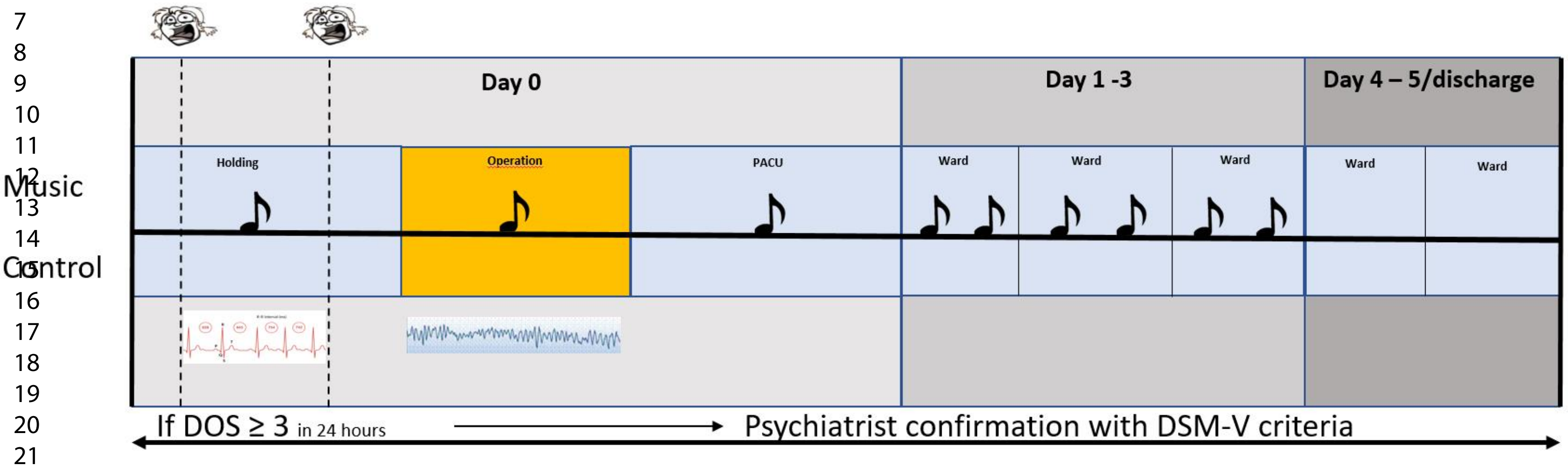


Course trial: after randomization, both groups receive ECG recording and two times anxiety measures. Intra-operative BIS registration is applied for depth of anesthesia. All participating subjects are treated according to standard care and delirium is measured until day 5 or discharge. Participants in the intervention group additionally receive music intervention 30 minutes before surgery, intra-operative during the entire operation, 30 minutes after surgery and twice per day 30minutes until post-operative day 3.

For peer review only

**Supplementary figure 2. Course delirium diagnosis**

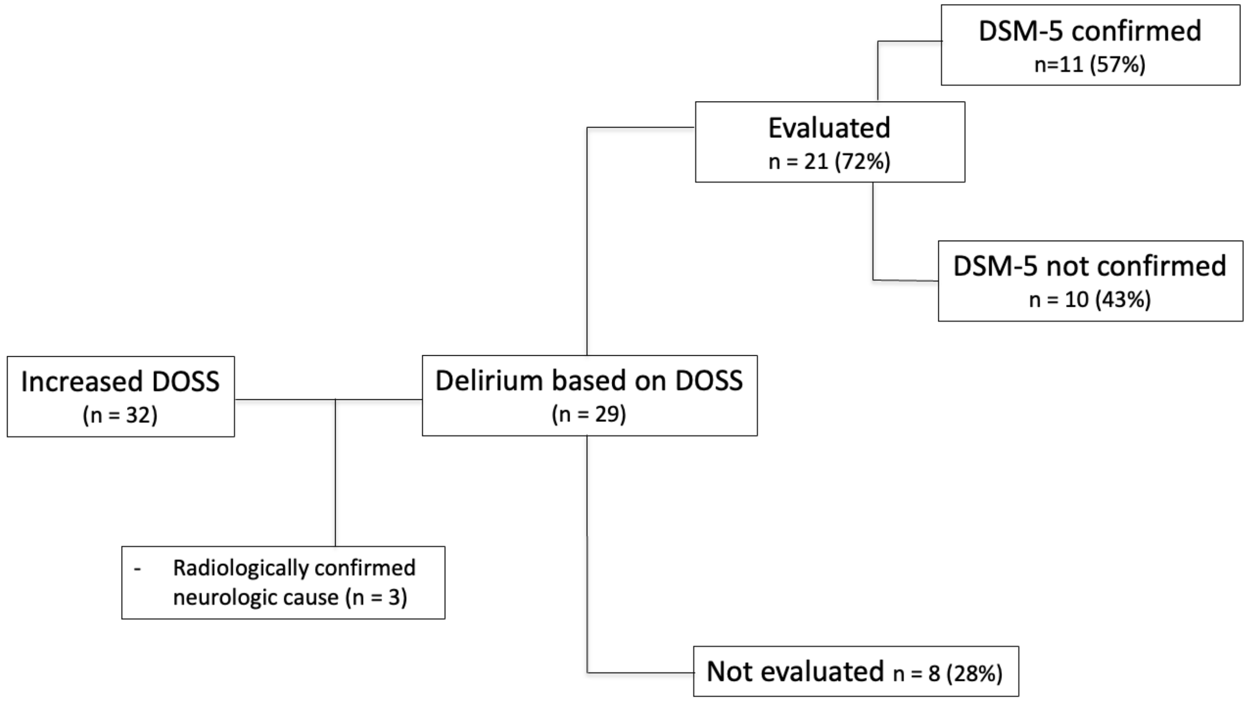


Delirium diagnosis flowchart, diagnosis of delirium required a two-step procedure. First, all participants were screened with the DOS scale. Second, in case of a daily mean score of 3 or higher (and no neurologic cause was radiologically found) a psychiatrist was consulted to assess the clinical diagnosis of delirium based on the DSM-5 criteria. In 8 patients, the psychiatrist was not consulted due to logistic issues.

view only

**Supplementary table 1. Adherence to music intervention during trial**

| | Day 0 | | | Day 1 | | Day 2 | | Day 3 | |
|---|---|---|---|---|---|---|---|---|---|
| **Moment[1]** | **Pre** | **Per** | **Post** | **Morning** | **Noon** | **Morning** | **Noon** | **Morning** | **Noon** |
| **N[2]** | 91 | 91 | 91 | 91 | 91 | 88 | 88 | 70 | 70 |
| | | | | | | | | | |
| **Music** | | | | | | | | | |
| **No** | 3 | 0 | 21 | 25 | 26 | 26 | 21 | 19 | 20 |
| **Yes** | 79 | 91 | 61 | 59 | 45 | 44 | 39 | 24 | 18 |
| **Unknown[3]** | 9 | 0 | 9 | 7 | 20 | 18 | 28 | 27 | 32 |
| **Adherence[4]** | 96% | 100% | 74% | 70% | 63% | 63% | 65% | 56% | 47% |

Patients in the mITT population allocated to the music group. 1. Session of 30 minutes in the morning or afternoon. 2. Amount of patients in the musical group not discharged from the ward. 3. Unregistered music session. 4. Adherence calculated from the registered patients (i.e. 'Yes'/'No' + 'Yes')

For peer review only

**2017 CONSORT checklist of information to include when reporting a randomized trial assessing nonpharmacologic treatments (NPTs)*. Modifications of the extension appear in italics and blue.**

| Section/Topic Item | Checklist item no. | CONSORT item | Extension for NPT trials | Page |
|---|---|---|---|---|
| **Title and abstract** | | | | |
| | 1a | Identification as a randomized trial in the title | | 1 |
| | 1b | Structured summary of trial design, methods, results, and conclusions (for specific guidance see CONSORT for abstracts) | *Refer to CONSORT extension for abstracts for NPT trials* | *3* |
| **Introduction** | | | | |
| Background and objectives | 2a | Scientific background and explanation of rationale | | 4, 5 |
| | 2b | Specific objectives or hypotheses | | 4, 5 |
| **Methods** | | | | |
| Trial design | 3a | Description of trial design (such as parallel, factorial) including allocation ratio | When applicable, how care providers were allocated to each trial group | 6:10 |
| | 3b | Important changes to methods after trial commencement (such as eligibility criteria), with reasons | | 6, 8 |
| Participants | 4a | Eligibility criteria for participants | When applicable, eligibility criteria for centers and for *care providers* | 6 |
| | 4b | Settings and locations where the data were collected | | 6 |
| Interventions *†* | 5 | The interventions for each group with sufficient details to allow replication, including how and when they were actually administered | Precise details of both the experimental treatment and comparator | 7, 8 |
| | 5a | | Description of the different components of the interventions and, when applicable, description of the procedure for tailoring the interventions to individual participants. | 7, supplementary figures |
| | 5b | | Details *of whether and* how the interventions were standardized. | 7, supplementary figures |
| | 5c. | | Details *of whether and* how adherence of care providers to the protocol was assessed or enhanced | 8, supplementary figures |

*Cite as:* *Boutron I, Altman DG, Moher D, Schulz KF, Ravaud P. CONSORT Statement for Randomized Trials of Nonpharmacologic Treatments: A 2017 Update and a CONSORT Extension for Nonpharmacologic Trial Abstracts. Annals of Internal Medicine. 2017 Jul 4;167(1):40–7.*

| Section/Topic Item | Checklist item no. | CONSORT item | Extension for NPT trials | Page |
|---|---|---|---|---|
| | *5d* | | *Details of whether and how adherence of participants to interventions was assessed or enhanced* | *8* |
| Outcomes | 6a | Completely defined pre-specified primary and secondary outcome measures, including how and when they were assessed | | **8, 9** |
| | 6b | Any changes to trial outcomes after the trial commenced, with reasons | | **7, 9** |
| Sample size | 7a | How sample size was determined | When applicable, details of whether and how the clustering by care providers or centers was addressed | 9, 10 |
| | 7b | When applicable, explanation of any interim analyses and stopping guidelines | | NA |
| Randomization: | | | | 6, 7 |
| - Sequence generation | 8a | Method used to generate the random allocation sequence | | 6, 7 |
| | 8b | Type of randomization; details of any restriction (such as blocking and block size) | | 6, 7 |
| - Allocation concealment mechanism | 9 | Mechanism used to implement the random allocation sequence (such as sequentially numbered containers), describing any steps taken to conceal the sequence until interventions were assigned | | 6, 7 |
| - Implementation | 10 | Who generated the random allocation sequence, who enrolled participants, and who assigned participants to interventions | | 6, 7 |
| Blinding | 11a | If done, who was blinded after assignment to interventions (for example, participants, care providers, those assessing outcomes) and how | If done, who was blinded after assignment to interventions (e.g., participants, care providers, *those administering co-interventions,* those assessing outcomes) and how | 8 |
| | 11b | If relevant, description of the similarity of interventions | If blinded, method of blinding and description of the similarity of interventions | NA |
| | *11c* | | *If blinding was not possible, description of any attempts to limit bias* | *8* |

***Cite as:*** *Boutron I, Altman DG, Moher D, Schulz KF, Ravaud P. CONSORT Statement for Randomized Trials of Nonpharmacologic Treatments: A 2017 Update and a CONSORT Extension for Nonpharmacologic Trial Abstracts. Annals of Internal Medicine. 2017 Jul 4;167(1):40–7.*

| Section/Topic Item | Checklist item no. | CONSORT item | Extension for NPT trials | Page |
|---|---|---|---|---|
| Statistical methods | 12a | Statistical methods used to compare groups for primary and secondary outcomes | When applicable, details of whether and how the clustering by care providers or centers was addressed | 9, 10 |
| | 12b | Methods for additional analyses, such as subgroup analyses and adjusted analyses | | 9, 10 |
| **Results** | | | | |
| Participant flow (a diagram is strongly recommended) | 13a | For each group, the numbers of participants who were randomly assigned, received intended treatment, and were analyzed for the primary outcome | The number of care providers or centers performing the intervention in each group and the number of patients treated by each care provider or in each center | 11, figure 1 |
| | 13b | For each group, losses and exclusions after randomization, together with reasons | | 11, figure 1 |
| | 13c | | *For each group, the delay between randomization and the initiation of the intervention* | *Supplementary figures* |
| | new | | Details of the experimental treatment and comparator as they were implemented | *Supplementary figures* |
| Recruitment | 14a | Dates defining the periods of recruitment and follow-up | | **11, figure 1** |
| | 14b | Why the trial ended or was stopped | | **11** |
| Baseline data | 15 | A table showing baseline demographic and clinical characteristics for each group | When applicable, a description of care providers (case volume, qualification, expertise, etc.) and centers (volume) in each group. | Table 1 |
| Numbers analyzed | 16 | For each group, number of participants (denominator) included in each analysis and whether the analysis was by original assigned groups | | 11, 12, Table 1 |
| Outcomes and estimation | 17a | For each primary and secondary outcome, results for each group, and the estimated effect size and its precision (such as 95% confidence interval) | | 11, 12, table 2, 3 and figure 2, 3 and 4 |
| | 17b | For binary outcomes, presentation of both absolute and relative effect sizes is recommended | | 11, 12, ttable 2, 3 and figure 2, 3 and 4 |
| Ancillary analyses | 18 | Results of any other analyses performed, including subgroup analyses and adjusted analyses, distinguishing pre-specified from exploratory | | 11, 12, table 2, 3 and figure 2, 3 and 4 |

***Cite as:** Boutron I, Altman DG, Moher D, Schulz KF, Ravaud P. CONSORT Statement for Randomized Trials of Nonpharmacologic Treatments: A 2017 Update and a CONSORT Extension for Nonpharmacologic Trial Abstracts. Annals of Internal Medicine. 2017 Jul 4;167(1):40–7.*

For peer review only - http://bmjopen.bmj.com/site/about/guidelines.xhtml

| Section/Topic Item | Checklist item no. | CONSORT item | Extension for NPT trials | Page |
|---|---|---|---|---|
| Harms | 19 | All important harms or unintended effects in each group (for specific guidance see CONSORT for harms) | | NA |
| **Discussion** | | | | |
| Limitations | 20 | Trial limitations, addressing sources of potential bias, imprecision, and, if relevant, multiplicity of analyses | In addition, take into account the choice of the comparator, lack of or partial blinding, and unequal expertise of care providers or centers in each group | 15 |
| Generalizability | 21 | Generalizability (external validity, applicability) of the trial findings | Generalizability (external validity) of the trial findings according to the intervention, comparators, patients, and care providers and centers involved in the trial | 13-15 |
| Interpretation | 22 | Interpretation consistent with results, balancing benefits and harms, and considering other relevant evidence | | 13-15 |
| **Other information** | | | | |
| Registration | 23 | Registration number and name of trial registry | | 6 |
| Protocol | 24 | Where the full trial protocol can be accessed, if available | | 6 |
| Funding | 25 | Sources of funding and other support (such as supply of drugs), role of funders | | 16 |

**Additions or modifications to the 2010 CONSORT checklist. CONSORT = Consolidated Standards of Reporting Trials*
*†The items 5, 5a, 5b, 5c, 5d are consistent with the Template for Intervention Description and Replication (TIDieR) checklist*

***Cite as:*** *Boutron I, Altman DG, Moher D, Schulz KF, Ravaud P. CONSORT Statement for Randomized Trials of Nonpharmacologic Treatments: A 2017 Update and a CONSORT Extension for Nonpharmacologic Trial Abstracts. Annals of Internal Medicine. 2017 Jul 4;167(1):40–7.*

For peer review only - http://bmjopen.bmj.com/site/about/guidelines.xhtml

# Music to prevent delirium during neurosurgery (MUSYC): a single-centre prospective randomised controlled trial.



| | |
|---|---|
| Journal: | *BMJ Open* |
| Manuscript ID | bmjopen-2022-069957.R2 |
| Article Type: | Original research |
| Date Submitted by the Author: | 09-May-2023 |





| | |
|---|---|
| Complete List of Authors: | Kappen, Pablo; Erasmus Medical Center, Department of Neurosurgery; Erasmus Medical Center, Department of Neuroscience<br>Mos, M.I.; Erasmus Medical Center, Department of Neurosurgery; Erasmus Medical Center, Department of Neuroscience<br>Jeekel, Johannes; Erasmus Medical Center, Department of Neuroscience<br>Dirven, Clemens; Erasmus Medical Center, Department of Neurosurgery<br>Kushner, Steven; Erasmus Medical Center, Department of Psychiatry<br>Osse, Robert-Jan; Erasmus Medical Center, Department of Psychiatry<br>Coesmans, Michiel; Erasmus Medical Center, Department of Psychiatry<br>Poley, Marten; Erasmus Medical Center, Institute for Medical Technology Assessment (iMTA); Erasmus Medical Center, Department of Paediatric Surgery and Intensive Care<br>van Schie, Mathijs; Erasmus Medical Center, Department of Cardiology<br>van der Holt, Bronno; Erasmus Medical Center, Department of Haematology<br>Klimek, M; Erasmus Medical Center, Anesthesiology<br>Vincent, Arnaud; Erasmus Medical Center, Department of Neurosurgery |





| | |
|---|---|
| <b>Primary Subject Heading</b>: | Neurology |
| Secondary Subject Heading: | Anaesthesia, Surgery, Patient-centred medicine, Nursing |
| Keywords: | Neurosurgery < SURGERY, Delirium & cognitive disorders < PSYCHIATRY, COMPLEMENTARY MEDICINE, Clinical trials < THERAPEUTICS, PREVENTIVE MEDICINE |



SCHOLARONE™ Manuscripts

*I, the Submitting Author has the right to grant and does grant on behalf of all authors of the Work (as defined in the below author licence), an exclusive licence and/or a non-exclusive licence for contributions from authors who are: i) UK Crown employees; ii) where BMJ has agreed a CC-BY licence shall apply, and/or iii) in accordance with the terms applicable for US Federal Government officers or employees acting as part of their official duties; on a worldwide, perpetual, irrevocable, royalty-free basis to BMJ Publishing Group Ltd ("BMJ") its licensees and where the relevant Journal is co-owned by BMJ to the co-owners of the Journal, to publish the Work in this journal and any other BMJ products and to exploit all rights, as set out in our licence.*

*The Submitting Author accepts and understands that any supply made under these terms is made by BMJ to the Submitting Author unless you are acting as an employee on behalf of your employer or a postgraduate student of an affiliated institution which is paying any applicable article publishing charge ("APC") for Open Access articles. Where the Submitting Author wishes to make the Work available on an Open Access basis (and intends to pay the relevant APC), the terms of reuse of such Open Access shall be governed by a Creative Commons licence – details of these licences and which Creative Commons licence will apply to this Work are set out in our licence referred to above.*

*Other than as permitted in any relevant BMJ Author's Self Archiving Policies, I confirm this Work has not been accepted for publication elsewhere, is not being considered for publication elsewhere and does not duplicate material already published. I confirm all authors consent to publication of this Work and authorise the granting of this licence.*

For peer review only

# Music to prevent delirium during neurosurgery (MUSYC): a single-centre prospective randomised controlled trial.

P.R. Kappen[✉, 1, 2], M.I. Mos[1, 2], J. Jeekel[2], C.M.F. Dirven[1], S.A. Kushner[3], R.J. Osse[3], M. Coesmans[3], M.J. Poley[4, 5], M.S. van Schie[6], B. van der Holt[7], M. Klimek[8], A.J.P.E. Vincent[1]

**Affiliations.**

✉ Corresponding Author; Mr. Pablo Kappen, address: Doctor Molewaterplein 40, 3015 GD Rotterdam, the Netherlands, Room NA-2124, phone: +31(0)10 704 35 41, email: p.kappen@erasmusmc.nl

1. Department of Neurosurgery, Erasmus MC University Medical Center, Rotterdam, the Netherlands
2. Department of Neuroscience, Erasmus MC University Medical Center, Rotterdam, the Netherlands
3. Department of Psychiatry, Erasmus MC University Medical Center, Rotterdam, the Netherlands
4. Institute for Medical Technology Assessment (iMTA), Erasmus MC University Medical Center, Rotterdam, the Netherlands
5. Department of Paediatric Surgery and Intensive Care, Sophia Children's Hospital, Erasmus MC University Medical Center, Rotterdam, the Netherlands
6. Department of Cardiology, Erasmus MC University Medical Center, Rotterdam, the Netherlands
7. Department of Haematology, Erasmus Medical Center Cancer Institute, Rotterdam, the Netherlands
8. Department of Anaesthesiology, Erasmus Medical Center, Rotterdam, the Netherlands

**Abstract word count:** 293

**Manuscript word count:** 3717

**Reference count:** 41

**Number of figures:** 4

**Number of tables:** 3



For peer review only - http://bmjopen.bmj.com/site/about/guidelines.xhtml

For peer review only

## Abstract

### Objectives

Delirium is a serious complication following neurosurgical procedures. We hypothesize that the beneficial effect of music on a combination of delirium-eliciting factors might reduce delirium incidence following neurosurgery and subsequently improve clinical outcomes.

**Design** Prospective randomized controlled trial.

**Setting** Single centre, conducted at the neurosurgical department of the Erasmus MC Rotterdam, the Netherlands.

**Participants** Adult patients undergoing craniotomy were eligible.

**Interventions** Patients in the intervention group received preferred recorded music before, during and after the operation until day 3 after surgery. Patients in the control group were treated according to standard of clinical care.

**Primary and secondary outcome measures** Primary outcome was presence or absence of postoperative delirium within the first five postoperative days measured with the Delirium Observation Screening scale (DOSS) and, in case of a daily mean score of 3 or higher, a psychiatric evaluation with the DSM-5 criteria. Secondary outcomes included anxiety, heart rate variability (HRV), depth of anaesthesia, delirium severity and duration, post-operative complications, length of stay and location of discharge.

**Results** We enrolled 189 patients (music = 95, control = 94) from July 2020 through September 2021. Delirium, as assessed by the DOSS, was less common in the music (n = 11, 11.6%) than in the control group (n =21, 22.3%, OR:0.49, p=0.048). However, after DSM-5 confirmation, differences in delirium were not significant (4.2% vs. 7.4%, OR:0.47, p=0.342). Moreover, music increased the heart rate variability (RMSSD, p=0.012). All other secondary outcomes were not different between groups.

**Conclusion** Our results support the efficacy of music in reducing the incidence of delirium after craniotomy, as found with DOSS but not after DSM-5 confirmation, substantiated by the effect of music on pre-operative autonomic tone. Delirium screening tools should be validated and the long-term implications should be evaluated after craniotomy.




For peer review only - http://bmjopen.bmj.com/site/about/guidelines.xhtml

**Strengths and limitations**

- This is the first randomized controlled trial assessing the effect of music on delirium after craniotomy
- A variety of secondary outcomes, substantiating the onset of delirium and its clinical implications, were collected.
- Delirium was defined with the Delirium Observation Screening Score and the DSM-criteria.
- Due to the nature of the intervention, we did not blind the study, which could have influenced the outcome assessors.
- The generalizability of the results may be affected by the single-centre design of the study.



For peer review only

For peer review only - http://bmjopen.bmj.com/site/about/guidelines.xhtml

## Introduction

Delirium is defined in the latest Diagnostic and Statistical Manual of Mental Disorders (DSM-5) as “an acute disturbance in attention and cognition which is not better explained by another neurocognitive disorder such as for example dementia”. To increase the recognition of delirium during hospital stay, a variety of delirium diagnostic screening tools have been developed, which can also be assessed by other healthcare workers. Delirium in neurosurgical patients has been reported in 4 to 44% of cases, with a large variation in definition and assessment methods. (1) The high incidence in this population is probably caused by the underlying massive neuro-inflammation which is usually induced during intracranial procedures. (2) Delirium, also in neurosurgical literature, is often multifactorial in aetiology and can be influenced by a number of predisposing (e.g. older age, cognitive impairment, multiple comorbidities) and precipitating factors (e.g. infections, operations, drugs).(3-8) The clinical relevance of delirium in neurosurgery remains difficult to assess objectively, as criteria for delirium overlap with symptoms from the primary neurologic injury. However, delirium independently predicted clinical outcomes in neurosurgical and neurocritically ill patients such as worse functional outcome (9), length of stay, costs and death. (10) These complications justify the search for preventive therapies for post-operative delirium in neurosurgical patients.

Although promising preventive approaches are emerging, pharmacological treatments have inconsistent results and are accompanied with side effects. (11, 12) Non-pharmacological multi-component approaches for primary prevention, such as reorientation, early mobilization, therapeutic activities, hydration, nutrition and sleep strategies have been shown to be effective and cost-reducing in other patient groups. However, these approaches can be labour intensive, and include the use of volunteers, or non-licensed professionals to enhance feasibility. (13)

Recorded music is an easy applicable intervention which neatly fits throughout the entire perioperative process and has been shown to be effective in the surgical population in reducing a combination of delirium-eliciting factors such as pre-operative anxiety, post-operative pain, stress response and opioid/sedation requirement.(14-21) A recent meta-



For peer review only - http://bmjopen.bmj.com/site/about/guidelines.xhtml

analysis, evaluating six randomised pilot studies, found music potentially being effective in preventing postoperative delirium in postsurgical patients. However, these studies did not include neurosurgical patients. (22)

We therefore designed a randomized controlled trial to assess the effect of music in the prevention of post-operative delirium in neurosurgical patients.

For peer review only

## Methods

*Patient and public involvement*

Patients were involved in the composition of the music playlists, as these were based on their music preference, the role music plays in their life (ie, whether they are musician/ just listen to music) and the importance of music. The results of our trial were disseminated to the participating patients through a letter after publication.

*Study design*

The Music to prevent deliriUm during neuroSurgerY Clinical (MUSYC) trial was a single centre, prospective randomized controlled trial conducted at the Erasmus MC Rotterdam, the Netherlands. The trial compared effects of music administered before, during and after craniotomy with standard of clinical care. The Medical Ethics Review Committee of the Erasmus Medical Center, Rotterdam (Erasmus MC), declared this study not subject to the Medical Research Involving Human Subjects Act (i.e. 'Non-WMO'), and approved this study (MEC-2020-0064), on the 25$^{th}$ of February of 2020, as such.

The trial protocol was designed by neurosurgeons, psychiatrists, anaesthesiologists and neuroscientists and followed the Standard Protocol Items: Recommendations for Interventional Trials (SPIRIT) guidelines and the Consolidated Standards of Reporting Trials (CONSORT) guidelines for non-pharmacological treatments (see checklist in online supplementary material). The trial was registered (trialregister.nl: NL8503 and ClinicalTrials.gov: NCT04649450) and details of the protocol have been published previously. (23, 24)

We expected an incidence of delirium in our control group of 30%, which was based on the incidence of 24.2–32.4% documented in neurosurgical literature using the same screening tool (i.e. DOSS).(5, 7) When designing the trial, the expected effect could not be based on previous literature as no pooled effect of music on delirium was reported. Other non-pharmacological interventions mentioned a relative reduction of 36%–77% and we therefore considered the intervention clinically relevant if a relative reduction of 60%, corresponding to an absolute reduction of 18%, was achieved.(25, 26) Assuming a loss to follow-up of 5%, we

For peer review only

estimated that a target sample size of 189 patients would provide the trial with a power of 80%.

From July 2020 through September 2021: 189 patients were registered and randomly assigned to a trial group: 95 in the music group and 94 in the standard care group (Fig. 1). Randomization was done in a 1:1 ratio, by a secured online software program (ALEA; FormsVision BV, Abcoude, The Netherlands) and stratified per type of disease characteristic (i.e. 'neuro-oncology', 'neurovascular', 'traumatic brain injury', 'infectious') and age (i.e. 'younger than 60 years', '60 years or older'). Variable block sizes were used in which in each block both groups were represented equally.

*Patients*

Adult patients (i.e. age 18 years or more) undergoing craniotomy (i.e. opening the dura requiring bone flap removal) at the Erasmus MC with sufficient knowledge of the Dutch language were eligible for study participation. Exclusion criteria were: impaired awareness before surgery (i.e. motoric less than 6 in the Glasgow Coma Scale), planned post-operative intensive care unit (ICU) admission, suspected delirium (defined as fluctuating awareness) at baseline, antipsychotic treatment, undergoing surgery impeding supply of music (i.e. awake craniotomy or vestibular schwannoma surgery), bilateral hearing impairment and participation in other clinical trials interfering with results. During inclusion, one participant reported that music induced epileptic seizures (known as musicogenic epilepsy): this patient was excluded (and the exclusion criteria were adopted accordingly), as it was considered unethical to expose such a patient to music. Eligible patients were approached and written informed consent by patient or legal representative was obtained.

*Intervention*

All participating subjects were treated according to standard of care. Method of music intervention administration (i.e. type, frequency and duration) was applied based on previous studies.(17, 18) Participants in the intervention group (i.e. music group) received an over-ear headphone and a tablet with access to a platform with different pre-selected music playlists (i.e. jazz, blues, classic, electronic, pop, 60s, 70s, 80s etc), in which the music selection could be extended based on patients' wishes. These patients received the first 30 minutes of music at the pre-operative holding area the day of operation (day 0) while awaiting surgery (see



For peer review only - http://bmjopen.bmj.com/site/about/guidelines.xhtml

supplementary figure 1 for 'Study Course MUSYC trial') which was stopped before reaching the operation room. In the operating room, in-ear earphones after intubation, which were compatible with the Mayfield clamp and site of operation, were inserted and music was continued until just before detubation. After surgery, during recovery at the post-operative care unit (PACU), another 30 minutes of recorded music through over-ear headphones was administered. Finally, participants received 30 minutes of recorded music twice a day until post-operative day 3. Patients in the control group were asked to refrain from music listening, however this was not strictly controlled as this would influence the standard of clinical care too much. Nurses were instructed to monitor for music listening behaviour with a diary which was placed next to the music equipment. Periodically (approximately every six months) a training was given for all nurses on the ward to explain how music had to be administered and monitored.

*Outcome measures*

The primary outcome measure was presence or absence of postoperative delirium within the first five postoperative days. (27) The diagnosis of delirium required a two-step procedure; first, all participating patients were daily screened by the treating nurse using the Delirium Observation Screening scale (DOSS), a validated 13-item delirium screening tool with higher scores indicating a higher probability of delirium. Use of the DOSS was already current practice at our department and was administered by the nurse during each shift (three- 8-hour shifts per day).(28-31) Second, in case of a daily mean score of 3 or higher, which was radiologically not substantiated by a neurosurgical complication, a psychiatrist was consulted to assess the clinical diagnosis of delirium based on the DSM-5 criteria.(32) DSM-5 criteria assessment was conducted once in case of an increased mean DOS score of 3 or above. This was not standardized for a certain moment of the day, but depended on the timing of the increased mean DOSS score and the logistic of the consulting psychiatrist that specific day. We chose not to blind the assessors from the intervention, as this could not be secured which might have led to misleading results.

Secondary outcomes were assessed to substantiate the effects of music on delirium and its clinical implications. Pre-operative secondary outcomes (during the 30-minute pre-operative holding stay) included anxiety (measured with the Visual Analogue Scale-anxiety/VAS-A) and heart rate variability (HRV), a marker of the autonomic tone reflecting parasympathetic



For peer review only - http://bmjopen.bmj.com/site/about/guidelines.xhtml

nervous activity, measured with a 30 minutes electrocardiography (ECG) recording. The following HRV parameters were analysed: standard deviation of normal sinus beats (SDNN), root mean square of successive differences between normal heartbeats (RMSSD), the number of adjacent NN intervals that differ from each other by more than 50 ms (NN50) and the ratio of low frequency to high frequency power (HF/LF). Intra-operative secondary outcomes included depth of anaesthesia with Bispectral Index (BIS, Aspect ™ version 3.22) with standardized sedation dosages (propofol and remifentanil). BIS was measured from the non-operated side, if feasible with site of resection, and the anaesthesiologist was blinded from the intra-operative BIS values, which was considered ethical as this form of monitoring is not standard of clinical care during intracranial procedures. Post-operative secondary outcomes (measured during the entire post-operative stay) were delirium severity (using the Delirium Rating Scale-revised-98/DRS-R-98) and delirium duration (onset until first day DOSS score <3), pain (Numeric Rating Scale/NRS pain and dosages analgesic), post-operative complications, length of stay and location of discharge. Finally, patients' satisfaction of receiving music was assessed with a 100- point visual analogue scale (administered at the outpatient clinic 6 weeks after discharge).

Baseline characteristics were extracted at baseline from questionnaires or the electronic patient file consisting of age, gender, medical history, daily function (Karnofsky Performance Scale/KPS or Modified Rankin scale/MRS), quality of life (100-likert scale, EQ-5D and EORTC QLQ – BN-20), cognitive function (Montreal Cognitive assessment / MoCA), disease characteristics (i.e. neurologic deficit, type and side of intracranial pathology) and operation details (i.e. emergency grade, duration of surgery).

*Statistical analysis*

The main analysis was the comparison of the proportion of patients with delirium between the two arms in the Intention-to-Treat population (ITT; all registered and randomized patients) using univariate logistic regression. As sensitivity analyses the proportion of patients with delirium was also compared between the two arms in the modified ITT (m-ITT, i.e. ITT but excluding patients who were found to be ineligible after randomization) and Safety Population (i.e. all patients who underwent craniotomy). A multivariable logistic regression analysis with the stratification factors in the ITT population was also performed as sensitivity analysis, while multivariable analyses in the m-ITT and safety population should



For peer review only - http://bmjopen.bmj.com/site/about/guidelines.xhtml

be considered as descriptive and therefore as hypothesis-generating only. All secondary outcomes were analyses in the mITT population, and should only be considered as descriptive only.

A 2-hour recording of BIS (blinded from the anaesthesiologist between operation minute 60 to 180) was split into samples of 15 minutes as time points. A 30-minute recording of HRV was split into samples of 5 minutes as time points. Subsequently, we ran a linear mixed model with unstructured covariance for BIS and HRV, as a within subject variability was suspected, with time point and interaction group/time point as independent variables, as presented with fixed effects (beta/$\beta$1) and confidence intervals (95%CI). Moreover, a sensitivity analyses was conducted with possible additional confounding for depth of anaesthesia (BIS level) including age, comorbidity, type of disease, American Society of Anesthesiologists (ASA) classification and steroid use. The residual plots were visually observed and a log transformation was applied in case of heteroscedasticity.

A two-sided p value of 0.05 or less was considered statistically significant. All statistical analysis were conducted using R (version 4.1.1).



For peer review only - http://bmjopen.bmj.com/site/about/guidelines.xhtml

## Results

A total of 309 patients were expected to be eligible after screening, of which 189 patients were registered and randomly assigned to the music (n=95) or control group (n=94). Five patients (4 in the music group and 1 in the control group) were excluded after registration due to withdrawing consent (n = 1), pre-operative use of antipsychotic treatment (n=1), or no craniotomy (n = 3, one operation cancelled, one burr-hole-biopsy and one no necessity of opening the dura). The remaining 184 patients, constituting the modified intention-to-treat population, were followed up for all the secondary outcomes.

The baseline characteristics were similar in the two trial groups (ITT population), with a median age of 60 years and 44% being female (table 1). Psychiatric medical history was reported in 11%, including depression (n = 10) in most cases, pre-operative usage of possible delirium eliciting medication (i.e. antidepressants and sleep medication) in 15% and no dementia in our cohort. Baseline cognition (MoCA) was 24/20-27 (median/IQR), quality of life (QoL) was 70/55-80 (median/IQR) and no neurologic symptoms (in case of a KPS = 100 or MRS = 0) at admission were present in 23%. This cohort included mostly neuro-oncological patients (n = 161, 85%), with neurological deficit present in 38% and frontal localisation in 34%. Affection for music was reported with an importance of 8/7-8 (median/IQR), only 5% reported never to listen music in daily life. Surgical details showed duration of surgery of 220 minutes and emergency surgery (i.e. within 72 hours) in only 3%.

*Primary outcome*

In the music group, adherence to the music intervention before, during, and directly after surgery was 96%, 100%, and 74%, respectively (supplementary table 1). The following days the adherence decreased each day, from 70% on the first morning to 47% at noon on day 3. The total listening time was a median of 130 minutes (IQR, 73-230) during the five days of admission or until discharge.

A high DOSS score (i.e. 3 or higher) was observed in 32 patients. This was caused by a neurosurgical complication, as confirmed on radiology, in three patients; two patients with infarction after a vascular procedure with hemiparesis and decreased attention. The other patient had a subdural hematoma which was evacuated in the operation room. This resulted in 29 patients with possible delirium by DOSS; 21 were evaluated by the psychiatrist who



For peer review only - http://bmjopen.bmj.com/site/about/guidelines.xhtml

diagnosed delirium in 11 patients (57%, supplementary figure 2) based on the DSM-5 criteria.

According to the DOSS, a significantly higher incidence of delirium was observed in the control (n=21) vs. music (n=11) group in the ITT population for the univariable (22.3 vs. 11.6%, p =0.048) and multivariable (OR/95%CI: 0.49/0.20-1.00, p =0.050) analysis. This was not observed in the mITT (p = 0.064) and the SP (p =0.064) population. The occurrence of a DSM-5 confirmed delirium, was not statistically significant between the control (n=7) vs. music (n=4) group in the ITT (7.4 vs 4.2%, OR = 0.55), the mITT (7.5 vs. 4.4%, OR = 0.57), and the SP (7.4 vs. 4.3%, OR = 0.58) population (table 2).

Of those patients with DSM-5-confirmed delirium (n = 11); severity of delirium (mean/SD) was 12.60/5.52, which was not different between the two arms (p =0.857). The duration of delirium (days, mean/SD) was 3.36/4.69, which was not different between the two arms (p = 0.761).

*Secondary outcome*

Available ECG data (n=87) revealed that heart rate remained constant in the music group while it decreased after 15 ($\beta_1$ = 2.89, p =0.043), 25 ($\beta_1$ = 3.36, p=0.05) and 30 ($\beta_1$ = 5.06, p=0.011) minutes in the control group (table 3 and figure 2). A significant increase on HRV was found by music at 5 minutes on RMSSD ($\beta_1$ = 55.08, p = 0.012). No significant effect was found on the other HRV parameters. Available depth of anaesthesia (n=70) data revealed no significant difference between the music and control group at the several analysed time points (figure 3). A trend towards less anxiety in the music group was observed (p=0.058, figure 4). All other secondary outcomes were not different between groups.

Patient satisfaction (median/IQR) in the music group who filled in the questionnaire (n = 68), was 85/80-95, and 88% reported they would want to receive music in case of future surgery.



For peer review only - http://bmjopen.bmj.com/site/about/guidelines.xhtml

## Discussion

We found a significant decrease on the incidence of post-operative delirium by the addition of music perioperatively using the DOSS, however this was not significant when assessed by the DSM-5 criteria. Second, music activated pre-operative heart rate variability, a marker of autonomic tone. Last, no significant effect on anxiety, depth of anesthesia, post-operative complications, length of stay and location of discharge were found. Clinical implications and limitations are discussed below.

We found a significant decrease on the incidence of post-operative delirium, defined with DOSS, by the addition of perioperative music. A recent published systematic review conducted a meta-analysis on the preventive effect of music on delirium with six studies and found a relative reduction similar to ours (0.52 vs 0.48)(22) This meta-analysis included pilot studies which administered patient preferred music similar to our design, but varied from ours as the population considered surgical and non-surgical ICU patients and delirium was defined with the NEECHAM, CAM-ICU or own definitions. Our sample size calculation was based on other neurosurgical studies evaluating delirium in case of increased scores on delirium screening tools. (1) When handling screening tools for delirium definition by using the DOSS, we support the efficacy of music in lowering the incidence of post-operative delirium. However, although a similar trend was found, significance of results was not achieved when assessed by the DSM-5 criteria. The discrepancy between DOSS and DSM-5 may have several explanations. First, DSM-5 was evaluated by a psychiatrist *after* an increased DOSS score. Hence, delirium may have been resolved over time before the psychiatrist its assessment.  Moreover, DOSS evaluation was conducted three times per day by the nurses, as opposed to DSM-5 determination which was only evaluated once. DSM-5 assessment during day-time might have missed some cases as delirium fluctuates over the course of the day, especially for the delirium-type present during night-time. Also, not all our patients with increased DOSS were evaluated by a psychiatrist due to logistics and we might have missed some patients with delirium. Second, delirium screening tools have not been validated within the neurosurgical population. (5-8, 33-37) Hence, while high diagnostic accuracies in the general population justify diagnostic usage of delirium screening tools, it is unclear whether this can be adopted to our complicated patient population, as a positive screen for delirium



For peer review only - http://bmjopen.bmj.com/site/about/guidelines.xhtml

may be due to the underlying neurological disease or its sequelae (e.g., oedema, vasospasm, seizures, rebleeding, ischemia) leading to false-positive results.

We propose a vagal-mediated anti-inflammatory response as a candidate pathway of music on delirium, as hypotheses of delirium rely on neuro-inflammatory reactions within the brain. (2) Although we did not assess inflammatory cytokines in our study, vagal nerve activation by music was supported by the increased HRV, revealed by an increased RMSSD on ECG during the pre-operative music session. The activation of HRV by music in brain-damaged patients was proven earlier and is considered a valid marker of parasympathetic nervous activation. (38, 39) Whether HRV could be used as a marker for postoperative recovery, remains to be determined. Moreover, we observed a decreasing trend in pre-operative anxiety by music, although this did not reach significance. This pre-operative parasympathetic activation and anxiety reduction may have induced a sedative-sparing effect, subsequently increasing cortical engagement and cognitive processing.(40) We did not find a deeper level of anesthesia in the music group. Literature is contradictory on the correlation of depth of sedation and anesthetic requirements as music listening is associated with decrease of depth of anesthesia, but no decrease of sevoflurane was achieved when pursuing constant depth of anesthesia. Future neurosurgical studies should confirm whether concentration of sedation can be reduced with music in case of standardized depth of anesthesia (BIS) levels.

We found high adherence to the music intervention before surgery. High importance of music in daily life, the number of hours listening to music in daily life and the willingness to receive music intervention in case of future surgeries was found in our cohort, which are considered important facilitators for music implementation. (41) However the adherence declined after surgery, due to pain, nausea, logistics (i.e. for MRI) or unwillingness. Absence of a near future operation prospect may have reduced the urgent necessity of music, resulting in the post-operative decline in adherence. Lack of the knowledge of the intervention is considered a barrier for implementation. Informing patients, substantiated by the results from efficacy studies such as this trial, may aid in the implementation of music in the neurosurgical population.



For peer review only - http://bmjopen.bmj.com/site/about/guidelines.xhtml

Although delirium is (most often) temporary and self-limiting, delirium independently predicts clinically relevant outcomes in neurologically damaged patients. (9) (10) Although a trend was observed, we did not find any significant positive effects on complications, length of admission or location of discharge. Future studies should assess the long-term implications of delirium defined with either DOSS and DSM-criteria after discharge in neurosurgical patients.

*Strengths and limitations*

This is the first randomized controlled trial assessing the effect of music on delirium after craniotomy and the largest assessing the effect of music against delirium. We showed that music reduced the incidence of delirium when defined with the DOSS but not after DSM-5 confirmation. Our study was subject to several limitations: first, not all our patients with increased DOSS were evaluated by a psychiatrist due to logistics and we might have missed some patients with delirium. However, we feel that this did not affect our conclusions, because, with our low confirmation rate of suspected delirium, the study would still have been underpowered. Second, due to the nature of the intervention we did not blind the study, which could have influenced the outcome assessors. However, blinding could not be secured which might have led to misleading results. Third, the generalizability of the results may be affected by the single-centre design of the study.

For peer review only

## Conclusion

Our results support the efficacy of music in reducing the incidence of delirium after craniotomy, as found with DOSS but not after DSM-5 confirmation. Delirium screening tools should be validated within the neurosurgical context and the long-term implications of a delirium, either defined by an increased DOSS or DSM-5, should be evaluated. This effect of music is substantiated by the effect of music on an increased pre-operative HRV. Last, although pre-operative adherence was high, this declined after surgery which should be taken into account, when considering implementation in the neurosurgical population.

## Contributors

Each author has contributed significantly to, and is willing to take public responsibility for, one or more aspects of the study. AJPEV, CMFD, JJ, MK and PRK conceived the study idea. PRK coordinated the research protocol. PRK and MIM extracted the data. PRK, BvdH and AJPEV conducted the statistical analysis and wrote the first draft of the manuscript. JJ, MIM, CMFD, MK, SK, RJO, MC, MJP, MS and AJPEV critically revised the manuscript. All authors have seen and approved the final version of the manuscript being submitted. The article is the authors' original work, has not received prior publication and is not under consideration for publication elsewhere.

## Funding

This research is funded with the Erasmus MC Efficiency Grant (Grant number: 19105) by the Erasmus Medical Centre, Rotterdam, The Netherlands.

## Competing interests

None declared.

## Research ethical approval

This study involves human participants and was approved by the Medical Ethics Review Committee of the Erasmus Medical Center, Rotterdam (Erasmus MC), which declared this study not subject to the Medical Research Involving Human Subjects Act (i.e. 'Non-WMO'), and approved this study (MEC-2020-0064), on the 25th of February of 2020, as such. Written informed consent was obtained for each inclusion by patient or legal representative



For peer review only - http://bmjopen.bmj.com/site/about/guidelines.xhtml

**Data Sharing Statement**

The raw data supporting the conclusions of this article will be made available by the authors, without undue reservation.

For peer review only



For peer review only - http://bmjopen.bmj.com/site/about/guidelines.xhtml

**Table 1. Baseline characteristics**

| | Control (n=94) | Music (n = 95) |
|---|---|---|
| **Pre-hospital demographics** | | |
| Age (years) * | 61 (51-69) | 60 (49-69) |
| Sex (% female) | 46 (49%) | 38 (40%) |
| Medical history (n/%) | | |
| Somatic history[1] | 79 (84%) | 74 (78%) |
| Psychiatric history | 14 (15%) | 6 (6%) |
| Delirium prior admission | 2 (2%) | 5 (3%) |
| Dementia | 0 (0%) | 0 (0%) |
| Body mass index (kg/m$^2$) | 26 (24-30) | 26 (23-28) |
| Medication (n/%)[2] | 15 (26%) | 14 (15%) |
| Intoxication[3] | | |
| Abuse of alcohol (n/%) | 6 (6%) | 1 (1%) |
| Abuse of drugs (n/%) | 3 (3%) | 3 (3%) |
| **In-hospital demographics** | | |
| Pre-hospital functional status[4] | 80 (70-90) | 80 (70-90) |
| KPS (100-0) | 70 (18-80) | 70 (45-80) |
| MRS (5-1) | 1.0 (0.25-2.0) | 1.0 (1.0-2.0) |
| Quality of Life (1- 100) | 70 (60-79) | 70 (55-80) |
| Cognitive function (0 - 30)[5] | 24 (19-27) | 25 (21-27) |
| Electrolyte disturbance (n/%)[6] | 10 (11%) | 9 (10%) |
| | | |
| **Disease characteristics** | | |
| Neurologic deficit (n/%) | 35 (37%) | 36 (38%) |
| Type | | |
| Oncologic | 81 (86%) | 80 (84%) |
| Vascular | 12 (13%) | 14 (15%) |
| Other | 1 (1%) | 1 (1%) |
| Frontal disease localization (n/%) | 40 (43%) | 24 (25%) |
| **Music affection** | | |
| Music importance | 7.0 (6.8-8.0) | 8.0 (7.0-8.0) |
| Frequency listening | | |
| The whole day | 29 (31%) | 21 (22%) |
| Some hours per day | 44 (47%) | 54 (57%) |
| Some hours per week | 9 (10%) | 12 (13%) |
| Never | 6 (6%) | 3 (3%) |
| Played an instrument | 20 (21%) | 20 (21%) |
| | | |
| **Operation details[7]** | | |
| Operation duration (minutes) | 220 (160-320) | 210 (140-290) |
| Emergency operation (n/%)[8] | 2 (2%) | 4 (4%) |
| Supine position (n/%) | 81 (86%) | 80 (84%) |
| Tramrail sign tension (n/%) | 68 (72%) | 67 (7%) |

*All continuous data are presented in median/IQR. 1. Somatic history: including systematic disease (DM, hypertension) currently treated by medication and prior surgery (requiring general anesthesia on the operation room). 2. Medication known to induce delirium before admission, such as sleep medication, morphine, atropine and antidepressants 3. Reported abusive use of alcohol and/or drugs. 4. Patients' functional performance with the Karnofsky Performance Scale (ranging from 100/'No complaints' to 0/'Death') and Modified Ranking Scale (ranging from 0/'No symptoms to 5/'Death'. 5. Cognitive function assessed with the Montreal Cognitive Assessment / MoCA 6. Electrolyte disturbance (mEq/L) in case of sodium >145 or <135 or potassium <3.5 or >5. 7. Patients in the MITT population (n=184) only. 8. Operation indication within 72 hours.

For peer review only



For peer review only - http://bmjopen.bmj.com/site/about/guidelines.xhtml

1

2 **Table 2. Primary outcome**

| | Control (n/%) | Music (n/%) | Univariable analysis (OR/95%CI)* | P value[1] | Multivariable analysis (OR/95%CI)* | P value[2] |
|---|---|---|---|---|---|---|
| **Intention-to-treat analysis (ITT)** | | | | | | |
| Increased DOSS | 21/22.3% | 11/11.6% | 0.46/0.19;1.00 | 0.048 | 0.49/0.20;1.00 | 0.050 |
| Confirmed by DSM-5 | 7/7.4% | 4 /4.2% | 0.55/0.14;1.96 | 0.342 | 0.57/0.16;2.07 | 0.39 |
| **Modified Intention-to-treat analysis (mITT)** | | | | | | |
| Increased DOSS | 21/22.6% | 11/12.1% | 0.47/0.21;1.04 | 0.060 | 0.47/0.16;2.07 | 0.064 |
| Confirmed by DSM-5 | 7/7.5% | 4/4.4% | 0.57/0.14;2.03 | 0.370 | 0.58/0.16;2.10 | 0.412 |
| **Safety Population (SP)** | | | | | | |
| Increased DOSS | 21/22.3% | 11/12.0% | 0.47/0.21;1.04 | 0.061 | 0.47/0.21;1.04 | 0.064 |
| Confirmed by DSM-5 | 7/7.4% | 4/4.3% | 0.58/0.14;2.03 | 0.370 | 0.58/0.16;2.11 | 0.414 |

*Odds Ratio/95%Confidence interval (OR/95%CI). 1. P values assessed with the Chi-squared test 2. Logistic regression analysis with groups, type disease and gender as independent variables.

3

4

5

6

7

8

9

10

11

12

13

14

15

16

17

18

19

20

21



For peer review only - http://bmjopen.bmj.com/site/about/guidelines.xhtml

**Table 3. Secondary outcomes**

| | Control (n = 93) | Music (n = 91) | P value |
|---|---|---|---|
| **Univariable analyses** | | | |
| Anxiety difference (mean/SD)[1] | 0.05/0.94 | -0.25/1.49 | 0.058 |
| Pain (mean/SD)[2] | 3.56/1.91 | 3.16/1.74 | 0.246 |
| Naproxen mg (mean/SD) | 13.6/75.4 | 2.75/26.2 | 0.103 |
| Oxycodon mg (mean/SD) | 2.03/4.35 | 1.61/3.31 | 0.828 |
| No complications (n/%) | 25 (26.9%) | 21 (23.1%) | 0.551 |
| Length of stay, days (mean/SD) | 7.43 (8.08) | 6.74 (8.26) | 0.947 |
| Discharge home (n/%) | 77 (82.8%) | 76 (83.5%) | 0.896 |

**Multivariable analyses**

| Heart Rate Variability/HRV ($\beta_1$/95%CI)[4] | | | | |
|---|---|---|---|---|
| Time point[3] | SDNN | RMSSD | NN50 | LF/HF |
| 5 minutes | 38.84/ -2.23;79.91 | 55.08/13.16;97.00* | 17.64/ -3.92;39.21 | -0.46/-1.04;0.11 |
| 10 minutes | 18.87/ -18.23;55.97 | 18.79/ -16.16;53.75 | 11.11/ -8.67;30.88 | 0.49/ -0.18;1.17 |
| 15 minutes | 2.49/ -35.41;40.38 | -3.20/ -38.60;32.19 | 9.14/ -11.11;29.38 | 0.34/ -0.28;0.96 |
| 20 minutes | -11.65/ -52.87;29.58 | 3.12/ -40.54;46.78 | 8.22/ -12.00;28.45 | 0.56/ -0.07;1.19 |
| 25 minutes | -15.62/-57.24;26.00 | -11.85/ -58.03;34.33 | -3.56/ -27.10;19.98 | 0.45/-0.26;1.16 |
| 30 minutes | 7.41/-38.04;52.87 | 22.20/ -17.01;61.41 | -5.98/ -28.49;16.53 | 0.46/-0.18;1.09 |

| Time point[3] | Depth of anesthesia/BIS ($\beta_1$/95%CI)[5] | P value |
|---|---|---|
| 15 minutes | 0.71/ -3.17;4.58 | 0.717 |
| 30 minutes | -1.44/ -3.34;0.46 | 0.139 |
| 45 minutes | -1.06/ -3.42;1.30 | 0.378 |
| 60 minutes | -2.23/ -5.34;0.89 | 0.162 |
| 75 minutes | -1.82/ -5.48;1.83 | 0.328 |
| 90 minutes | -2.46/ -6.72;1.79 | 0.256 |
| 105 minutes | -0.50/ -6.17;5.16 | 0.862 |
| 120 minutes | 0.31/ -5.44;6.05 | 0.917 |

Secondary outcomes analyzed on the mITT population. 1. Anxiety differences between first and second measures with VAS-A. 2. Pain (NRS) over the first three post-operative days. 3. Time-points samples included in the linear mixed model. 4. HRV analyses; 30 minute of pre-operative ECG recordings split into 5-minute samples, all values marked with * are significant (i.e. p<0.05). 5. BIS analyses: 120 minute of intra-operative BIS registration split into 15-minute samples.

For peer review only



For peer review only - http://bmjopen.bmj.com/site/about/guidelines.xhtml

**Figure 1.** CONSORT Trial flowchart.

**Figure 2.** Pre-operative course of HRV. A 30-minute recording of HRV was split into samples of 5 minutes as time points and compared between groups. Heart rate remained constant in the music group while it decreased after 15 ($\beta_1$ = 2.89, p =0.043), 25 ($\beta_1$ = 3.36, p=0.05) and 30 ($\beta_1$ = 5.06, p=0.011) minutes in the control group. A significant increase on HRV was found by music at 5 minutes on RMSSD ($\beta_1$ = 55.08, p = 0.012). No significant effect was found on the other HRV parameters.

**Figure 3**. Intra-operative depth of anaesthesia. A 2-hour recording of BIS was split into samples of 15 minutes as time points and compared between groups. Available depth of anaesthesia (n=70) data revealed no significant difference between the music and control group at the several analysed time points (figure 3).

**Figure 4.** Two anxiety measurements (VAS-A) were conducted before surgery and categorized in 'No anxiety' (0 – 2), 'Medium anxiety (3-6) and 'High anxiety (7 – 10). Most patients remained in their anxiety level, although some patients showed a decrease pre-operative anxiety when receiving music, but this difference was non-significant between groups (p=0.058).

**Supplementary figure 1. Study course MUSYC trial.**

**Supplementary figure 2. Course delirium diagnosis.**

**Supplementary table 1. Adherence to music intervention during trial.**



For peer review only - http://bmjopen.bmj.com/site/about/guidelines.xhtml

## References


1. Kappen PR, Kakar E, Dirven CMF, van der Jagt M, Klimek M, Osse RJ, et al. Delirium in neurosurgery: a systematic review and meta-analysis. Neurosurg Rev. 2021;45(1):329-341.
2. Maldonado JR. Neuropathogenesis of delirium: review of current etiologic theories and common pathways. Am J Geriatr Psychiatry. 2013;21(12):1190-222.
3. Oh ES, Fong TG, Hshieh TT, Inouye SK. Delirium in Older Persons: Advances in Diagnosis and Treatment. JAMA. 2017;318(12):1161-74.
4. Flanigan PM, Jahangiri A, Weinstein D, Dayani F, Chandra A, Kanungo I, et al. Postoperative Delirium in Glioblastoma Patients: Risk Factors and Prognostic Implications. Neurosurgery. 2018;83(6):1161-72.
5. He Z, Cheng H, Wu H, Sun G, Yuan J. Risk factors for postoperative delirium in patients undergoing microvascular decompression. PLoS One. 2019;14(4):e0215374.
6. Wang CM, Huang HW, Wang YM, He X, Sun XM, Zhou YM, et al. Incidence and risk factors of postoperative delirium in patients admitted to the ICU after elective intracranial surgery: A prospective cohort study. Eur J Anaesthesiol. 2020;37(1):14-24.
7. Zipser Z, Deuel J, Ernst J, Schubert M, von Kanel R, Bottger S. The predisposing and precipitating risk factors for delirium in neurosurgery: a prospective cohort study of 949 patients. Acta Neurochir (Wien). 2019;161(7):1307-15.
8. Kappen PR, Kappen HJ, Dirven CMF, Klimek M, Jeekel J, Andrinopoulou ER, et al. Postoperative Delirium After Intracranial Surgery: A Retrospective Cohort Study. World Neurosurgery. 2023;(22)01838-1.
9. Budėnas A, Tamašauskas Š, Šliaužys A, Navickaitė I, Sidaraitė M, Pranckevičienė A, et al. Incidence and clinical significance of postoperative delirium after brain tumor surgery. Acta Neurochir. 2018;160(12):2327-37.
10. Chen H, Jiang H, Chen B, Fan L, Shi W, Jin Y, et al. The Incidence and Predictors of Postoperative Delirium After Brain Tumor Resection in Adults: A Cross-Sectional Survey. World Neurosurg. 2020;140:e129-e39.
11. Liu Y, Li XJ, Liang Y, Kang Y. Pharmacological Prevention of Postoperative Delirium: A Systematic Review and Meta-Analysis of Randomized Controlled Trials. Evid Based Complement Alternat Med. 2019;2019:9607129.
12. Vlisides P, Avidan M. Recent Advances in Preventing and Managing Postoperative Delirium. F1000Res. 2019;8.
13. Inouye SK, Westendorp RG, Saczynski JS. Delirium in elderly people. Lancet. 2014;383(9920):911-22.
14. Cerejeira J, Batista P, Nogueira V, Vaz-Serra A, Mukaetova-Ladinska EB. The stress response to surgery and postoperative delirium: evidence of hypothalamic-pituitary-adrenal axis hyperresponsiveness and decreased suppression of the GH/IGF-1 Axis. J Geriatr Psychiatry Neurol. 2013;26(3):185-94.
15. Clegg A YJ. Which medications to avoid in people at risk of delirium: a systematic review. 2011(40:23–29).
16. Fu VX, Oomens P, Sneiders D, van den Berg SAA, Feelders RA, Wijnhoven BPL, et al. The Effect of Perioperative Music on the Stress Response to Surgery: A Meta-analysis. J Surg Res. 2019;244:444-55.
17. Fu VX OP, Klimek M, Verhofstad MHJ, Jeekel J. The effect of perioperative music on medication requirement and hospital length of stay: a meta-analysis. Annals of Surgery. July 2019;272(6):961 - 72.
18. Kuhlmann AYR, de Rooij A, Kroese LF, van Dijk M, Hunink MGM, Jeekel J. Meta-analysis evaluating music interventions for anxiety and pain in surgery. Br J Surg. 2018;105(7):773-83.
19. Lee JH. The Effects of Music on Pain: A Meta-Analysis. J Music Ther. 2016;53(4):430-77.
20. Leung JM SL, Paul S. Does postoperative delirium limit the use of patient-controlled analgesia in older surgical patients? 111:625–631. 2009.




For peer review only - http://bmjopen.bmj.com/site/about/guidelines.xhtml

21. Hole J, Hirsch M, Ball E, Meads C. Music as an aid for postoperative recovery in adults: a systematic review and meta-analysis. Lancet. 2015;386(10004):1659-71.
22. Golubovic J, Neerland BE, Aune D, Baker FA. Music Interventions and Delirium in Adults: A Systematic Literature Review and Meta-Analysis. Brain Sci. 2022;12(5).
23. Kappen P, Jeekel J, Dirven CMF, Klimek M, Kushner S, Osse RJ, et al. Music to prevent deliriUm during neuroSurgerY (MUSYC) Clinical trial: a study protocol for a randomised controlled trial. BMJ Open. 2021;11(10):e048270.
24. Chan AW, Tetzlaff JM, Altman DG, Laupacis A, Gotzsche PC, Krleza-Jeric K, et al. SPIRIT 2013 statement: defining standard protocol items for clinical trials. Ann Intern Med. 2013;158(3):200-7.
25. Janssen TL, Alberts AR, Hooft L, Mattace-Raso F, Mosk CA, van der Laan L. Prevention of postoperative delirium in elderly patients planned for elective surgery: systematic review and meta-analysis. Clin Interv Aging. 2019;14:1095-117.
26. Abraha I, Trotta F, Rimland JM, Cruz-Jentoft A, Lozano-Montoya I, Soiza RL, et al. Efficacy of Non-Pharmacological Interventions to Prevent and Treat Delirium in Older Patients: A Systematic Overview. The SENATOR project ONTOP Series. PLoS One. 2015;10(6):e0123090.
27. Lee H, Ju JW, Oh SY, Kim J, Jung CW, Ryu HG. Impact of timing and duration of postoperative delirium: a retrospective observational study. Surgery. 2018;164(1):137 - 43.
28. Association AP. Diagnostic and Statistical Manual of Mental Disorders, Fourth Edition. May 2013.
29. Gavinski K, Carnahan R, Weckmann M. Validation of the delirium observation screening scale in a hospitalized older population. J Hosp Med. 2016;11(7):494-7.
30. Detroyer E, Clement PM, Baeten N, Pennemans M, Decruyenaere M, Vandenberghe J, et al. Detection of delirium in palliative care unit patients: a prospective descriptive study of the Delirium Observation Screening Scale administered by bedside nurses. Palliat Med. 2014;28(1):79-86.
31. van Velthuijsen EL, Zwakhalen SM, Warnier RM, Mulder WJ, Verhey FR, Kempen GI. Psychometric properties and feasibility of instruments for the detection of delirium in older hospitalized patients: a systematic review. Int J Geriatr Psychiatry. 2016;31(9):974-89.
32. American Psychiatric A. American Psychiatric Association DSM-5 Task Force (2013): Diagnostic and Statistical Manual of Mental Disorders: DSM-5. Washington, DC: American Psychiatric Association.
33. Chen L, Xu M, Li GY, Cai WX, Zhou JX. Incidence, Risk Factors and Consequences of Emergence Agitation in Adult Patients after Elective Craniotomy for Brain Tumor: A Prospective Cohort Study. PLoS One. 2014;9(12):e114239.
34. Flanigan PM, Jahangiri A, Kuang R, Truong A, Choi S, Chou A, et al. Developing an Algorithm for Optimizing Care of Elderly Patients With Glioblastoma. Neurosurgery. 2018;82(1):64-75.
35. Herrero S, Carrero E, Valero R, Rios J, Fabregas N. Postoperative surveillance in neurosurgical patients - usefulness of neurological assessment scores and bispectral index. Rev Bras Anestesiol. 2017;67(2):153-65.
36. Matano F, Mizunari T, Yamada K, Kobayashi S, Murai Y, Morita A. Environmental and Clinical Risk Factors for Delirium in a Neurosurgical Center: A Prospective Study. World Neurosurg. 2017;103:424-30.
37. Wang J, Ji Y, Wang N, Chen W, Bao Y, Qin Q, et al. Establishment and validation of a delirium prediction model for neurosurgery patients in intensive care. Int J Nurs Pract. 2020:e12818.
38. Florez-Perdomo WA, Garcia-Ballestas E, Moscote-Salazar LR, Konar SK, Raj S, Chouksey P, et al. Heart Rate Variability as a Predictor of Mortality in Traumatic Brain Injury: A Systematic Review and Meta-Analysis. World Neurosurg. 2021;148:80-9.
39. Mojtabavi H, Saghazadeh A, Valenti VE, Rezaei N. Can music influence cardiac autonomic system? A systematic review and narrative synthesis to evaluate its impact on heart rate variability. Complement Ther Clin Pract. 2020;39:101162.



For peer review only - http://bmjopen.bmj.com/site/about/guidelines.xhtml

40. Khan SH, Wang S, Harrawood A, Martinez S, Heiderscheit A, Chlan L, et al. Decreasing Delirium through Music (DDM) in critically ill, mechanically ventilated patients in the intensive care unit: study protocol for a pilot randomized controlled trial. Trials. 2017;18(1):574.
41. Kakar E, Ista E, Klimek M, Jeekel J. Implementation of music in the perioperative standard care of colorectal surgery: study protocol of the IMPROVE Study. BMJ Open. 2021;11(10):e051878.

For peer review only

**Figure 1. Trial flowchart**

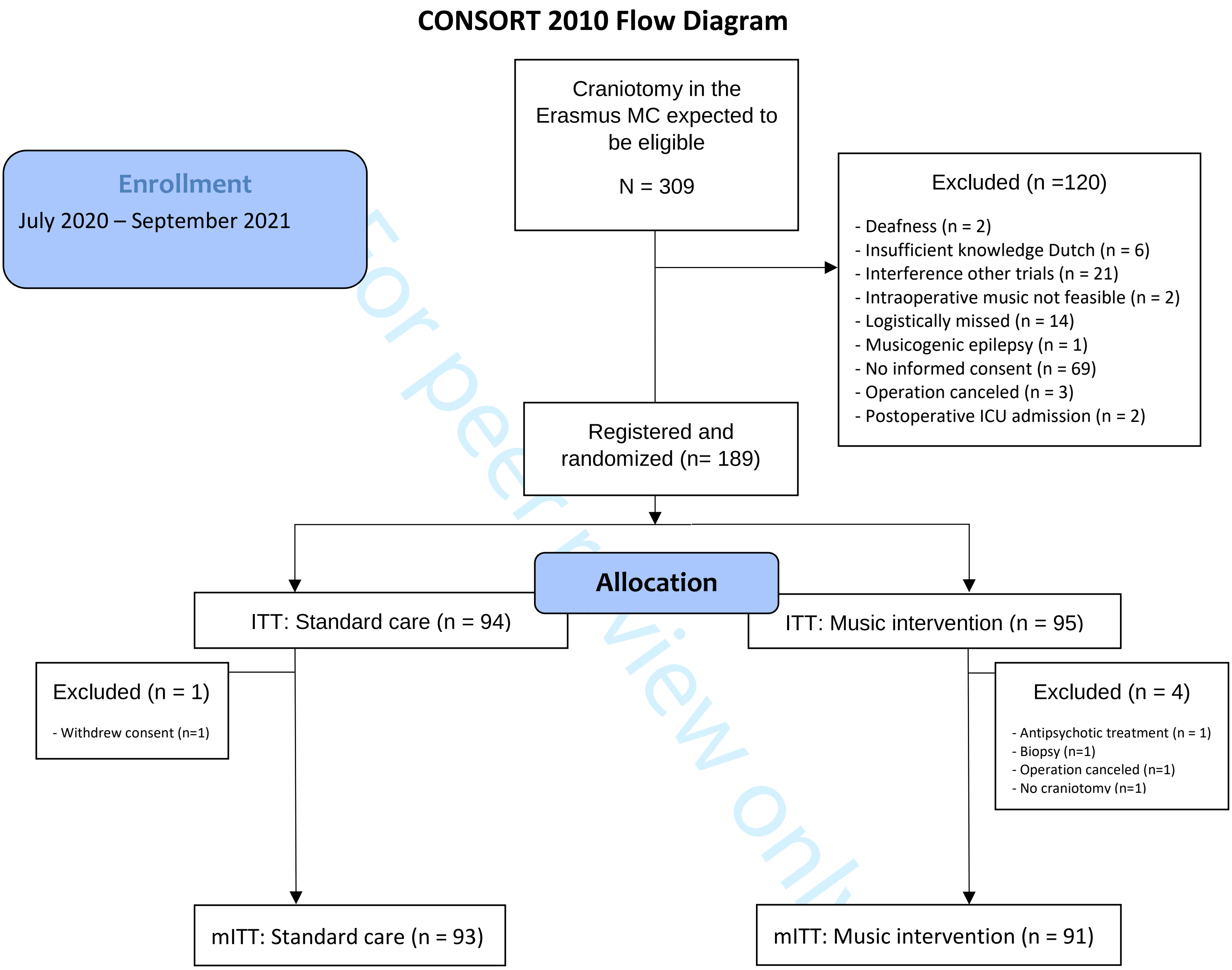

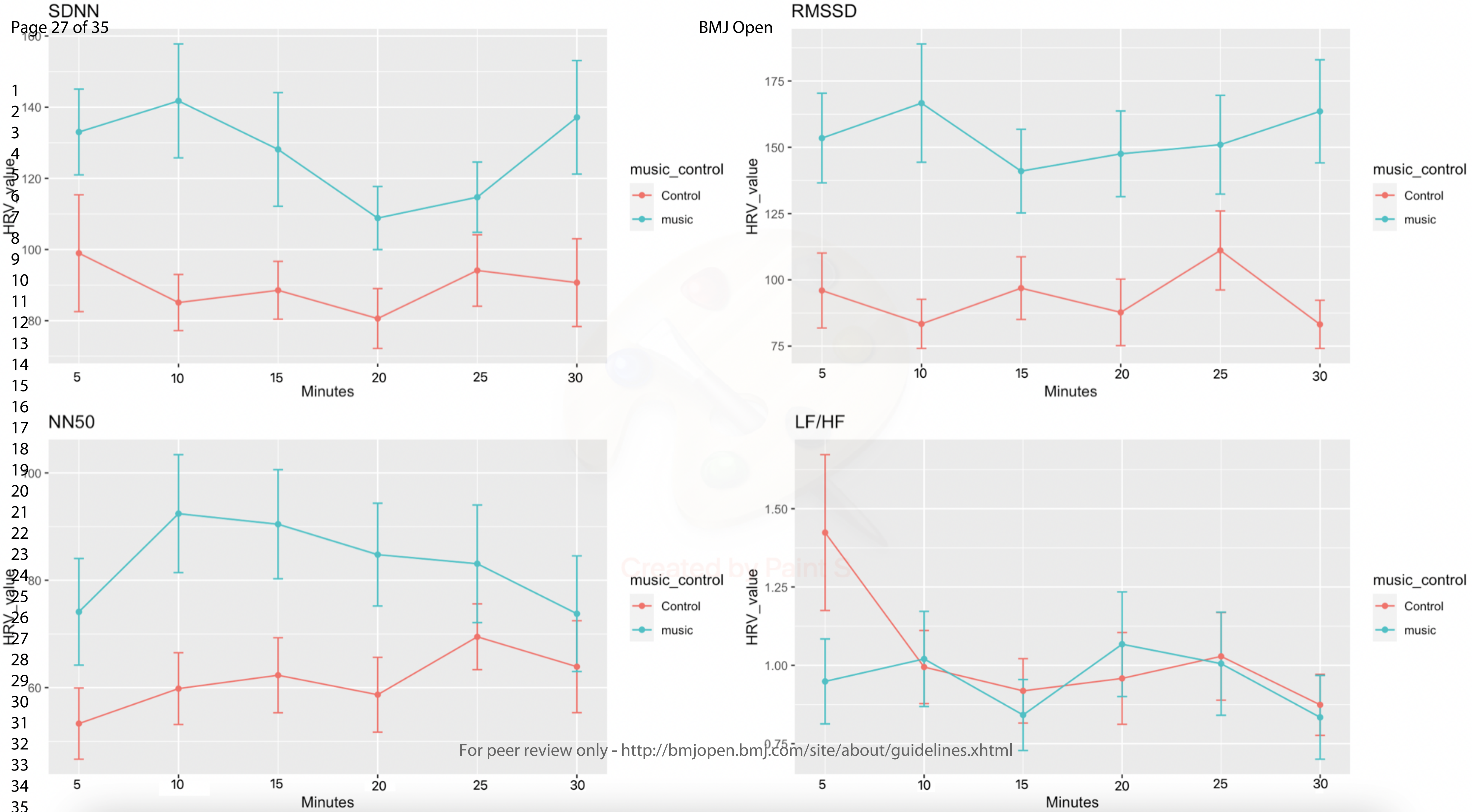
SDNN
RMSSD
NN50
LF/HF
HRV_value
Minutes
music_control
Control
music

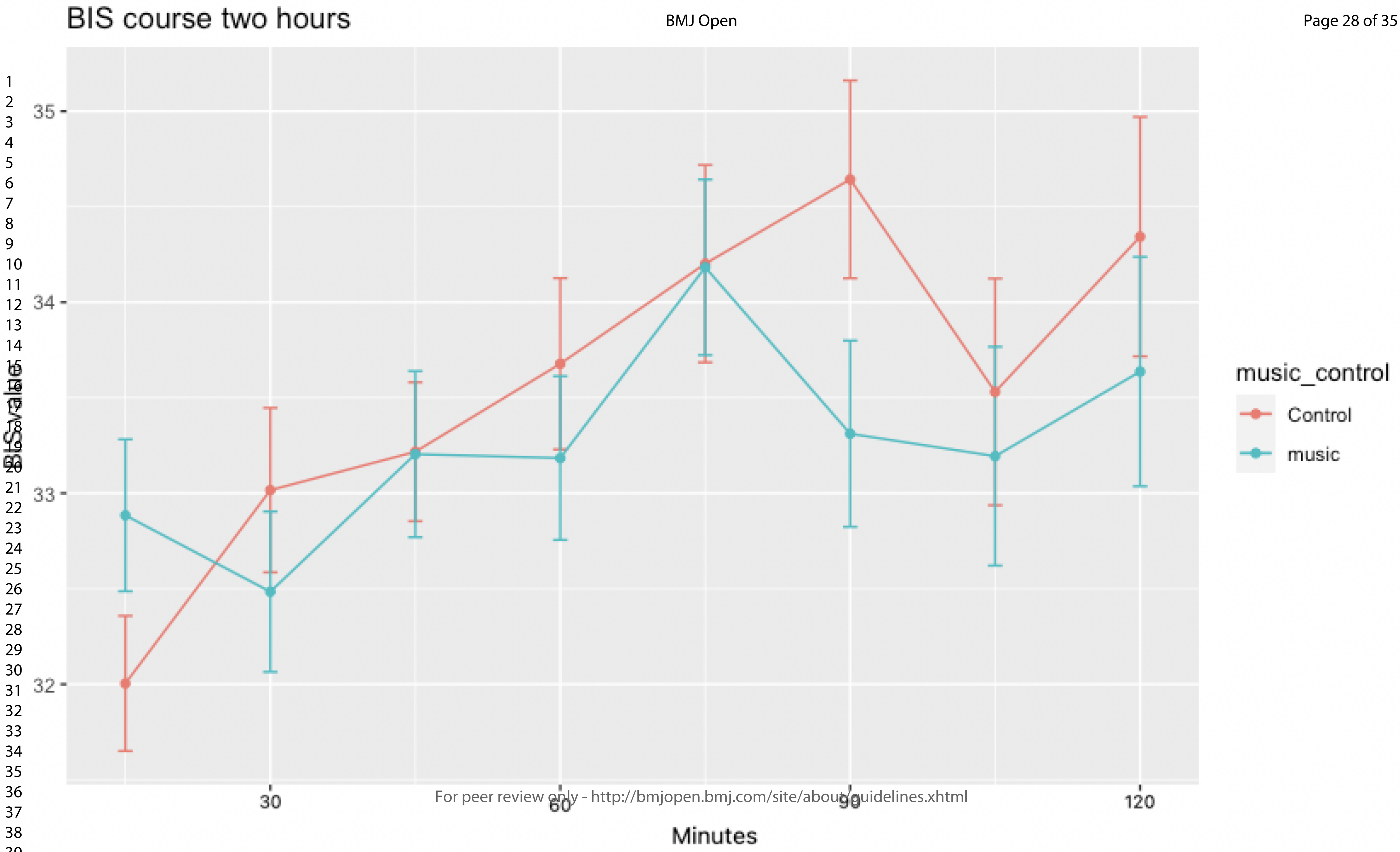
BIS course two hours
BIS value
35
34
33
32
30
60
90
120
Minutes
music_control
Control
music

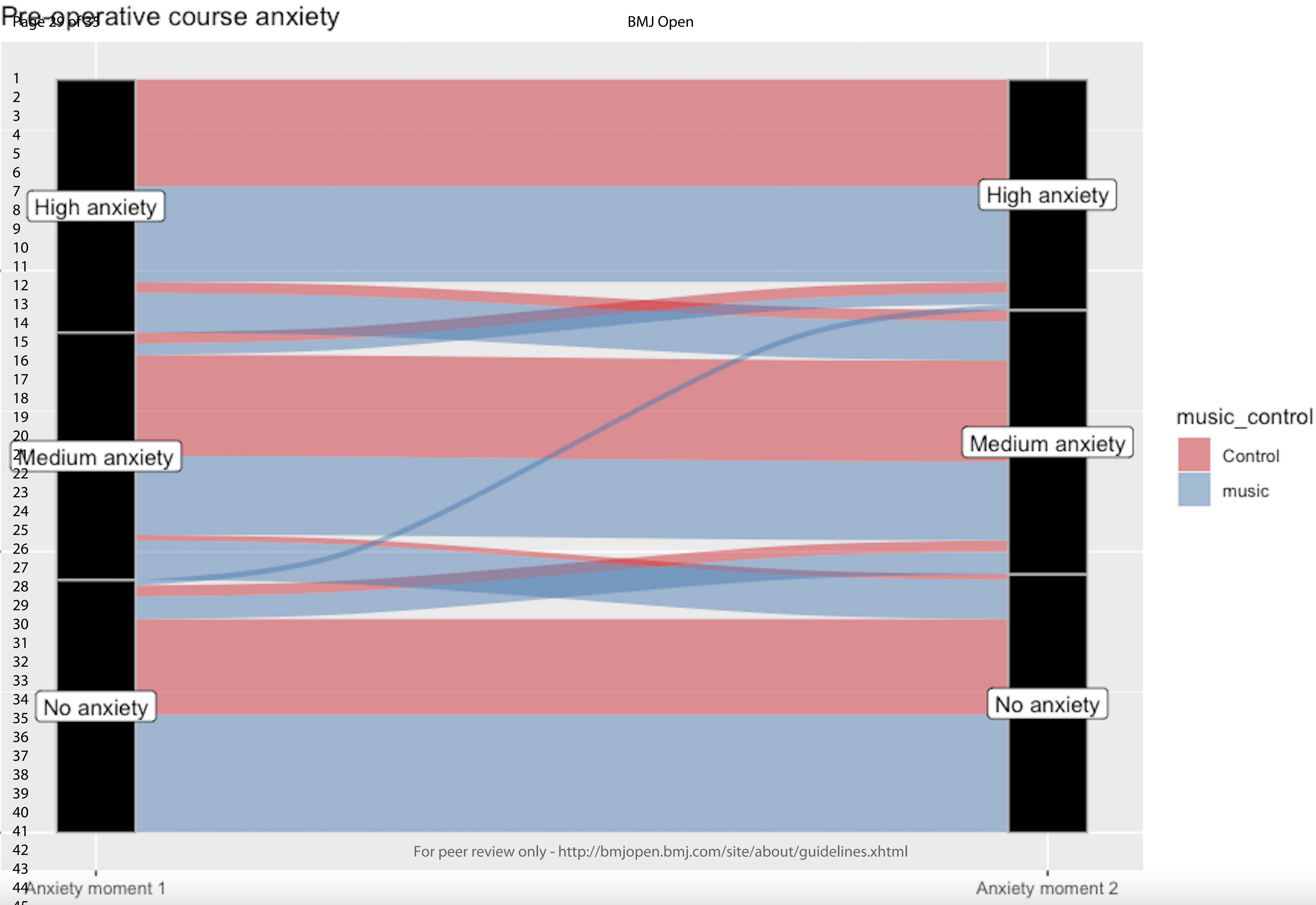
Pre-operative course anxiety
High anxiety
Medium anxiety
No anxiety
High anxiety
Medium anxiety
No anxiety
music_control
Control
music
Anxiety moment 1
Anxiety moment 2

## Supplementary figure 1. Study course MUSYC trial

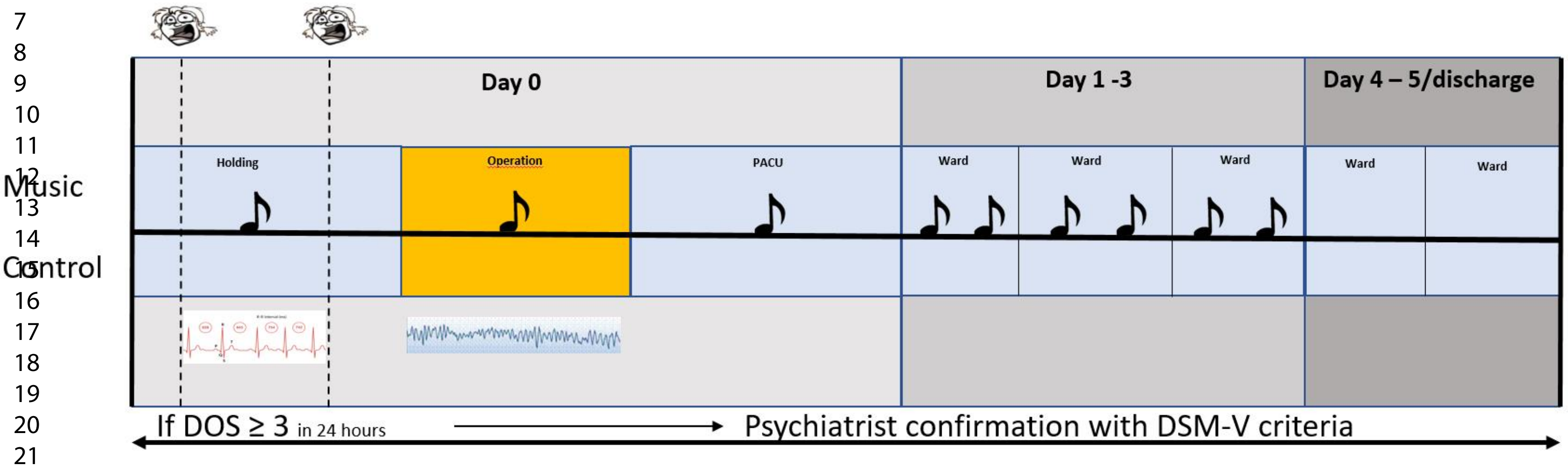


Course trial: after randomization, both groups receive ECG recording and two times anxiety measures. Intra-operative BIS registration is applied for depth of anesthesia. All participating subjects are treated according to standard care and delirium is measured until day 5 or discharge. Participants in the intervention group additionally receive music intervention 30 minutes before surgery, intra-operative during the entire operation, 30 minutes after surgery and twice per day 30minutes until post-operative day 3.

For peer review only

## Supplementary figure 2. Course delirium diagnosis

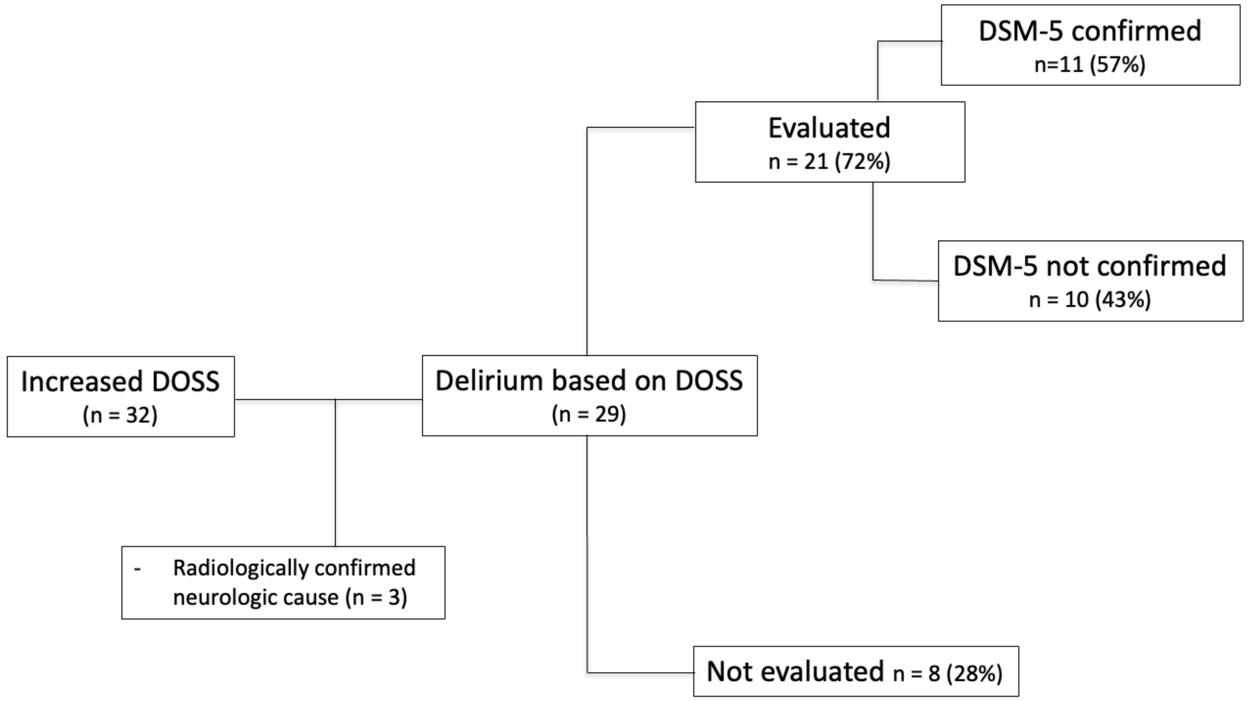


Delirium diagnosis flowchart, diagnosis of delirium required a two-step procedure. First, all participants were screened with the DOS scale. Second, in case of a daily mean score of 3 or higher (and no neurologic cause was radiologically found) a psychiatrist was consulted to assess the clinical diagnosis of delirium based on the DSM-5 criteria. In 8 patients, the psychiatrist was not consulted due to logistic issues.

view only

## Supplementary table 1. Adherence to music intervention during trial

| | Day 0 | | | Day 1 | | Day 2 | | Day 3 | |
|---|---|---|---|---|---|---|---|---|---|
| **Moment[1]** | **Pre** | **Per** | **Post** | **Morning** | **Noon** | **Morning** | **Noon** | **Morning** | **Noon** |
| **N[2]** | 91 | 91 | 91 | 91 | 91 | 88 | 88 | 70 | 70 |
| | | | | | | | | | |
| **Music** | | | | | | | | | |
| **No** | 3 | 0 | 21 | 25 | 26 | 26 | 21 | 19 | 20 |
| **Yes** | 79 | 91 | 61 | 59 | 45 | 44 | 39 | 24 | 18 |
| **Unknown[3]** | 9 | 0 | 9 | 7 | 20 | 18 | 28 | 27 | 32 |
| **Adherence[4]** | 96% | 100% | 74% | 70% | 63% | 63% | 65% | 56% | 47% |

Patients in the mITT population allocated to the music group. 1. Session of 30 minutes in the morning or afternoon. 2. Amount of patients in the musical group not discharged from the ward. 3. Unregistered music session. 4. Adherence calculated from the registered patients (i.e. 'Yes'/'No' + 'Yes')

For peer review only

# 2017 CONSORT checklist of information to include when reporting a randomized trial assessing nonpharmacologic treatments (NPTs)*. Modifications of the extension appear in italics and blue.

| Section/Topic Item | Checklist item no. | CONSORT item | Extension for NPT trials | Page |
|---|---|---|---|---|
| **Title and abstract** | | | | |
| | 1a | Identification as a randomized trial in the title | | 1 |
| | 1b | Structured summary of trial design, methods, results, and conclusions (for specific guidance see CONSORT for abstracts) | *Refer to CONSORT extension for abstracts for NPT trials* | *3* |
| **Introduction** | | | | |
| Background and objectives | 2a | Scientific background and explanation of rationale | | 4, 5 |
| | 2b | Specific objectives or hypotheses | | 4, 5 |
| **Methods** | | | | |
| Trial design | 3a | Description of trial design (such as parallel, factorial) including allocation ratio | When applicable, how care providers were allocated to each trial group | 6:10 |
| | 3b | Important changes to methods after trial commencement (such as eligibility criteria), with reasons | | 6, 8 |
| Participants | 4a | Eligibility criteria for participants | When applicable, eligibility criteria for centers and for *care providers* | 6 |
| | 4b | Settings and locations where the data were collected | | 6 |
| Interventions † | 5 | The interventions for each group with sufficient details to allow replication, including how and when they were actually administered | Precise details of both the experimental treatment and comparator | 7, 8 |
| | 5a | | Description of the different components of the interventions and, when applicable, description of the procedure for tailoring the interventions to individual participants. | 7, supplementary figures |
| | 5b | | Details *of whether and* how the interventions were standardized. | 7, supplementary figures |
| | 5c. | | Details *of whether and* how adherence of care providers to the protocol was assessed or enhanced | 8, supplementary figures |

***Cite as:*** *Boutron I, Altman DG, Moher D, Schulz KF, Ravaud P. CONSORT Statement for Randomized Trials of Nonpharmacologic Treatments: A 2017 Update and a CONSORT Extension for Nonpharmacologic Trial Abstracts. Annals of Internal Medicine. 2017 Jul 4;167(1):40–7.*

| Section/Topic Item | Checklist item no. | CONSORT item | Extension for NPT trials | Page |
|---|---|---|---|---|
| | 5d | | *Details of whether and how adherence of participants to interventions was assessed or enhanced* | 8 |
| Outcomes | 6a | Completely defined pre-specified primary and secondary outcome measures, including how and when they were assessed | | **8, 9** |
| | 6b | Any changes to trial outcomes after the trial commenced, with reasons | | **7, 9** |
| Sample size | 7a | How sample size was determined | When applicable, details of whether and how the clustering by care providers or centers was addressed | 9, 10 |
| | 7b | When applicable, explanation of any interim analyses and stopping guidelines | | NA |
| Randomization: | | | | 6, 7 |
| - Sequence generation | 8a | Method used to generate the random allocation sequence | | 6, 7 |
| | 8b | Type of randomization; details of any restriction (such as blocking and block size) | | 6, 7 |
| - Allocation concealment mechanism | 9 | Mechanism used to implement the random allocation sequence (such as sequentially numbered containers), describing any steps taken to conceal the sequence until interventions were assigned | | 6, 7 |
| - Implementation | 10 | Who generated the random allocation sequence, who enrolled participants, and who assigned participants to interventions | | 6, 7 |
| Blinding | 11a | If done, who was blinded after assignment to interventions (for example, participants, care providers, those assessing outcomes) and how | If done, who was blinded after assignment to interventions (e.g., participants, care providers, *those administering co-interventions,* those assessing outcomes) and how | 8 |
| | 11b | If relevant, description of the similarity of interventions | If blinded, method of blinding and description of the similarity of interventions | NA |
| | 11c | | *If blinding was not possible, description of any attempts to limit bias* | 8 |

***Cite as:*** *Boutron I, Altman DG, Moher D, Schulz KF, Ravaud P. CONSORT Statement for Randomized Trials of Nonpharmacologic Treatments: A 2017 Update and a CONSORT Extension for Nonpharmacologic Trial Abstracts. Annals of Internal Medicine. 2017 Jul 4;167(1):40–7.*

| Section/Topic Item | Checklist item no. | CONSORT item | Extension for NPT trials | Page |
|---|---|---|---|---|
| Statistical methods | 12a | Statistical methods used to compare groups for primary and secondary outcomes | When applicable, details of whether and how the clustering by care providers or centers was addressed | 9, 10 |
| | 12b | Methods for additional analyses, such as subgroup analyses and adjusted analyses | | 9, 10 |
| **Results** | | | | |
| Participant flow (a diagram is strongly recommended) | 13a | For each group, the numbers of participants who were randomly assigned, received intended treatment, and were analyzed for the primary outcome | The number of care providers or centers performing the intervention in each group and the number of patients treated by each care provider or in each center | 11, figure 1 |
| | 13b | For each group, losses and exclusions after randomization, together with reasons | | 11, figure 1 |
| | 13c | | *For each group, the delay between randomization and the initiation of the intervention* | *Supplementary figures* |
| | new | | Details of the experimental treatment and comparator as they were implemented | *Supplementary figures* |
| Recruitment | 14a | Dates defining the periods of recruitment and follow-up | | **11, figure 1** |
| | 14b | Why the trial ended or was stopped | | **11** |
| Baseline data | 15 | A table showing baseline demographic and clinical characteristics for each group | When applicable, a description of care providers (case volume, qualification, expertise, etc.) and centers (volume) in each group. | Table 1 |
| Numbers analyzed | 16 | For each group, number of participants (denominator) included in each analysis and whether the analysis was by original assigned groups | | 11, 12, Table 1 |
| Outcomes and estimation | 17a | For each primary and secondary outcome, results for each group, and the estimated effect size and its precision (such as 95% confidence interval) | | 11, 12, table 2, 3 and figure 2, 3 and 4 |
| | 17b | For binary outcomes, presentation of both absolute and relative effect sizes is recommended | | 11, 12, ttable 2, 3 and figure 2, 3 and 4 |
| Ancillary analyses | 18 | Results of any other analyses performed, including subgroup analyses and adjusted analyses, distinguishing pre-specified from exploratory | | 11, 12, table 2, 3 and figure 2, 3 and 4 |

***Cite as:*** *Boutron I, Altman DG, Moher D, Schulz KF, Ravaud P. CONSORT Statement for Randomized Trials of Nonpharmacologic Treatments: A 2017 Update and a CONSORT Extension for Nonpharmacologic Trial Abstracts. Annals of Internal Medicine. 2017 Jul 4;167(1):40–7.*

| Section/Topic Item | Checklist item no. | CONSORT item | Extension for NPT trials | Page |
|---|---|---|---|---|
| Harms | 19 | All important harms or unintended effects in each group (for specific guidance see CONSORT for harms) | | NA |
| **Discussion** | | | | |
| Limitations | 20 | Trial limitations, addressing sources of potential bias, imprecision, and, if relevant, multiplicity of analyses | In addition, take into account the choice of the comparator, lack of or partial blinding, and unequal expertise of care providers or centers in each group | 3, 15 |
| Generalizability | 21 | Generalizability (external validity, applicability) of the trial findings | Generalizability (external validity) of the trial findings according to the intervention, comparators, patients, and care providers and centers involved in the trial | 13-15 |
| Interpretation | 22 | Interpretation consistent with results, balancing benefits and harms, and considering other relevant evidence | | 13-15 |
| **Other information** | | | | |
| Registration | 23 | Registration number and name of trial registry | | 6 |
| Protocol | 24 | Where the full trial protocol can be accessed, if available | | 6 |
| Funding | 25 | Sources of funding and other support (such as supply of drugs), role of funders | | 16 |

**Additions or modifications to the 2010 CONSORT checklist. CONSORT = Consolidated Standards of Reporting Trials*
*†The items 5, 5a, 5b, 5c, 5d are consistent with the Template for Intervention Description and Replication (TIDieR) checklist*

***Cite as:*** *Boutron I, Altman DG, Moher D, Schulz KF, Ravaud P. CONSORT Statement for Randomized Trials of Nonpharmacologic Treatments: A 2017 Update and a CONSORT Extension for Nonpharmacologic Trial Abstracts. Annals of Internal Medicine. 2017 Jul 4;167(1):40–7.*

For peer review only - http://bmjopen.bmj.com/site/about/guidelines.xhtml

For peer review only
